# Scalable high-throughput microfluidic separation of magnetic microparticles

**DOI:** 10.1016/j.device.2024.100403

**Published:** 2024-07-19

**Authors:** Hongri Gu, Yonglin Chen, Anton Lüders, Thibaud Bertrand, Emre Hanedan, Peter Nielaba, Clemens Bechinger, Bradley J. Nelson

**Affiliations:** 1 Department of Physics, University of Konstanz, Konstanz 78464, Germany; 2 Institute of Robotics and Intelligent Systems, ETH Zurich, Zurich CH-8092, Switzerland

## Abstract

Surface-engineered magnetic microparticles are used in chemical and biomedical engineering due to their ease of synthesis, high surface-to-volume ratio, selective binding, and magnetic separation. To separate them from fluid suspensions, existing methods rely on the magnetic force introduced by the local magnetic field gradient. However, this strategy has poor scalability because the magnetic field gradient decreases rapidly as one moves away from the magnets. Here, we present a scalable high-throughput magnetic separation strategy using a rotating permanent magnet and two-dimensional arrays of micromagnets. Under a dynamic magnetic field, nickel micromagnets allow the surrounding magnetic microparticles to self-assemble into large clusters and effectively propel themselves through the flow. The collective speed of the microparticle swarm reaches about two orders of magnitude higher than the gradient-based separation method over a wide range of operating frequencies and distances from a rotating magnet.

## Highlights

• A scalable system to remove magnetic microparticles from liquid
• A conceptual shift from “microfluidic-centered” to “magnet-centered” system
• Micromagnets and a rotating magnetic field are used to boost collective movement
• Theoretical framework to compare the separation throughput using different methods

## The bigger picture

Reliable and effective removal of magnetic particles is a critical step in many emerging high-throughput applications. The required throughput for these applications can be orders of magnitude larger than today’s microfluidic separation systems. Here, we propose a scalable and high-throughput microfluidic separation method. We discuss the underlying mechanism of collective transport of microparticles under flow, evaluate its unique advantages from a scalability perspective, and provide a roadmap for further optimization and parallel scaling to expand the capability of high-throughput removal of magnetic particles from liquid. The results pave the way for emerging applications of magnetic micro- and nanoparticles that require significantly higher throughput, including blood purification, water remediation, compact chemical reactors, and other scenarios of magnetic micro- and nanorobots that need to be recovered, recycled, and reused.

## Graphical abstract

**Figure:**
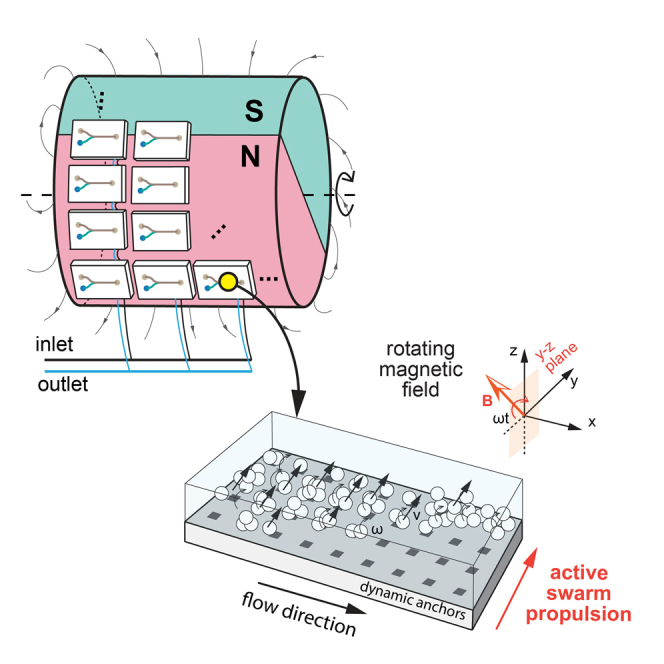

## Teaser

This work presents a method for rapid and efficient separation of magnetic microparticles in liquids. With very high-throughput applications in mind, we focus on the scalability of the magnetic separation systems. We propose a conceptual evolution from a “"microfluidic-centric” to a “magnet-centric” system architecture and combine micromagnet arrays and rotating magnetic fields to increase separation efficiency. This work provides an alternate path toward high-throughput magnetic separation systems, which are critical for blood purification, micro- and nanorobot recycling, and wastewater remediation.

## Introduction

Magnetic micro- and nanoparticles with sizes ranging from hundreds of nanometers to hundreds of micrometers hold promise across a wide array of applications, from environmental remediation^1^ to biomedical engineering.^2^ These particles have many unique properties that make them suitable for various applications, including high surface-to-volume ratios, high surface loading capacities, chemical stability, and biocompatibility. In recent years, they have become a popular platform technology where the surface can be coated with functional materials that expand their application scenarios, such as catalysts for accelerating specific chemical reactions,^3^^,^^4^ ligands for selective binding of large biomolecules and cells,^5^ and active sites for the adsorption of heavy metals in the environment.^6^ In addition, magnetic particles can be driven to form active swarms to enhance collective functions such as virus detection,^7^^,^^8^^,^^9^^,^^10^ and DNA scavenging.^11^^,^^12^^,^^13^ In single-channel microfluidic devices, magnetic particles are typically injected from a storage channel, sufficiently mixed with the target fluid, and then magnetically separated and collected for further analysis. These microfluidic systems are widely used for immunoassay and the detection of specific molecules, with typical flow rates of a few milliliters per minute and sample volumes in the range of nanoliters to milliliters.^14^^,^^15^

Thanks to the developments in particle synthesis and functionalization, the available selection of magnetic microparticles has expanded, and the associated costs are continuously decreasing. This makes magnetic microparticles a viable option for many emerging applications that require much higher throughput (at least ∼100 mL/min) compared to single-channel microfluidic devices. For example, in blood purification, magnetic microparticles can be used to selectively eliminate pathogens in acute blood infections (e.g., sepsis) where traditional antibiotic treatment is too slow.^16^^,^^17^^,^^18^ With β-amyloid binders on the surface, magnetic microparticles can be used to remove toxic protein aggregates from the blood of patients with Alzheimer’s disease.^19^^,^^20^ In another example, researchers are exploring compact solutions with microfluidic devices for hospital wastewater treatment, where various pharmaceutically active compounds must be broken down before they enter the drainage system.^21^^,^^22^ Magnetic micro- and nanoparticles inside a small system can be promising for such applications where industrial solutions are too large to fit.^1^^,^^22^^,^^23^ These envisioned applications rely on robust separation methods to effectively and reliably recover magnetic microparticles at the end of the process, which is often the limiting factor for magnetic-particle-based applications.^24^ In some cases, this can be critical, as unrecovered magnetic particles can cause damage to the human body and the environment (e.g., blocking distal blood vessels, degrading to microplastics, and being ingested by marine animals).

**Figure 1:**
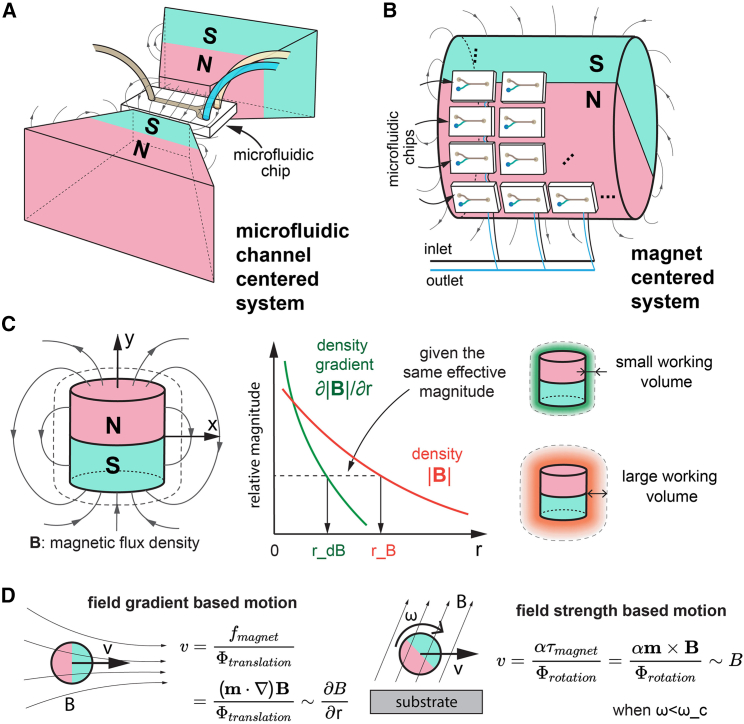
Concept and analysis of microfluidic-centered and magnet-centered systems for magnetic particle separation (A and B) Illustration of a typical microfluidic-centered system and a magnet-centered system, respectively. The magnetic flux density maps around a typical cylindrical magnet. (C) The field gradient dB/dx decreases faster than the magnetic flux density B away from the magnet. For the same relative magnitude, the gradient-based separation methods have a much smaller working volume than the field-strength-based separation methods. The working volumes for the two different methods are shown as colored shadows around the same magnet. (D) Two examples of gradient-based motion (a magnet in a nonuniform magnetic field) and field-strength-based motion (a rolling sphere near a surface). $f_{magnet}$ and $\tau_{magnet}$ are the force and torque applied on the mobile magnetic microparticle, and $\Phi_{translation}$ and $\Phi_{rotation}$ are translational and rotational drag coefficients, respectively. α is the coupling factor of the translation and rotation near a surface. See the supplemental information for more details on the comparison.

Most existing microfluidic separation methods rely on the magnetic force due to the local field gradient ($\vec{F}=\left(\;\vec{m}\cdot \nabla \right)\;\vec{B}$), where $\vec{m}$ is the dipole moment of the magnetic microparticle and $\vec{B}$ is the density flux of the magnetic field. In a simple example, one can place a bar magnet close to the microfluidic channel, and magnetic microparticles will drift and accumulate toward the north and south poles, where the magnetic field gradient is greatest.^17^^,^^25^^,^^26^^,^^27^^,^^28^^,^^29^ This principle guides the design of most microfluidic magnetic microparticle separation devices, resulting in a microfluidic-centered design where the microfluidic channel is surrounded by strong and bulky magnets (Figure 1A). Such systems can achieve very good separation results with a typical throughput of a few milliliters per minute,^15^^,^^27^^,^^30^^,^^31^^,^^32^^,^^33^ which is sufficient for most diagnostic-oriented applications (e.g., counting circulating tumor cells^15^). However, many emerging applications require significantly higher separation throughput, and such a system cannot be easily scaled to meet these needs. In the blood purification example, the ideal throughput is similar to that of a hemodialysis machine (approximately 200–500 mL/min), which is almost two orders of magnitude higher than the current state-of-the-art microfluidic separation systems.^34^^,^^35^^,^^36^

Increasing separation throughput by orders of magnitude is a challenging problem. Scaling up the dimensions of the microfluidic channel dramatically reduces the effective magnetic field gradient.^37^^,^^38^^,^^39^ In addition, scaling out (i.e., increasing the number in parallel) of the same-size microfluidic devices requires a corresponding number of large magnets, where both the overall size and cost of the system become prohibitive. Many high-throughput systems have been reported in the literature, often with different microfluidic channel designs, different magnetic microparticles (ferromagnetic, paramagnetic, etc.), different magnet designs (electromagnet, permanent magnet), and different working fluids (viscous, viscoelastic, and even some biofluids).^4^^,^^31^^,^^40^ To date, there is no general framework for the development of very high-throughput magnetic microfluidic systems in which all these reported systems can be fairly compared, analyzed, and evaluated.^41^^,^^42^ It remains difficult for researchers to strategically combine the advantages of different existing separation systems and to develop new devices for new applications that may require orders of magnitude higher throughput.

In this article, we first analyze existing microfluidic separation methods from the perspective of throughput scalability and then introduce and discuss the experimental results of dynamic transport of swarming particles. We find that the inefficient use of the magnet is the main bottleneck for scalable magnetic separation. Based on this analysis, we constrain our design with a fixed magnet volume and try to use its surrounding magnetic field for separation as efficiently as possible. As a result, we obtain a magnet-centered separation system, which is different from the microfluidic-centered systems commonly found in the literature. In addition, we propose a novel separation method that combines the use of a micromagnet array and a rotating magnetic field. The high magnetic field gradient near the micromagnets attracts nearby magnetic microparticles, assembles them into large clusters, and helps them to “jump” on the micromagnet grid under a rotating magnetic field. This collective transport enables the rapid separation of magnetic particles in microfluidic channels, even in the presence of strong fluid flow. Combining experiments and numerical simulations, we studied the collective dynamics under different magnetic field strengths (18–130 mT) and rotation frequencies (1–130 Hz). We discover three distinct states of collective transport and quantify the key parameters (e.g., particle density and the width of the particle band) during the transition between the collective states. Finally, we show that this collective transport is an effective method for the separation of magnetic microparticles in water and in porcine blood, with the possibility of being adapted to various high-throughput applications.

### Design strategy

#### Microfluidic-centered vs. magnet-centered systems

Microfluidic devices, together with external control modules, enable precise fluid manipulation (e.g., rapid mixing and reactions) in continuous flows or within droplets.^33^ Using soft lithography and three-dimensional (3D) printing, microfluidic channels can be integrated and scaled in parallel to increase throughput while maintaining the benefits of channel size.^32^ However, this is not the case for most magnetic separation systems, where bulky magnets are placed closely around the microfluidic channels to maximize the magnetic field gradient.^40^^,^^43^ In some cases, multiple bulky magnets are implemented around the microfluidic channel in all directions. Increasing the throughput in these microfluidic systems also means increasing the number of accompanying bulky magnets. This will inevitably increase rapidly the size and cost of the scaled system, especially considering that the target throughput can be orders of magnitude higher than that of a standard microfluidic channel. In these scaled-up designs, the magnet, rather than the microfluidics, becomes the bottleneck for scaling because the use of magnets is inefficient.

To overcome this limitation of scalability and to further increase throughput, we propose the concept of “magnet-centered” systems, as shown in Figure 1B. In this concept, we assume that the available magnet has a fixed volume and that the microfluidic channels are arranged around this magnet. As a result, we can no longer add more magnets around the microfluidic channel to increase the local magnetic field gradient, as in the case of microfluidic-centered systems. This conceptual change forces us to think about how to use the magnetic field around this magnet as efficiently as possible and how to design microfluidic separation systems. For example, the gradient (∂B/∂r, where r corresponds to the distance from the magnet) decreases more steeply than the magnetic field B (in point dipole approximation: $\vec{B}\left(r\right)=\frac{\mu_{0}}{4\pi }\frac{3\hat{\vec{r}}\left(\hat{\vec{r}}\cdot \vec{m}\right)-\vec{m}}{{\left|\vec{r}\right|}^{3}}$, where $\vec{r}$ is a vector from the center of the magnetic dipole to the location of interest, $\vec{m}$ is the magnetic dipole moment, $\mu_{0}$ is the vacuum permeability, and the hat means that a vector is normalized) in the space around a magnet. The magnetic field decreases at the rate of ${\left|\vec{r}\right|}^{-3}$, and the gradient decreases at the rate of ${\left|\vec{r}\right|}^{-4}$ when the magnet is considered a point dipole.^38^

This observation raises an important question about the available working volume around a given magnet. If we choose a minimum value for the magnetic field gradient and magnetic flux density that can be used for magnetic particle separation, we will find that the available working volume, where we can place microfluidic separation channels, is different for both cases. The working volume of the gradient-based method (marked green in Figure 1C) is much smaller than the working volume of the flux-based method (marked red in Figure 1C). Regardless of the actual separation efficiency, the available working volume alone makes a significant difference in terms of the throughput because more microfluidic channels and devices can fit into a larger working volume. In Figure 1D, we select two classical examples to move magnetic particles in a laminar flow. One is to use the magnetic force due to the magnetic field gradient, and the other is to use a magnetic torque under a rotating magnetic field. In this case, the magnetic-torque-based method, which depends on the magnetic flux density, is likely to have a larger available working volume than the gradient-based separation methods.

#### Combining micromagnets and dynamic magnetic fields

**Figure 2:**
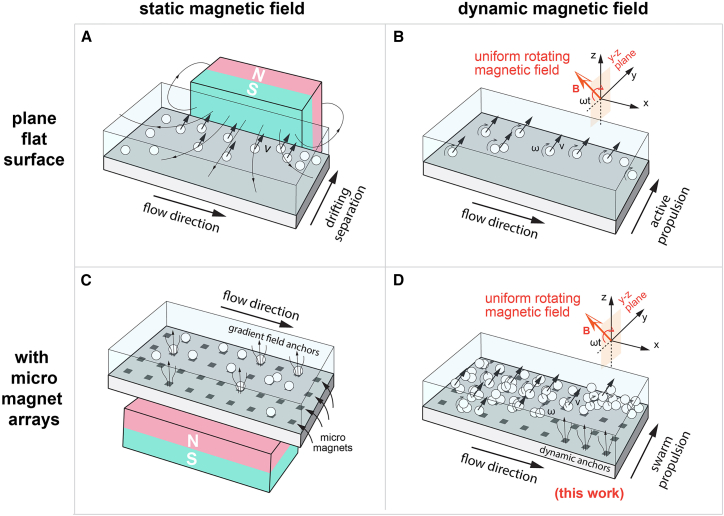
Four categories of magnetic particle separation devices using microfluidics (A–D) The four categories are determined based on two independent criteria: (1) use of a static or a dynamic magnetic field or (2) use of an array of micromagnets or simply a flat surface in the microfluidic channel. In this work, we combined both a dynamic rotating magnetic field and micromagnets to achieve better dynamic locomotion of magnetic microparticle swarms through microfluidic flow.

Among the gradient-based magnetic separation systems, one popular technique to further enhance the magnetic field gradient is to use micromagnets.^30^ Patterned micromagnets can be deposited directly on the substrate as part of the microfluidic channel, placing them very close to the target magnetic microparticles. The strong gradient field around the micromagnets creates many magnetic field “hotspots” on the microfluidic chip. The effective magnetic force is so strong that it can be used to trap cells and large particle clusters.^44^ In the bottom column of Figure 2, the magnetic flux lines are drawn to demonstrate such gradient fields and their effect on magnetic microparticles.

Another method that can increase the separation efficiency is to use a dynamic magnetic field, which is very popular in the microrobotics community. For example, a rotating magnetic field can apply a continuous torque to magnetic particles, propelling them forward as they rotate (e.g., surface roller and helix).^10^^,^^39^^,^^45^ The speed of the moving microparticle depends on the rotational frequency and the field strength of the applied magnetic field. The magnetic particle is synchronized at low frequencies and slows down at high frequencies when the fluid drag is high. At a moderate magnetic field strength of 10 mT (which is much lower than the magnetic field strength for most separation devices), the particle can reach 10–100 body lengths per second in water,^46^ which is typically higher than the speed we observed in gradient-field-based microfluidic separation devices. It is also known in the microrobotics community that for the same magnetic setup that can generate both a gradient field and a uniform rotating magnetic field, e.g., Octomag,^47^ the rotating magnetic field can move particles much faster than the gradient-based method.^39^ However, such systems can be costly and complicated compared to a single piece of magnet for magnetic particle separation purposes.

In Figure 2, we classify magnetic separation devices into four categories based on two independent criteria: (1) the use of a static magnetic field (Figures 2A and 2C) or a dynamic field (Figures 2B and 2D) and (2) the use of an array of micromagnets (Figures 2C and D) or simply a flat surface inside the microfluidic channel (Figures 2A and 2B). In this work, we combined both a dynamic magnetic field (i.e., a rotating magnetic field) and patterned micromagnets to achieve fast and collective transport of magnetic microparticles across the microfluidic flow. As shown in the 2D case,^48^ the magnetic particle swarm can self-assemble into large clusters and move collectively, bridging the particles and achieving a much higher transport speed. The two methods work together in our experiments and show superior performance compared to other magnetic separation devices, which we explain later in the discussion.

#### Scalability and cost considerations

Implementing micromagnets and a rotating magnetic field can be expensive and may not be easily accessible to researchers in microfluidics and magnetic separation, as magnetic actuation systems are usually large and costly. These systems are usually composed of multiple electromagnets or permanent magnets that allow individual control to generate a calibrated magnetic field in the workspace.^47^^,^^49^ This is critical for controlling the precise motion of the microrobot but is redundant for the purpose of magnetic particle separation. We use a simple magnetic setup that can be scaled to meet the throughput requirements in different applications. The setup is based on a permanent magnet rotating at a constant speed. Using a stepper motor and a motor controller, the speed of the rotating magnetic field can be easily controlled, which is less expensive than coil-based electromagnetic systems.

**Figure 3:**
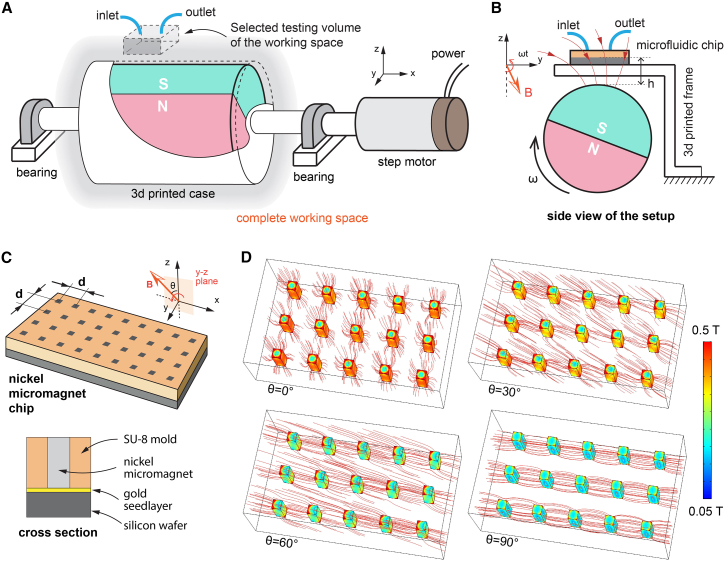
Experimental setup of a rotating magnetic field and the magnetic field in the vicinity of the micromagnet array (A) An illustration of the experimental setup for scalable separation and the selected working volume of the available field, which can be further scaled. (B) Side view of the setup showing the relative z-position between the rotating magnet, the 3D-printed frame, and the micromagnet chip in the microfluidic device. (C) Structure of the nickel micromagnet chip. The periodicity d is equal to 60 μm in the x and y directions. The detailed fabrication process can be found in the supplemental information. (D) Results of numerical simulations of the magnetic field around the micromagnet array. The orange lines indicate the local magnetic field directions and the relative density near the micromagnets. The colored area shows the magnetic flux density on the surface of the nickel micromagnets in the range of 50 mT to 0.5 T under an external uniform field of 200 mT. See the supplemental information for more details of the simulation.

As depicted in Figure 3A, we use a cylindrical NdFeB magnet (grade: N35, diameter: 3 cm, length: 4 cm, surface magnetic flux density: 439 mT) with the dipole direction along the radial axis. The magnetic field strength measured at a distance from the surface of the magnet is shown in Figure S3. The NdFeB magnet is fixed inside a 3D-printed case, supported by two bearings, and connected to the motor by a coupling (the exact structure and computer-aided design [CAD] data of the actual setup can be found in Figures S1 and S2). The dynamic load on the motor is quite small, since the interaction with the magnetic particles can be negligible compared to the friction in the system. It is worth noting that the rotating magnetic field strength at any given position around this dipole magnet is not constant. Due to the symmetry of the dipole magnet in 3D, the magnetic field is stronger when facing the north and south poles and weaker in between, as shown in Figure S4. We neglect these differences and use the average field strength to represent the rotating magnetic field.

Inside the microfluidic channel, we fabricated the micromagnet array using nickel and SU-8 photoresist on a silicon chip. As shown in Figure 3C, the nickel micromagnets are embedded in the SU-8 layer with the same height (45 μm). The processing involves standard microfabrication with 1-step photolithography and nickel electroplating, both of which can be mass produced at a reasonable cost. Electroplated nickel exhibits typical soft magnetic properties with low coercivity and a linear response under an external magnetic field.^48^ The micromagnet chip can be further improved with higher permeability magnetic materials (e.g., NiCo), optimized geometry, and better surface treatment to prevent unwanted particle adhesion and sorption. We used polystyrene-based microparticles (average diameter of 10 μm) with embedded superparamagnetic iron oxide nanoparticles (SPIONs). In this paper, we have only implemented a small microfluidic device using a fraction of the working volume of the rotating magnet to understand collective transport. One can design different setups to accommodate more microfluidic channels and chips to increase the overall throughput depending on the applications.

## Results

### Collective transport on a nickel micromagnet chip

**Figure 4:**
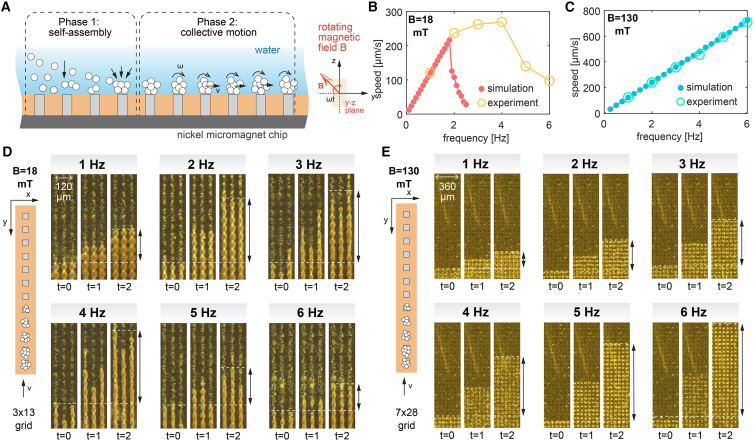
Locomotion speed of the magnetic particle swarm on the chip without externally driven flow (A) Two distinct phases of magnetic microparticle motion: self-assembly through magnetic attractions and collective swarming motion. (B and C) Measured velocity of the front line of the magnetic swarm. (D and E) Time-lapse images of swarm particles moving under a uniform rotating magnetic field with different frequencies of the driving magnetic field. Time unit is seconds.

We observed that the magnetic microparticles interact with the nickel micromagnet chip in two distinct phases (Figure 4A). In the first phase, the magnetic particles are attracted to the surface of the micromagnet chip from different heights due to the very high local magnetic field gradient near the micromagnets (as shown in Figure 3D). The time needed for the particles to reach the surface depends on their vertical positions. The gradient field around the micromagnets has a typical characteristic length of the micromagnet periodicity (d = 60 μm). When the magnetic microparticles are far away from the micromagnets, the gradient field is small and may never bring them close enough to the chip. This indicates that the design of the microfluidic channel requires an optimal channel height. If the channel is too high, the magnetic particles will not be in the magnetic field of the micromagnet chip and will simply flow away; and if the channel is too low, the flow rate will be too low with a very high flow resistance.

In the second phase, magnetic microparticles are on the surface of the magnetic microchip, and the behavior is dominated by the embedded micromagnet array. These microparticles assemble into large clusters due to the local magnetic field gradient and dynamically transfer from one micromagnet to a neighboring one, achieving collective transport. The local distortions of the magnetic field result in alternating gradient regions because the micromagnets are dynamically magnetized by the rotating magnetic field.^48^ The dynamically alternating gradient regions around the micromagnets help anchor the particle clusters and propel them twice per rotation.^48^ This collective transport mechanism allows the transport speed to depend not on the particle size but on the rotational frequency and the periodicity of the magnetic field. As a result, the transport method significantly increases the separation speed at two jumps per rotation on the micromagnet chip. Similar acceleration collective behavior has been observed on magnetic garnet films, where hydrodynamic interactions promote collective transport on a structured micromagnet.^50^^,^^51^

At higher frequencies, the required fluid drag increases, and the magnetic clusters will eventually slow down, as shown in Figures 4B and 4D. However, in the case of using a stronger magnetic field (130 mT), the magnetic clusters stay synchronized at 1–6 Hz and achieve a higher maximum collective speed. The comparison between different operating frequencies can be directly observed in Videos S1, S2, and S3. The transition frequency, which is determined by the relative strength of the magnetic and fluidic interactions, can also be observed in other systems driven by rotating magnetic fields.^39^^,^^52^^,^^53^ We also observed this transition in the many-particle simulations (as shown in Figure 4B). In the simulation, we use a very simple model without any fitting that integrates the corresponding overdamped equations of motions of the magnetic particles (including their hydrodynamic interactions on a point particle level), and the results show excellent qualitative agreement with the experiments. More details of our numerical methods can be found in Note S8.

Video S1. Swarm transport at 18 mT from 1 to 6 Hz

Video S2. Swarm transport at 130 mT from 1 to 6 Hz

Video S3. Swarm transport at 130 mT from 7 to 20 Hz

### Characterization of collective transport

To obtain a deeper understanding of the collective transport under different driving conditions, we perform many-particle simulations of the collective transport of magnetic microparticles under different combinations of magnetic field strength and rotating frequencies. In detail, we randomly place 200 superparamagnetic microparticles at the start of four lanes of micromagnets, and the rotating magnetic field is applied (see more information in Video S4). Depending on the topic of study, we decide to stop the simulation after either the first 20 microparticles pass a predefined finish line to analyze the speed or a certain period of time (100 s) to analyze the particle configurations (e.g., width of the particle band).

Video S4. Particle simulation of collective transport

**Figure 5:**
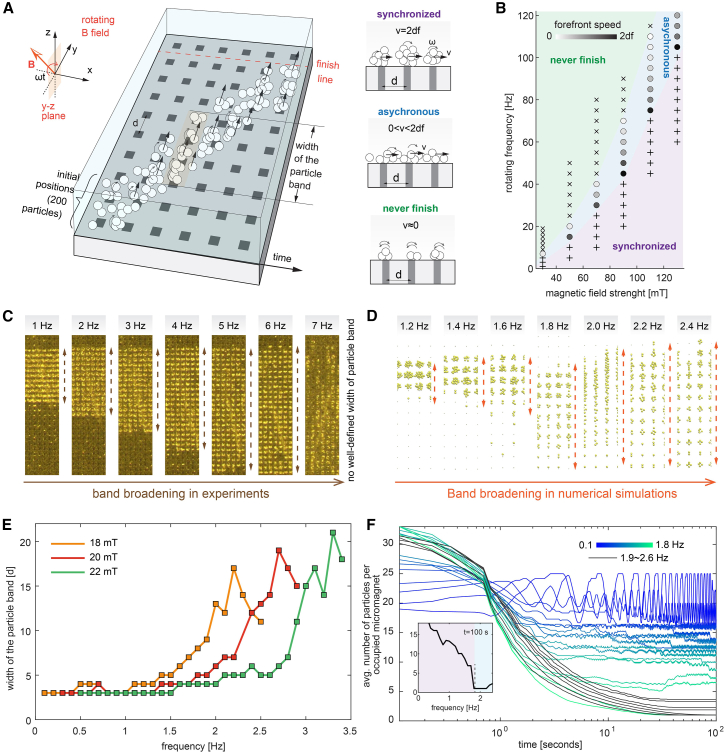
Characterization of the collective motion of swarm magnetic microparticles on a micromagnet chip (A and B) Simulation setup of the collective transport of magnetic microparticles. 200 particles are released in the starting region, and a rotating magnetic field of different magnitude and frequency is applied. Depending on the combination, three distinct “regimes” are observed in the simulation: synchronized, asynchronized, and never reaching the target. (C and D) The width of the particle band widens with increasing operating frequency. This behavior is observed in both simulations and experiments. (E) The width of traveling particle band of the same number of microparticles. This helps to determine the maximum traveling density of the particles on the chip. (F) Number of particles per occupied micromagnet. This can show the intrinsic probabilities of high-frequency swarm travel, and the speed can have a large variation.

We observe three different collective regimes of the magnetic particle swarms shown in Figures 4B and 5B: a synchronized regime (<1.9 Hz for B = 18 mT), an asynchronized regime (between 1.9 and 2.5 Hz, for B = 18 mT), and one that never reached the finish line (>2.5 Hz, B = 18 mT). We have already discussed the synchronized and asynchronized regimes in the previous section. In the never-finish regime, the particles are gradually dispersed among the micromagnets and only oscillate under the rotating field without moving forward. In this regime, which usually occurs at high frequency and low magnetic field, the magnetic force is much weaker compared to the hydrodynamic drag, resulting in zero net motion. Note that in the asynchronized regime, the velocity of the microparticle front distinctly decreases with increasing frequency for all tested magnetic flux densities. Using numerical simulations, we computed a diagram (Figure 5B) showing the transition for the different regimes at different magnetic fields.

An important parameter to quantify this collective transport is the width of the particle band, which refers to the flat, thin strip of space occupied by a finite number of microparticles during transport. In the synchronized regime, the particles in the simulations assemble into large clusters that occupy 3–4 rows of micromagnets and propel forward. During transport, the width of the particle band remains constant until the swarms reach the target. However, as the frequency increases, the width of the particle band starts to increase and widens due to the increasing hydrodynamic drag while moving along the lanes, even before reaching the asynchronous regime. We observed this behavior in both experiments and simulations (Figures 5C and 5D). The width increases rapidly in the asynchronous regime, where the forefront of the particle swarm is quickly propelled and many particles are left behind and even remain at micromagnets. This can be captured by the sharp increase in the width of particle band shown in Figure 5E. The corresponding data are captured 15 s after the start of the simulations, and we define the edges of the particle distribution to obtain the width of the particle band as the last row of micromagnets, where ≥5 microparticles are located.

Note that the increasing width of the particle band results in the stop of the transport in the asynchronized regime. As the number of particles is constant in the simulations, an increase in the width of the particle band will decrease the number of particles that occupy the particular micromagnets at a specific time. If this number is too low, then the hopping of the microparticles from one micromagnet to the next micromagnet becomes impossible, and the particles stay trapped at their current positions. This also means that the asynchronized regime and the never-finish regime are related: in the never-finish regime, the critical number of particles per micromagnet for which transport is still possible is reached before the finish line, and the front velocity cannot be calculated. Consequently, the concrete transition point between the asynchronized regime and the never-finish regime depends on the chosen finish line. In our simulations, we use the fourth row of micromagnets as the starting line and the tenth row of micromagnets as the finishing line.

We also plot the average number of particles per occupied micromagnet in Figure 5F to show the same dynamic behavior from a different perspective. Here, a micromagnet is considered occupied if at least one particle is located in its vicinity. In the synchronous regime, the number of particles per occupied micromagnet reaches a static and finite value. The magnetic microparticles are self-assembled into swarms and then “jump” between micromagnets while the magnetic field is rotating. Near the transition frequency and in the asynchronous regime, the width of the particle band increases rapidly, and thus the number of particles per occupied micromagnet decreases distinctly. At the transition frequency (1.9 Hz for B = 18 mT), we even observed that micromagnets are only occupied by a single microparticle. If this value is reached, then the transport stops, as we find that a single particle seems to be unable to hop from the micromagnet to the micromagnet. In the asynchronous regime and the never-finish regime, the number of particles per occupied micromagnet again reaches a finite value larger than one. Here, the critical number of particles per micromagnet for which transport is still possible increases, and consequently, the number of particles that are stuck at the particular micromagnet rises. The dependence of the final value of the number of particles per occupied micromagnet on the frequency is also depicted in the inset of Figure 5F. For the same amount of magnetic microparticles, the width of the particle band increases with rotating frequencies (easy to extract from the video). This also provides clues to the dynamics in the z direction, namely that at lower frequencies, the particle swarm can carry more particles in the z direction with a larger swarm.

### Collective transport across the flow

**Figure 6:**
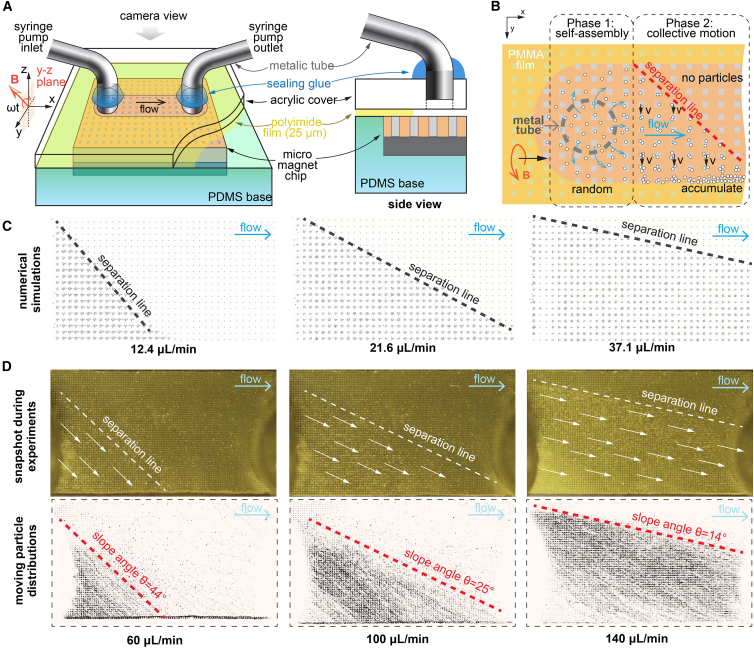
Locomotion of the magnetic microparticle swarm under perpendicular flow (A) Detailed view of the microfluidic setup. The setup consisted of a thin PMMA film (25 μm) to maintain a precise distance between the acrylic top cover and the micromagnet chip. The thickness of the film also determines the thickness in the z direction of the microfluidic channel. A camera captures the top view between the inlet and outlet. (B) An enlarged top view of the microfluidic setup. The swarm particles are driven in a positive y direction by the rotating magnetic field. The externally driven flow is perpendicular to the x direction. The combination of the two motions results in a clear separation line between the particle-free area and the particle-filled area. (C and D) Experimental and simulation results of the particle swarm motion under different flow velocities. The magnetic field rotates at 4 Hz.

In a microfluidic separation device, magnetic particles are designed to move perpendicular to the flow and accumulate on one side of the channel. This separation is stabilized by the laminar flow, and the flow of high-concentration magnetic particles can be easily collected at the end of the separation channel. In this work, we only evaluate the transport across the flow on the nickel micromagnet chip inside the microfluidic channel to quantify the magnetic separation efficiency. As shown in Figure 6, we use a syringe pump to control the flow rate inside the fluidic channel and observe the motion of the magnetic microparticles. The collective motion of the magnetic microparticles gives a clear separation line based on the density of the magnetic microparticles. With an increasing flow rate, the separation line changes its slope to adapt to the flow rate, which can be the sum of the magnetic propulsion speed and the local flow rate (Video S5). This effect of the changing slope of the separation line is also reproduced by our numerical simulations, which provide a useful indicator of the matching of the rotational speed and the externally driven flow (Video S6). In the optimal scenario, the separation line should cover the diagonal of the micromagnetic chip so that all magnetic microparticles reach the bottom of the fluidic channel after passing through the separation device. The separation system then achieves maximum throughput while maintaining the highest recovery efficiency.

Video S5. Collective transport under the flow

Video S6. Particle simulation of collective transport under flow

**Figure 7:**
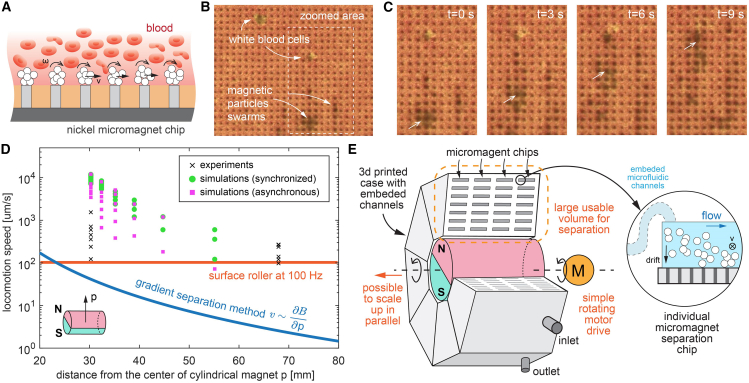
Swarming magnetic particles inside porcine blood and envisioned scalable system (A) Illustration of the swarm of magnetic particles moving in the blood. The magnetic particles accumulate at the bottom of the solution and near the micromagnet array. (B) Microscopic image of the experiments. We can identify some white blood cells and magnetic particle swarms. (C) Detailed view of the magnetic particle swarm inside the porcine blood. The particles are transported with a lower maximum velocity due to the increased viscosity and the non-Newtonian effect. For more information, see Video S7. (D) Comparison to different separation methods (gradient-based magnetic force and surface roller at 100 Hz) in terms of particle velocity at a distance from a given cylindrical magnet (same as in Figure 3A). The combination of micromagnets and rotating magnetic field shows superior separation performance over a wide working range. See the supplemental information for more details on the comparison. (E) An envisioned high-throughput magnetic particle separation device that integrates multiple micromagnet chips within a 3D-printed microfluidic housing.

We also perform a proof-of-concept experiment of magnetic particles in porcine blood. As shown in Figures 7A and 7B, the swarm of particles can collectively move in synchrony with the rotating magnetic field at low frequencies (Video S7). Blood is a much more complicated environment for magnetic microparticles, and many factors can influence the behavior and need further investigation. First, high concentrations of salt and proteins can affect the interactions between microparticles.^54^ Blood, as with many other fluids in the human body, is a viscoelastic fluid, and magnetic particle dynamics depend on operating frequencies. Cells may capture and engulf magnetic particles, as they are considered foreign by the immune system. In our experiments, we observed that some white blood cells became stuck on the micromagnetic chip (Figure 7B). However, this complex nature of swarm magnetic microparticles also provides opportunities in multiple applications, including blood purification,^16^^,^^18^ endovascular embolization,^55^ and the removal of biofilms from bacterial colonies.^56^

Video S7. Collective particle transport in porcine blood at 0.5 Hz

## Discussion

We have proposed a scalable separation method for magnetic microparticles using a combination of a micromagnet array and a rotating magnetic field. The self-assembled microparticle cluster can be effectively transported on the surface of the micromagnet chip, achieving very high speeds even under externally driven flow. To compare with other separation methods, we assume the same cylindrical magnet (grade: N35, diameter: 3 cm, length: 4 cm) and fitting magnetic microparticles to calculate the maximum speed based on different separation methods. The magnetic field and gradient at any distance from the center of the cylindrical magnet (z axis) are calculated using the finite element method. It is clear that the speed of the magnetic particles decreases as they move away from the magnet, since both the gradient and the field strength decrease. The proposed method in this paper is up to two orders of magnitude higher than the separation speed based on simply the magnetic field gradient. We also calculated the speed of the surface roller at 100 Hz as a reference, which is also much slower. The coupling between rotation and translation is approximately 0.2 body length per revolution, which is a value commonly found in the literature.^57^

However, speed is only one factor. As we observed in Figure 5, many other factors (e.g., width of the particle band) are also important for robust high-throughput particle separation. For example, band broadening shows that some particles cannot catch up with the front moving speed and are left behind. This means that for certain applications, we may want to avoid operating in the asynchronous regime and in the synchronous regime at high frequencies. Additionally, increasing the periodicity of the micromagnet array may appear to increase the transport speed. However, it also requires a much higher magnetic field to match the fluidic drag and a sufficient number of microparticles. Depending on the detailed application scenarios, different magnetic particles (e.g., SPIONs, NdFeB, iron, nickel, etc.) with different sizes (from nanometers to millimeters) and surface coatings (-COOH group or with antibodies) can be used in different liquid environments. Further investigation and optimization are required to understand the collective behavior and corresponding separation performance. Taking blood purification applications as an example, the high shear velocity near the chip surface may cause coagulation and generate thrombosis inside the fluidic channel, where surface coating may be important to prevent these problems.

As we discussed in the first section (microfluidic-centered vs. magnet-centered systems), the proposed method not only improves the separation speed of magnetic particles but also provides a larger working volume in which more devices and fluidic channels can be integrated around this given cylindrical magnet. In the future, many micromagnet chips can be assembled into a 3D-printed case with integrated parallel microfluidic connections, as shown in Figure 7E. Due to the inhomogeneity around the spinning NdFeB magnet, compromises must be made between the rotating frequency and the available workspace. And there is certainly room for further optimization of the integration strategy when hundreds of micromagnet chips are integrated into the 3D-printed microfluidic package and the fluidic network inside. Furthermore, we provide a roadmap and design guidelines in the supplemental information (Note S9; Figures S5–S9; Videos S8, S9, and S10) for developing scalable and high-throughput magnetic separation systems from a single microfluidic channel (∼0.1 mL/min) to benchtop machines (∼100 mL/min). This massive integration of microfluidic separation devices, utilizing only simple rotating magnets, has the potential to revolutionize current systems by creating compact, cost-effective, and highly efficient systems capable of achieving order-of-magnitude increases in throughput. This development holds significant promise for a wide range of applications, including blood purification, wastewater treatment, compact chemical reactors, DNA scavenging from large liquid samples, and many other scenarios involving the recovery, recycling, and reuse of magnetic micro- and nanorobots.

Video S8. Collective transport of iron oxide nanoparticle swarms

Video S9. Rolling motion of microparticle swarms on the 1D micromagnet array

Video S10. Particle simulation of collective transport under the external driven flow with different channel heights

## Experimental procedures

### Resource availability

#### Lead contact

Further information and requests for resources should be directed to the lead contact, Hongri Gu (hongri.gu@uni-konstanz.de).

#### Materials availability

This study did not generate new unique materials.

#### Data and code availability

All data needed to evaluate the conclusions in the paper are present in the paper and the supplemental information. The code that was used to perform the many-particle simulations and the CAD files are available via GitHub (https://github.com/AntonLueders/BDHM) and under the permanent DOI: https://doi.org/10.5281/zenodo.10982927.

## Supplemental information

Document S1. Notes S1–S10, Figures S1–S9, and Table S1

Document S2. Article plus supplemental information
