# Supplementary material for: Scalable high-throughput microfluidic separation of magnetic microparticles
Source: Device. 2024 Jul 19;2(7):100403. doi: 10.1016/j.device.2024.100403 (PMC11285115; doi:10.1016/j.device.2024.100403)
Supplement: Document S1. Notes S1–S10, Figures S1–S9, and Table S1 [file mmc1.pdf]

DEVICE, Volume *2*

Supplemental information

Scalable high-throughput microfluidic separation of magnetic microparticles

Hongri Gu, Yonglin Chen, Anton Lüders, Thibaud Bertrand, Emre Hanedan, Peter Nielaba, Clemens Bechinger, and Bradley J. Nelson

Note S1 Magnetic particles and properties

We use Micromer®-M (Micromod, 08-02-104, spherical with 10 µm size, surface: COOH, suspension in water, concentration: 50 $\mathrm{mg/mL}$, magnetization: 1.8 $\mathrm{A\,m^2/kg}$ at $H = 80\mathrm{kA/m}$, price: 264 euro for 10 mL) as the magnetic microparticle in the experiments. We then dilute the original particle suspension tenfold to become the input fluid that we use in the externally driven flow experiments depicted in Fig. 6 of the main article. Therefore, the particle concentration of the input fluid is 5 $\mathrm{mg/mL}$ (approx. 8.7×10^6 particles per mL). Due to the sedimentation, the particle concentration may slightly vary during the experiments.

Note S2 Design of the microfluidic device for magnetic particle separation

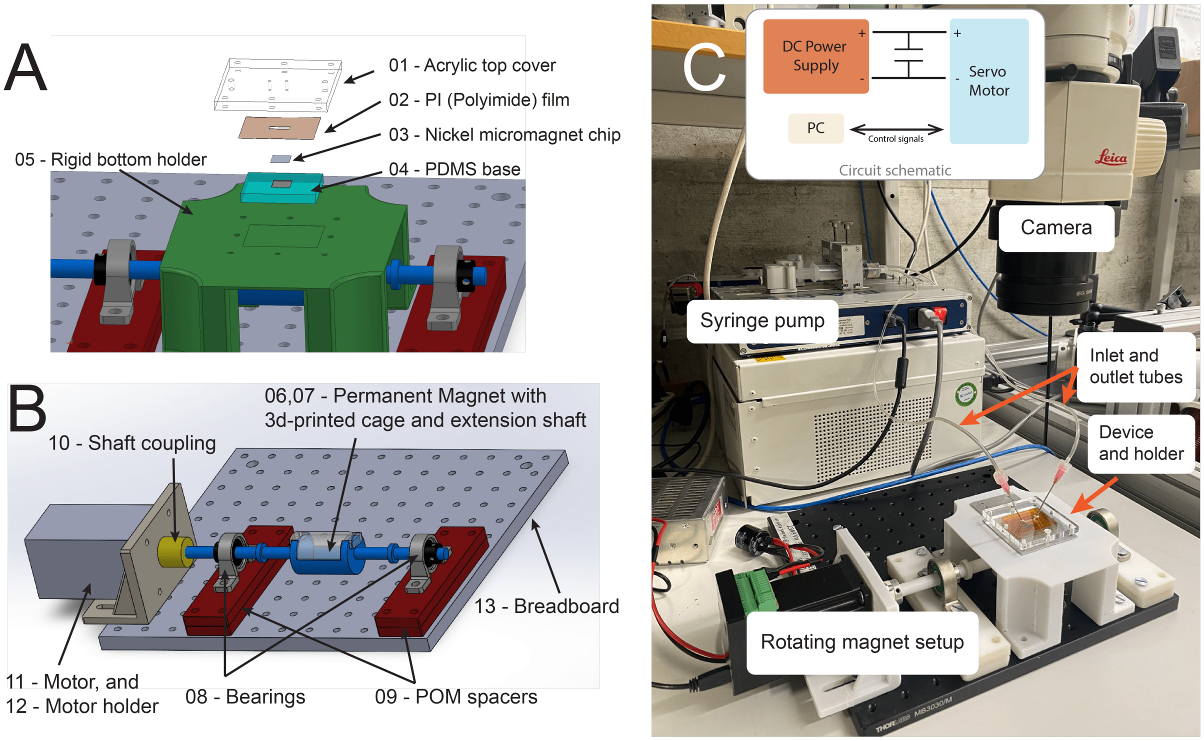

Figure S1. Design of the microfluidic particle separation device and the experimental setup. A.) The exploded view of the microfluidic separation device. The size of fluid channel (10 mm × 3 mm × 25.4 µm) is determined by the laser cut polyimide film. Eight M3 bolts and nuts are used to ensure a tight sealing between the acrylic top cover and the 3D-printed rigid bottom holder. B.) Assembly drawing of rotating magnet setup. We use a cylindrical NdFeB with radial magnetization (diameter = 30 mm and height = 40 mm). C.) Photograph of the complete experimental setup, with components labeled. The inlet and outlet tubes are connected to the syringe pump. The connection of the motor driver circuit is shown in the circuit schematic. The speed of the servo motor is controlled by a software (Plug & Drive Studio, Nanotec) on the PC.

We tested the magnetic particle separation in a microfluidic system shown in Fig. S1. The dimensions of the microfluidic channel are 10 mm × 3 mm × 25.4 µm (length × width × height). Fig. S1A demonstrates the assembly of the microfluidic separation device. It consists of 5 components (from top to bottom, as the numbers shown in Fig. S1A): 1) a 3-mm acrylic cover; 2) polyimide (Kapton) film (Thickness: 25.4 µm, McMaster-Carr, IL, USA); 3) nickel micromagnet chip; 4) PDMS base; 5) 3D-printed poly-carbonate rigid bottom holder, with holes for M6 screws on its legs for fixation on the breadboard. These layers are fixed with M3 bolts and nuts to ensure that the channel height is equal to the polyimide film. And soft PDMS layer ensures a tight fitting between the layers. We do not observe noticeable leakage even under a high flow rate at 3000 $\mu\mathrm{L/min}$.

The main objectives of the device design are to ensure height retention, anti-leakage, and good optical observability.

1) Height retention: The height of the fluid channel has a significant impact on the performance of particle separation. Therefore, ensuring a fixed and precise height control becomes an important consideration of device

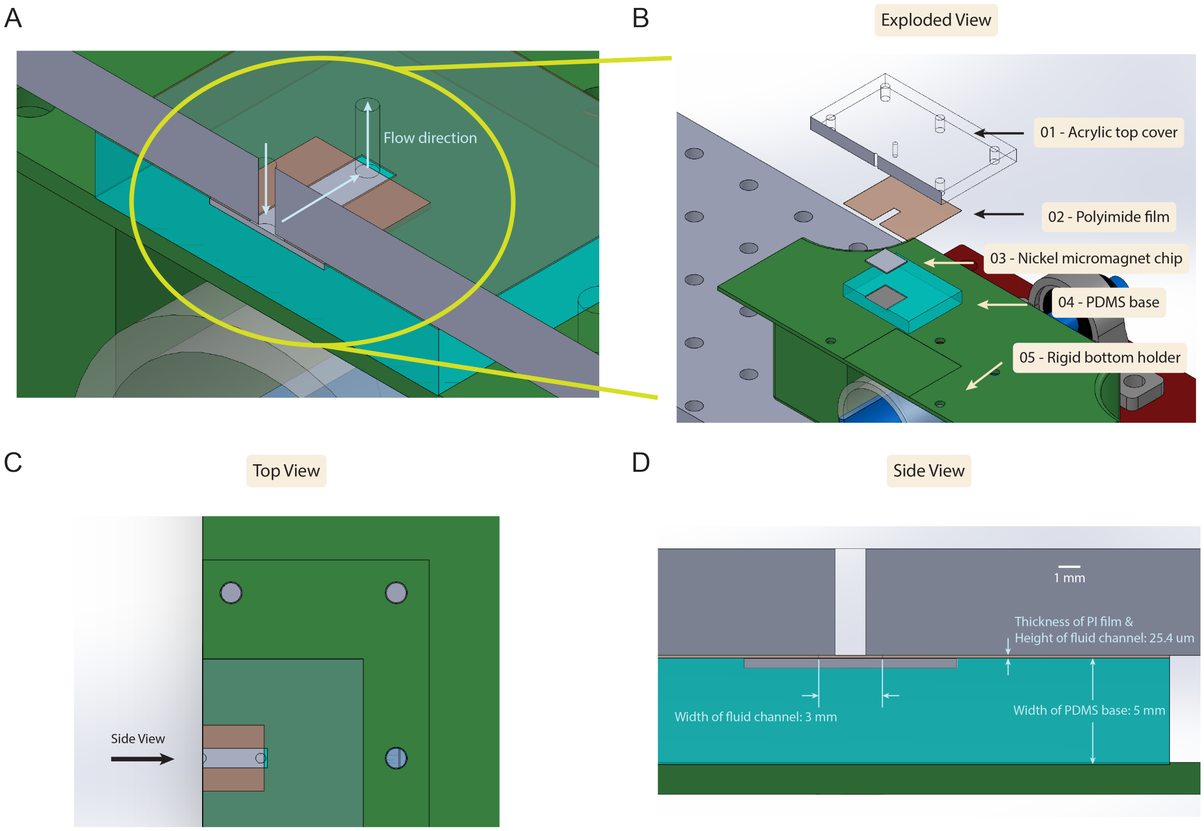

Figure S2. Details of fluid channel design. A.) 3D-sectional view of the fluid channel in Solidworks. The flow direction is marked by light blue arrows. The inlet and outlet directly sit on the fluid channel and guide the fluid through the channel, preventing leakage and contact with the PDMS base. B.) Exploded view of the fluid channel. The depth of the slot on the PDMS base is the same as the thickness of the chip. Hence, the upper surface of the chip and PDMS base is flat. The central rectangular slot in the PI film defines the dimensions of the fluid channel. C.) Top view of the sectional view of the microfluidic device. D.) Side view of the microfluidic device, reflecting the true scale of different parts in the device.

design. We use a polyimide (PI) film (Thickness: 25.4 μm, McMaster-Carr, IL, USA) to provide rigid support between the top acrylic cover and the soft PDMS base. The rectangular slit in the center of the film defines the dimensions of the fluid channel. (Fig. S2) Therefore, by tightening the bolts and nuts around the PDMS base and applying enough pressure on the Acrylic-PI film-PDMS interfaces, the height of the fluid channel is the same as the thickness of the PI film.

2) Anti-leakage: During the experiments, the device needs to be disassembled and cleaned once in a while, to get rid of the residual microparticles attached to the surfaces of the fluid channel. Therefore, the bonding between different parts of the microfluidic device cannot be permanent. Under the above constraints, preventing leakage and maintaining a fixed height of the fluid channel can be challenging.

Additionally, the inlet and outlet are located vertically above the fluid channel and guide the fluid through the channel without contacting the PDMS base. (Fig. S2A) Under sufficient pressure, the large contact area between the PI film and the PDMS base surrounding the fluid channel also helps prevent liquid from leaking out of the channel. Therefore, when sufficient pressure is applied by tightening the bolts and nuts, the interfaces between acrylic cover - polyimide film - PDMS are tight preventing leakage even at a high flow rate (typically 2000-3000 $\mu L/min$) without any adhesion agent.

3) Optical observability: The observation and recording of the dynamics of swarm microparticles are performed with a microscope placed above the setup. By covering a 3 mm-thick acrylic plate as the top cover, the fluid channel and magnetic particles are entirely visible from above. The details are shown in Fig. S1A.

4) Bubble-free: The height of the fluid channel (tens of microns) is several times smaller than its length and width. When the flow passes by, it is necessary to remove all bubbles inside the fluidic channel. The fluid inlet and

Table S1. Characteristic dimensions of components in the microfluidic separation device.

No #	Item	Characteristic dimensions
01	Acrylic top cover	Outline: 60 mm × 60 mm × 3 mm, holes for inlet/outlet: d1.5 mm
02	Polyimide film	Outline: 40 mm × 30 mm × 25.4 μm, Slot: 3 mm × 11 mm
03	Nickel micromagnet chip	Outline: 10 mm × 10 mm × 460 μm
04	PDMS base	Outline: 40 mm × 30 mm × 5 mm, Slot: same as the chip
05	3D-printed polycarbonate bottom holder	Thickness: 1.5 mm

outlet are placed directly above the chip so that the flow does not pass the gap between the PDMS and the chip. In practice, we control the flow rate and wet the channel first to make sure the system is bubble-free.

Note S3 Design of the rotating magnetic field setup

Fig. S1B shows the assembly of the rotating magnetic field setup. We use a cylindrical NdFeB magnet with radial magnetization (HKCM, 9962-72557, flux density at surface = 439 mT) inside a 3D-printed poly-carbonate cage with a shaft connected to a stepper motor (Nanotec, PD4-C5918L4204-E-01) with a shaft coupling (SUNGIL, SJC-25C-GR-6X10). The whole structure is supported by 2 bearing units (MISUMI pillow blocks, PBRSX10) to ensure a smooth rotation.

Circuit schematic of the servo motor system: As shown in Fig. S1C), the electrical connection of the motor is as follows: The DC power supply provides the power for the motor. An electrolyte capacitor is connected in parallel with the DC power supply. The motor has a built-in controller and driver. The control signals are sent from the PC to the motor via a USB connection.

Control of the motor speed: The manufacturer of the servo motor (Nanotec) provides software (Plug & Drive Studio) to directly set and control the speed of the motor in a GUI. No custom code is used to control the motor speed.

Note S4 Externally driven flow setup

Fig. S1C shows the structure of the experimental setup. A syringe pump (Cetoni Base 120) is used to control the flow rate. Two 18-gauge needles are inserted and glued on the acrylic cover of the microfluidic device as the fluid inlet and outlet. A microscope and camera (Basler acA2440-35uc) are mounted above to observe the particle swarm dynamics with and without the external flow.

In the externally driven flow experiment, we use a syringe to extract around 5 mL of stirred particle suspension and mount it on the syringe pump. An identical empty syringe is mounted on the other syringe pump to collect the liquid from the outlet. We wait 20 to 30 seconds for the system to reach equilibrium after changing the flow parameters. The nickel micromagnet chip is cleaned and re-used after each experiment.

Note S5 Magnetic flux density measurement

The magnetic field is measured using a customized setup as shown in Fig. S3A. A 3-axis magnetic field sensor (Metrolab, THM1176 Hall Magnetometer) is fixed on a POM plate mounted on a motorized XYZ stage. The cylindrical NdFeB magnet rotates at $f = 0.3$ Hz and the hall sensor records the waveform $B(t)$. The recorded flux density waveform is sinusoidal, as shown in Fig. S4. The sensor is adjusted to a specific vertical height and the average value of the waveform is calculated as the magnetic field strength at this height. Fig. S3B shows the curve of the averaged magnetic flux density B at different vertical distances r. In this curve, r is defined as the vertical distance between the surface of the magnet cage (not the magnet itself) and the bottom surface of the POM plate that carries the hall sensor. The POM plate is 4 mm thick. As the curve shows, the field strength decreases rapidly with an increasing r.

Note S6 Numerical simulations of the magnetic field around micromagnet array

The magnetic field around the nickel micromagnet chip is simulated using COMSOL Multiphysics. In the simulation, we assume that the permeability of the nickel micromagnets is 70 and the rest is 1. A simple stationary

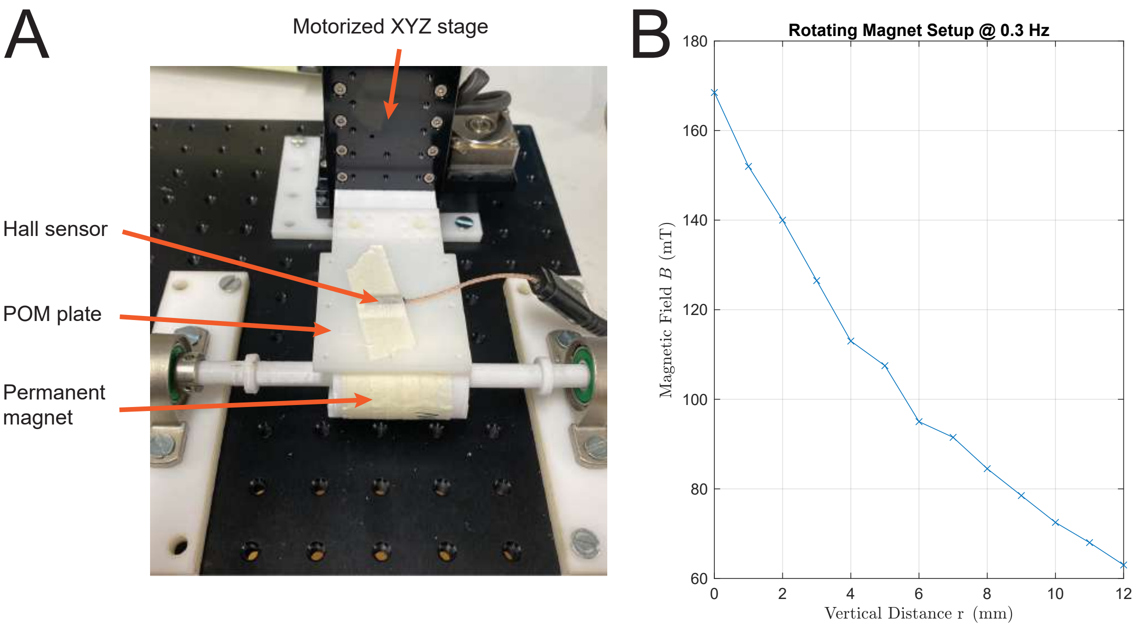

Figure S3. Magnetic field measurement near the rotating cylindrical magnet using a Hall sensor. A.) Photo of the setup for measuring the average flux density of the rotating permanent magnet. B.) Curve of multi-cycle averaged magnetic flux density versus vertical distance. The cylindrical magnet rotates along its axis at $f = 0.3$ Hz. The average magnetic flux density in the externally driven flow experiment is 130 mT.

case is implemented for magnetic fields in different directions using a simple model (Magnetic Fields, No Currents, mfnc). A uniform magnetic flux of 100 mT is applied to all outer surfaces of the geometries, and the results are visualized in Fig. 3D of the main article. In the visualization, we selected streamline positioning only on the surfaces of the micromagnets with adjusted streamline densities to provide an easier understanding of the 3D magnetic field distribution. The streamline density in Fig. 3D is only a qualitative representation of the local field strength.

Note S7 Comparison between different magnetic separation methods

We compare our collective transport of magnetic microparticles with two commonly used separation methods: (1) drifting based on the magnetic field gradient, and (2) surface roller under a rotating magnetic field. There are many factors in the experimental implementation of these systems that can affect the overall throughput of magnetic microparticle separation. In this work, we focus on a microscopic quantity, i.e., magnetic particle velocity, which eliminates the influence of the microfluidic channel dimensions. We believe that it more accurately reflects the differences in the performance of the separation methods.

The other important factor that makes comparison difficult is the different magnetic setups in the literature. For example, one can place a very powerful superconducting magnet near the microfluidic channel and achieve record throughput. However, this unique advantage cannot be easily replicated by other users considering implementation in other applications. Here, we assume that all methods can only use the identical cylindrical NdFeB magnet (grade: N35, diameter: 3 cm, length: 4 cm) and compare the corresponding moving speed of the magnetic microparticles.

We simulated the magnetic field around the cylindrical magnet in COMSOL Multiphysics (magnetic flux density $B_r = 1192$ mT) and used curve fitting (power law, as for a point dipole) to numerically calculate the corresponding magnetic field gradient. The gradient is used to calculate the moving speed of the magnetic microparticles utilized in this study, based on the reported size and magnetic properties (10 µm diameter, magnetization: 1.8 $\mathrm{A\,m^2/kg}$ at $H = 80\mathrm{kA/m}$). For the surface rollers, we assume that they are actively rotating at 100 Hz, which is usually considered a very high speed in the literature. We use the translation to rotation coupling factor $\alpha = 0.1$, which means that the magnetic particle moves 0.1 times its diameter under a single rotation, which is commonly reported in the literature for roller on a smooth surface.

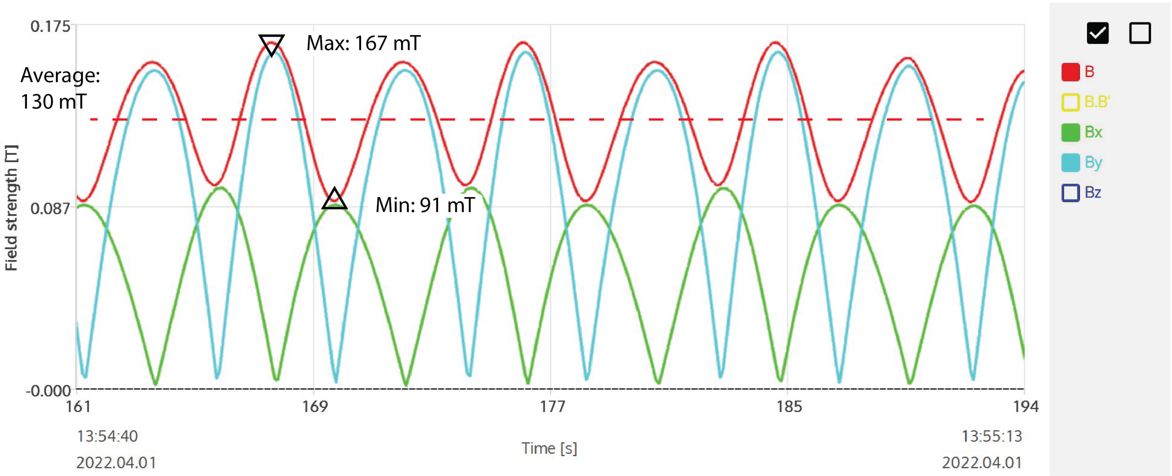

Figure S4. Recorded waveform of magnetic flux density near the rotating cylindrical magnet using a 3-axis hall sensor. The rotating frequency of the cylindrical magnet is 0.3 Hz, and the waveform of the total magnetic flux density B is sinusoidal. The average, maximum, and minimum values are 130, 167, and 91 mT, respectively. We use the average value to represent the magnetic field strength in experiments and numerical simulations (Note that the field strength is kept constant in the many-particle simulations).

Note S8 Many-particle Simulation

Various aspects that contribute to the dynamics of the systems (such as the hydrodynamic interactions between the particles and the walls, the magnetic field resulting from the micromagnet array, the additionally applied flow, and the external magnetic field with changing direction and magnitude) result in a high complexity of our setup. To break this complexity down and enhance the generality of our results, we implement a heavily simplified minimal toy model to corroborate our experimental findings.

In our simulations, we model the superparamagnetic microparticles as hydrodynamic point particles that possess a magnetic (point) dipole moment. To incorporate the finite volume of the real colloids (i.e., steric interactions) and avoid diverging dipole forces, we add purely repulsive pair interactions to the particle dynamics. However, hydrodynamic interactions that would result from these interactions are neglected to increase the stability of the simulations. This simplification leads to unphysical motions of “touching” particles, but the excellent qualitative agreement between our numerical model and the results of the experiments shows that our assumptions are sufficient to capture the collective phenomena sufficiently.

The micromagnets of the array are also modeled as magnetic point dipoles. To approximate their elongated shape, we assign the magnetic anisotropy of a prolate spheroid to the dipole moments by incorporating an (apparent) susceptibility tensor for magnetic ellipsoids in our corresponding calculations. We use a “wall” to separate the micromagnets and the superparamagnetic colloids, which is implemented as standard soft wall boundary conditions. Here, we use the same repulsive pair potential as for the repulsion between the microparticles[S1, S2]. The influence of the wall on the hydrodynamic interactions between the colloids is accounted for by applying the Blake tensor[S3] (for hydrodynamically interacting point particles in front of a wall) in our equations of motion. In our numerical model, we simplify the rotating external magnetic field by using a constant magnitude. Note that we assume that the magnetic moments of both, the particles and the micromagnets are linearly related to the external field for all applied field strengths, and no saturation magnetizations are reached.

For the simulations without an additional flow field, we utilize periodic boundary conditions in all directions that are not restricted by the soft wall separating the micromagnet array from the particles, where we always assume that the colloids only interact with their nearest periodic image. In these systems, the initial positions of the particles are randomly chosen at one “end” of the array. In the studies with an additional flow field, we add more walls to the systems: Firstly, we implement an additional soft wall parallel to the micromagnet array to set the constriction for the parabolic Poiseuille flow[S4] that we include in the equations of motion. Secondly, we include a soft wall at the end of the micromagnet array to which the particles are driven using the rotating magnetic field. The influence of both walls on the hydrodynamic interactions between the spheres is neglected. In the simulations with the additional flow field, we have to continuously add and remove new spheres to address the particle transport. For this, we use

a simple model where we delete particles that would leave the micromagnet array and spawn new particles at a constant rate along a predefined boundary.

Equations of motion: To describe the motion of the superparamagnetic particles, we integrate the Stokes law-like equations that connect the velocities of the colloids with the forces that act on them (through the grand mobility tensor)[S5]. This is in line with works such as Refs. S6, S7. Applying our additional approximations (for instance, neglecting the hydrodynamic interactions for the steric pair interactions of touching colloids), we find the integrator

$$\vec{r}_i(t+\Delta t) = \vec{r}_i(t) + \sum_j \hat{\mu}_{ij}(t)\vec{F}_j^{\mathrm{mag}}(t)\Delta t + \frac{D}{k_B T}\vec{F}_i^{\mathrm{ste}}(t)\Delta t + \vec{v}_i^{\mathrm{Poi}}(t)\Delta t \tag{S1}$$

for the position $\vec{r}_i$ of the ith sphere. Here, Δt is the length of the simulation step, $\hat{\mu}_{ij}$ is the hydrodynamic mobility tensor[S5] which depends on the positions of all particles and is based on the Blake tensor[S3] for point particles, $\vec{F}_j^{\mathrm{mag}}$ is the sum of the magnetic forces acting on particle j, $\vec{F}_i^{\mathrm{ste}}$ is the sum of the repulsive pair interactions acting on particle i (i.e., all particle-particle and particle-wall repulsions) and $\vec{v}_i^{\mathrm{Poi}}$ is the Poiseuille flow field[S4] that can be engaged. Note that the Poiseuille flow $\vec{v}_i^{\mathrm{Poi}}(t)$ depends on the position of the ith sphere at time t. The constants D and k_B are the diffusion coefficient of a sphere and the Boltzmann constant, respectively. The quantity T is the temperature.

Magnetic properties: After applying the external magnetic field, the superparamagnetic particles and micromagnets carry a magnetic dipole moment. In the real system, the direction and magnitude of these moments should be determined by the total magnetic field given by the external field and the dipole moments of the surrounding particles and micromagnets[S7]. In our simple toy model, however, we neglect the influence the colloids and micromagnets have on each other and assume that only the external magnetic field is relevant for the particular moments at a specific time. We further estimate that the orientation of the particles and the moments follow the orientation of the external rotating field instantaneously, and there are no phenomena such as hysteresis. Additionally, we assume that the external field and the magnetic moments of the components are linearly related for all applied field magnitudes (i.e., we do not reach saturation magnetizations).

At a specific time t, all superparamagnetic particles possess an identical magnetic moment $\vec{m}^{col}(t)$. In the same manner, all micromagnets of the array possess an identical magnetic moment $\vec{m}^{pol}(t)$ (but $\vec{m}^{col}(t) \neq \vec{m}^{pol}(t)$). Through simple algebraic transformations, we can express the magnetic moments of the micromagnets in multiples of the dipole moments of the particles. For this, we write

$$\vec{m}^{col}(t) = m_0 \vec{\vec{H}}(t) \tag{S2}$$

for the moment which each colloid holds, where m_0 is the corresponding magnitude and $\vec{\vec{H}}(t) = \vec{H}(t)/|\vec{H}(t)|$ is the direction of the external magnetic field $\vec{H}(t)$. Then, the magnetic moment of the micromagnets is

$$\vec{m}^{pol}(t) = \alpha^{\mathrm{pol}} m_0 \hat{\chi} \vec{\vec{H}}(t). \tag{S3}$$

Here, $\hat{\chi}$ is the diagonal (apparent) susceptibility tensor of an ellipsoid[S8] with the two unique eigenvalues

$$\chi^{\parallel} = \frac{\chi_0}{1+\chi_0 n^{\parallel}} \tag{S4}$$

$$\chi^{\perp} = \frac{\chi_0}{1+\chi_0 n^{\perp}} \tag{S5}$$

that correspond to the directions parallel and perpendicular to the orientation of the elongated micromagnets (which we model as ellipsoids). The corresponding quantity χ_0 is the susceptibility of the material, and we use the demagnetization factors

$$n^{\parallel} = \frac{1}{q^2-1}\left[\frac{q}{2\sqrt{q^2-1}}\ln\left(\frac{q+\sqrt{q^2-1}}{q-\sqrt{q^2-1}}\right) - 1\right] \tag{S6}$$

$$n^{\perp} = \frac{1-n^{\parallel}}{2} \tag{S7}$$

depending on the aspect ratio q of the micromagnets[S8] ($\chi^{\perp}$ is a double eigenvalue). The dimensionless coefficient α^{pol} is given by

$$\alpha^{\text{pol}} = \frac{V^{\text{pol}} H}{m_0}, \tag{S8}$$

where V^{pol} is the volume of a micromagnent.

The force resulting from the interaction between two magnetic moments $\vec{m}_i$ and $\vec{m}_j$ is computed as usually[S6,S7] using the relation for magnetic point dipoles

$$\vec{F}_{ij} = F_0^{\text{mag}} \left(\frac{\sigma}{|\vec{r}_{ij}|}\right)^4 \left\{ \left(\frac{\vec{\hat{m}}_i \cdot \vec{r}_{ij}}{|\vec{r}_{ij}|}\right) \vec{\hat{m}}_j + \left(\frac{\vec{\hat{m}}_j \cdot \vec{r}_{ij}}{|\vec{r}_{ij}|}\right) \vec{\hat{m}}_i - \left[\left(\frac{5\left(\vec{\hat{m}}_i \cdot \vec{r}_{ij}\right)\left(\vec{\hat{m}}_j \cdot \vec{r}_{ij}\right)}{|\vec{r}_{ij}|^2} \right) - \left(\vec{\hat{m}}_i \cdot \vec{\hat{m}}_j\right) \right] \frac{\vec{r}_{ij}}{|\vec{r}_{ij}|} \right\}, \tag{S9}$$

which contains the vector $\vec{r}_{ij}$ connecting the point dipoles, the diameter σ of the spheres and the dipole interaction strength

$$F_0^{\text{mag}} = \frac{3\mu_0 m_0^2}{4\pi\sigma^4}, \tag{S10}$$

where μ_0 is the vacuum permeability. Here, we used the abbreviations $\vec{\hat{m}}_i(t) = \vec{m}_i(t)/m_0$ and $\vec{\hat{m}}_j(t) = \vec{m}_j(t)/m_0$ marking the directions of the moments. Equation (S9) can be used for the magnetic interactions between two particles and the magnetic interactions between a particle and a micromagnet.

Especially in the simulations with the additional flow field, the numerical systems consist of a large number of particles and micromagenets. To save computational cost, we introduce cut-off radii describing distances after which we neglect the dipole interactions. For the colloid-micromagnet interactions, we use 3.5 times the lattice constant of the micromagnet array for said distance. For the dipole interactions between the particular microparticles, we use a cut-off radius of 2.5 times the lattice constant of the micromagnet array. Note that we apply these hard-coded cut-off radii independently of the dimensions of the simulation box or the micromagnet array and neglect interactions with more than the nearest periodic image in simulations with periodic boundary conditions (this can introduce artifacts that do not matter for our simplified toy model).

Repulsion: The purely repulsive particle interaction we use to model the finite size of the superparamagnetic microparticles is implemented via the Weeks-Chandler-Andersen (WCA) potential[S9]

$$V(|\vec{r}_{ij}|) = \begin{cases} 4\varepsilon \left[\left(\frac{\sigma}{|\vec{r}_{ij}|}\right)^{12} - \left(\frac{\sigma}{|\vec{r}_{ij}|}\right)^{6} \right] + \varepsilon, & |\vec{r}_{ij}| \leq 2^{1/6}\sigma \\ 0, & |\vec{r}_{ij}| > 2^{1/6}\sigma, \end{cases} \tag{S11}$$

where ε is the interaction strength. This pair potential is a shifted Lennard-Jones potential that is truncated at its minimum $r = 2^{1/6}\sigma$ to obtain only its repulsive part. The same potential is applied to realize the soft walls[S1,S2]. In fact, even the same interaction strength is inserted compared to the particle-particle interactions.

Poiseuille flow: For our studies with an additional flow (or velocity) field, we add the standard Poiseuille flow to the equations of motion[S4]. In detail, we set the soft wall over the micromagnet array at the position $z = 0$ and complete the Poiseuille flow setup with an additional soft wall with the position $z = h$. Thus, we can write

$$\vec{v}_i^{\text{Poi}}(t) = v_0 \left[1 - \frac{\left(z_i(t) - h/2\right)^2}{(h/2)^2} \right] \tag{S12}$$

for the flow velocity affecting the ith particle, where we assume that the two walls act as no-slip boundary conditions (for $\vec{v}_i^{\text{Poi}}$). Here, $z_i(t) = \vec{r}_i(t) \cdot (0,0,1)^{\text{t}}$ is the z-component of the position of sphere i and v_0 is the maximum velocity, which can be matched to generate similar volume flows as in the experiments.

Note that all quantitative flow rate data are always given concerning the setup of the experiments, which possess a width of $3\,\text{mm}$ and a height of approximately $30\,\mu\text{m}$ (which we rounded from the real $25.4\,\mu\text{m}$). This means that the average velocity is matched between the experiments and the simulations, and the flow rate numbers that are given in the text should be interpreted as the value the simulated system would have if it is set with the same dimensions as the real microfluidic channel.

Hydrodynamic interactions: Similar to works such as Ref. S6, we incorporate hydrodynamics on the Oseen level (i.e., we use a point particle model) in our numerical toy model. For this, we adapt the relations that can be

found in the theory part and the appendix of Ref. S3 to our computations. While we neglect the influence of all additional walls that are included during the studies with an additional flow field, we incorporate the effect of the soft walls between the particles and the micromagnet array (at position $z = 0$). Hence, we have to base the grand mobility tensor on the Blake tensor[S3]. In detail, for $i \neq j$, the mobility tensor is identical to said Blake tensor which means

$$\hat{\mu}_{ij} = \hat{G}(\vec{r}_i - \vec{r}_j) - \hat{G}(\vec{r}_i - \vec{r}_j') + \delta\hat{G}(\vec{r}_i, \vec{r}_j), \tag{S13}$$

where $\vec{r}_j' = \vec{r}_j - 2\vec{z}_j$ with $\vec{z}_j = \vec{r}_j(t) \cdot (0,0,1)^{\mathrm{t}}$ is the position of a mirror particle behind the wall, and

$$\hat{G}(\vec{r}) = \frac{1}{8\pi\eta|\vec{r}|}\left(\mathbb{1} + \frac{\vec{r}\otimes\vec{r}}{|\vec{r}|^2}\right) \tag{S14}$$

is the Oseen tensor, where η is the viscosity of the dispersion medium, $\mathbb{1}$ is the identity matrix, and $\otimes$ is the dyadic product[S3]. The matrix $\delta\hat{G}(\vec{r}_i, \vec{r}_j)$ is defined by the equations for its components

$$8\pi\eta\,\delta G_{xx} = -2z_i z_j\left[\frac{1}{s^3} - 3\frac{(x_i - x_j)^2}{s^5}\right] \tag{S15}$$

$$8\pi\eta\,\delta G_{yy} = -2z_i z_j\left[\frac{1}{s^3} - 3\frac{(y_i - y_j)^2}{s^5}\right] \tag{S16}$$

$$8\pi\eta\,\delta G_{zz} = 2z_i z_j\left[\frac{1}{s^3} - 3\frac{(z_i + z_j)^2}{s^5}\right] \tag{S17}$$

$$8\pi\eta\,\delta G_{xy} = 6\frac{z_i z_j\,(x_i - x_j)\,(y_i - y_j)}{s^5} = 8\pi\eta\,\delta G_{yx} \tag{S18}$$

$$8\pi\eta\,\delta G_{xz} = 2(x_i - x_j)\left[\frac{z_j}{s^3} - 3\frac{z_i z_j\,(z_i + z_j)}{s^5}\right] \tag{S19}$$

$$8\pi\eta\,\delta G_{zx} = 2(x_i - x_j)\left[\frac{z_j}{s^3} + 3\frac{z_i z_j\,(z_i + z_j)}{s^5}\right] \tag{S20}$$

$$8\pi\eta\,\delta G_{yz} = 2(y_i - y_j)\left[\frac{z_j}{s^3} - 3\frac{z_i z_j\,(z_i + z_j)}{s^5}\right] \tag{S21}$$

$$8\pi\eta\,\delta G_{zy} = 2(y_i - y_j)\left[\frac{z_j}{s^3} + 3\frac{z_i z_j\,(z_i + z_j)}{s^5}\right] \tag{S22}$$

with $\vec{r}_i = (x_i, y_i, z_i)^{\mathrm{t}}$, $\vec{r}_j = (x_j, y_j, z_j)^{\mathrm{t}}$ and $s = |\vec{r}_i - \vec{r}_j'|$, which we took out of the appendix of Ref. S3. Note that these relations only hold for a wall at position $z = 0$ and particles with $z_i > 0$.

We complete the grand mobility tensor by defining the remaining components for $i = j$ by

$$\hat{\mu}_{ii} = \frac{D}{k_B T}\mathbb{1} - \hat{G}(\vec{r}_i - \vec{r}_i') + \delta\hat{G}(\vec{r}_i, \vec{r}_i). \tag{S23}$$

The calculation of the hydrodynamic mobility tensor is the bottleneck of the simulations as it approximately scales with N^2, where N is the number of particles. Furthermore, the hydrodynamic interactions are long-ranged and, thus, we cannot introduce a cutoff radius or use similar techniques.

Remarks on the position of the wall: As we use a simplified hydrodynamic point particle model, unphysical artifacts can occur if the microparticles are too close to the wall, which is placed over the micromagents. In detail, the mobility tensor $\hat{\mu}_{ii}$ can change its sign when the corresponding particle penetrates the potential modeling the steric particle-wall interactions too much. To avoid this, we shift the position of the "steric representation of the wall" slightly above the wall we use in the calculation of the Blake tensor. This means that we use a wall positioned at $z = 0$ for the calculation of the Blake tensor, and a wall at $z = \delta z$ for the repulsive particle-wall interactions. One can interpret this approach as a setup, where a small layer is applied on the wall, that repels the microparticles but is perfectly permeable for the surrounding fluid. With this strategy, we avoid the artifacts in the simulations. However, note that tests showed that using $\delta z = 0$ does not affect the general collective dynamics of the system and the same qualitative behavior can be obtained.

General parameters used in the simulations: The simulations possess a large parameter set that we tried to match to the experimental setup as well as possible. Starting with the magnetic particles, we always assume a diameter of $10\,\mu\text{m}$, a density of $1100\,\text{kg/m}^3$ and a magnetization of $1.8\,\text{Am}^2/\text{kg}$ (at $H = 80\,\text{kA/m}$) (which corresponds to the properties of the real microparticles). With this, we obtain the magnitude of the magnetic moment $m_0 = 1.296 \times 10^{17}\,\text{m}^3\,|\vec{H}(t)|$.

We chose to match the volume and the aspect ratio of the elliptical micromagnets of the simulations to their experimental counterparts. Hence, we use $V^{\text{pol}} = 15 \times 15 \times 45\,\mu\text{m}^3$ and $q = 3.00$ and find $\alpha^{\text{pol}} = 781.31$. For the susceptibility of the micromagnets, we utilize $\chi_0 = 20$. This value can approximately be obtained from the relative magnetic permeability that was measured via a vibrating sample magnetometer and applied to FEM simulations in Ref. S10 for similar micromagnets. However, note that χ_0 does not influence the simulation distinctly as the apparent susceptibility tensor is dominated by geometric effects for large susceptibility[S8]. For instance, changing χ_0 from $\chi_0 = 20$ to $\chi_0 = 2000$ changes $\chi^{\parallel}$ and $\chi^{\perp}$ only by factors 1.45 and 1.11, respectively.

To maintain numerical stability, we set the interaction strength of the WCA potential to $\varepsilon = 10000 k_B T$. For the temperature, we apply 293.15K. If not stated otherwise, we utilize a distance of $60\,\mu\text{m}$ between the micromagnets. The point dipols that resemble the micromagnets are placed $21.0\,\mu\text{m}$ below the wall. This value was also optimized for numerical stability and is only possible as the point dipole micromagnets do not possess a "real" finite volume (the tips of the elliptical micromagnets would slightly poke through the wall). For the shift δz describing the difference between the position of the wall for the steric and the hydrodynamic interactions, we utilize $1.5\,\mu\text{m}$. In the simulations where an additional flow field is applied, we shift the upper wall also by $1.5\,\mu\text{m}$ to maintain the predefined distance between the walls (the Poiseuille flow is applied between $z = 0$ and $z = h\,\mu\text{m}$ nonetheless).

Specific parameters for the different runs: For the runs depicted in Fig. 4 of the main article, we utilize a micromagnet array of 4×100 micromagnets and randomly place 200 particles over the first two rows of said array. The length of the simulation step is set to $2.33 \times 10^{-5}\,\text{s}$ for $18\,\text{mT}$ and $2.33 \times 10^{-6}\,\text{s}$ for $130\,\text{mT}$. To calculate the velocity of the particle front, we define the fourth row of microparticles as a start line and the tenth row of microparticles as the finish line. If the first 20 particles pass the start line, we begin with the velocity calculations. After the first 20 particles pass the finish line, the simulation ends and the velocity can be obtained by the distance between the start and the finish line, as well as the time needed for the particles to travel this distance.

The simulations for the phase diagram in Fig. 5 are implemented analogously. Here, the length of the time step is also set to $2.33 \times 10^{-5}\,\text{s}$ or $2.33 \times 10^{-6}\,\text{s}$ depending on the stability of the simulations. To determine the width of the particle bands and the number of microparticles per occupied micromagnet, we also perform runs that do not end when the particle front reaches the finish line. In these runs, we set the total simulation time of approximately $115\,\text{s}$ and use a time step length of $2.33 \times 10^{-5}\,\text{s}$ throughout. The width of the particle band is defined as the distance between the last two rows of micromagnets where at least 5 microparticles are located (meaning that we compute the total number of all microparticles positioned at the four micromagnets corresponding to a row and take the distance between the first and the last row for which this number is higher or equal to 5). We consider a micromagnet occupied if it has at least a single particle in its vicinity. Formally, a particle is in the vicinity of the micromagnet when its position in the xy plane is in a square around the micromagnet with a box length of $60\,\mu\text{m}$.

For the simulations with a finite flow, we implement a micromagnet array of 60×20 micromagnets and utilize a time step length of $5.82 \times 10^{-6}\,\text{s}$. The height of the channel is chosen to be $h = 30\,\mu\text{m}$. Initially, 60 particles are randomly placed at one of the boundaries perpendicular to the flow field. Then, new particles spawn after a specific number of steps. At $12.4\,\mu\text{L/min}$, $21.6\,\mu\text{L/min}$, and $37.1\,\mu\text{L/min}$, a new particle appears after every 230, 132, and 76 simulation steps, respectively. Here, every simulation runs for 10^6 simulation steps each but we manually stop them after the separation line has fully formed. For supporting simulations analyzing the influence of the channel dimensions, we vary the height of the channel between $30\,\mu\text{m}$ and $120\,\mu\text{m}$. The spawn rate of the particles is adjusted for the increased volume.

Notes on gravity: Throughout all simulations, we neglect the influence of gravity on the particle dynamics. This can be justified by approximating estimates for the corresponding strengths of the magnetic and gravitational forces. For this, we assume a density of the colloids of $\rho = 1100\,\text{kg/m}^3$ and, additionally, take the buoyancy (which effectively reduces the influence of the gravity) into account. The resulting reduced force of gravity is roughly

$$F_G = (\rho - \rho_{\text{water}})V^{\text{col}} g \approx 1300\,k_B T/\sigma \approx 0.51\,\text{pN}, \tag{S24}$$

where ρ_{water} is the density of water, $V^{\text{col}} = 4/3\pi(\sigma/2)^3$ is the volume of the spherical colloids, and g is the acceleration corresponding to gravity. We can compare this value with the magnitude of the magnetic interaction force $\vec{F}_{ij}$ between an isolated colloid and an isolated micromagnet. Let us consider an example case with a low magnetic field with a strength of $18\,\text{mT}$ (which is the lowest field used in the numerical investigations). For simplicity, we further assume

that $\vec{m}^{col} \parallel \vec{m}^{pol}$, $\hat{\chi} \approx \mathbb{1}$, and $\vec{r}_{ij} \parallel \vec{m}^{col}$. With this, we find

$$|\vec{F}_{ij}| \approx 2F_0^{\mathrm{mag}} \alpha^{\mathrm{pol}} \left(\frac{\sigma}{|\vec{r}_{ij}|}\right)^4 \approx 4.0 \times 10^6 \, k_B T / \sigma \left(\frac{\sigma}{|\vec{r}_{ij}|}\right)^4 \approx 1.62 \, \mathrm{nN} \left(\frac{\sigma}{|\vec{r}_{ij}|}\right)^4 \tag{S25}$$

from Eq. (S9). Thus, it becomes clear that F_G is much smaller than $|\vec{F}_{ij}|$, and it is reasonable to neglect its influence at distances that are relevant to our simulations. For instance, $|\vec{F}_{ij}| \approx F_G$ at $|\vec{r}_{ij}| \approx 7.5\,\sigma = 75.0\,\mu\mathrm{m}$, which is more than twice the height of the channel used in the experiments. Subtracting the distance $75.0\,\mu\mathrm{m}$ of the micromagnets from the bottom wall (as they are placed behind the confining boundary), we obtain a height interval of $54.0\,\mu\mathrm{m}$ in respect to the bottom wall, where the influence of the gravity is smaller than the influence of the analyzed dipole interactions. Note that F_0^{mag} scales with $m_0^2 \sim H^2$ (see Eq. (S10)). This means that the difference between the orders of magnitude of the magnetic and gravitational forces becomes even much more distinct for larger magnetic field strengths.

Comments on the quantitative differences between the experiments and the simulations: Looking at Fig. 4B of the main article, the general results of the experiments and the many-particle simulations agree qualitatively: Up to a certain transition frequency, the collective dynamics are synchronous with the external magnetic field, meaning that the velocity of the particle front follows a linear relation. Passing the transition frequency, this linear relation between the velocity and the frequency is broken, and, finally, the particle front velocity decays.

Nevertheless, a clear quantitative deviation between the experimental and numerical results can be seen in Fig. 4B. Many aspects could be responsible for or could influence this deviation: To correctly assess the differences visible in Fig. 4B, one should keep in mind that the simulation results are based on simplified calculations with a model system consisting of 200 colloids. On the other hand, the experiments correspond to distinctly larger systems of much more microparticles. This should influence the results. Secondly, in Fig. 4D, it is visible that there is particle content at the micromagnets even before the particle band has reached them. As we discuss in Sec. “Characterization of collective transport” of the main article, we expect that a certain number of colloids must be assembled at a micromagnet so that jumps are still possible. Hence, the particle content that is already present on the micromagnets could influence the transition frequency because it could push the particle number of the “critical number of colloids” that is needed to keep the transport more synchronous and increase the observed particle front velocity. The last and most important aspect is that the presented many-particle simulations are a simple toy model that captures the essential collective dynamics of the complex experimental system (see above for the multiple simplifications that are made in the numerical calculations). Hence, the simulation results should strictly be interpreted on a qualitative level.

Remarks regarding the visualization: All images depicting the numerical result in the main article and all supplemental movies corresponding to the simulations are done using Visual molecular dynamics (VMD)[S11].

Source code availability: The code of the performed many-particle simulations is available via GitHub: `https://github.com/AntonLueders/BDHM`. This repository also contains the CAD data for the 3D-printed parts of the experimental setup. The corresponding permanent DOI is 10.5281/zenodo.10982927.

Note S9 How to design the micromagnetic array and choose system parameters?

In general, design parameters will strongly depend on the specific application, especially considering the complex collective dynamics of the magnetic microparticles. Here, we would like to provide a general guideline on how to design one’s own high-throughput systems based on our proposed scalable method. And share our insights on the gains and compromises in choosing certain parameters to maximize the final results balanced between throughput and reliability. The discussions are based on the simple system we presented in the paper (micrometer-sized magnetic particles and water).

Step 1: Choose magnetic microparticles: For most applications, there are limited choices for magnetic particles with the desired surface coating and size polydispersity. However, with advances in magnetic particle synthesis, it is possible to create your own magnetic microparticles with designer surface materials and functionalities.

Size selection: Magnetic particles need to be large enough to provide sufficient magnetic moment that scales with volume (L^3), however, one advantage of using magnetic particles is the high surface-to-volume ratio, and making the size L too large will compromise the total surface area ($\sim L^2$). Another issue related to size is Brownian motion, as thermal energy may become more pronounced at small sizes and the dynamics we observed in the paper may break down at the nanoscale.

Magnetic properties: Magnetic microparticles should have sufficient magnetic moments to interact strongly with the micromagnet array to move efficiently through the flow. We would say that the magnetic particles should be as permeable as possible. In addition, we would recommend that the magnetic moment of the microparticles have a linear dependence on the external magnetic field. The use of high coercivity magnetic materials will have its own magnetic memory, which will significantly increase the complexity of the particle dynamics (some aspects are discussed in supplemental Information Section S10 Magnetic properties of the micromagnet array and magnetic microparticles). This limits the choice of materials to superparamagnetic particles or soft magnetic materials.

Surface properties: The surface materials are usually determined by the intended applications (valuable catalyst, antigen for biomedical applications, etc.). However, dispersion and mixing with the media are also important. For water-based media, the surface of the microparticles must be hydrophilic so that they can be easily dispersed and react sufficiently in the media. If the surface is hydrophobic, the particle may want to cluster and compromise its advantages in a high surface-to-volume ratio and reaction time may increase in the first step before particle separation.

Cost: Since we are considering very high throughput applications (> 100 mL/min), the cost of the process can increase quickly and it is important to keep the price low or find a way to recycle and reuse the microparticles.

Further analysis and post processing: After the particles are separated from the bulk liquid, some applications require further treatment, and users need to consider whether the particles are compatible with these processes. For example, proteins or cells captured by the particles may need to be further analyzed to determine other properties. In another example, valuable catalysts may need to be recycled, cleaned, and reused.

Step 2: Micromagnet chip design: Our presented fabrication method based on 1-layer photolithography and electroplating supports a wide range of customized designs of micromagnet chips at reasonable cost.

Periodicity of micromagnet array: The periodicity of the micromagnet array should match the designed cluster size, which is a result of balancing transport speed and robustness. If one has a designed cluster size (100 microparticles), the periodicity of the micromagnet array should be about 1.5 to 2 times the size of the clusters.

We numerically study the influence on the periodicity by changing the periodicity of the micromagnetic array while keeping the rest of the parameters constant (B =18 mT @ 1Hz). As shown in Fig. S5, we can clearly identify there is an optimum for a given magnetic field strength and rotating frequency. However, the distribution of the swarm of microparticles also needs to be considered since it is a sign of transport robustness.

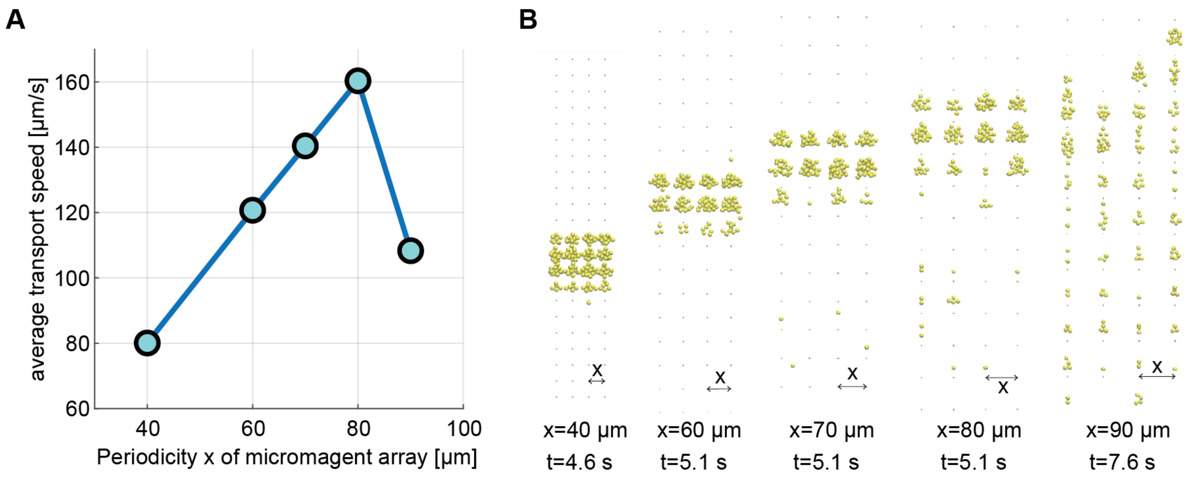

Figure S5. The influence of periodicity on the transport performance of swarm magnetic microparticles. A.) The average transport speed of magnetic microparticle swarms under the condition of an 18 mT rotating magnetic field at 1 Hz. B.) The morphologies of the swarm distributed on the micromagnetic array (top view) at the end of the numerical simulations.

Length and width of each micromagnet: Each micromagnet should have a long axis perpendicular to the chip substrate, and to prevent the magnetic coupling between the neighboring micromagnets, so the length and width of the micromagnet should be a small fraction of the periodicity L, a typical reference value should be $a, b < 0.3\ L$.

Height of the micromagnet: Increasing the height can ensure the magnetic anisotropy so that the long axis is always perpendicular to the chip. We recommend that the height is two times or more compared to the length and width (in our micromagnet array, the height is about 45 micrometers and the width is 15 micrometers). In

addition, increasing the height will increase the magnetic volume of the micromagnet, effectively increasing the magnetic attraction and promoting particle transport. However, increasing the height also increases the aspect ratio in microfabrication, which can be challenging and lowers the microfabrication yield rate.

Step 3: Design individual microfluidic channels: *Channel length and width:* First, the microfluidic channel should be long enough to allow all the magnetic microparticles to move across the flow and accumulate at one end of the microfluidic channel. This includes both the magnetic particle assembly process and the active swarming motion across the flow as shown in Figure 4A. In addition, the length and width should be adjusted to allow for the separation line across the channel, which is determined by the pumping speed of the externally driven flow and the collective moving speed of the magnetic microparticles.

Height of the channel: The height of the channel should have a characteristic length as the height of the micromagnet. The local gradient field, which is responsible for attracting free-floating microparticles in the liquid to the vicinity of the micromagnets, decays rapidly when moving away from the chip surface. This means that if the microfluidic channel is too high, particles at higher positions can be flushed away before they are attracted by the micromagnets and moved along with other particles. On the other hand, if the channel is too low, the flow resistance increases, and the strong shear flow profile can cause problems in the blood, as it can cause coagulation and thrombosis.

Step 4: Designing the rotating magnetic field setup and microfluidic networks: After determining the system parameters of the micromagnetic chip and microfluidic channels, it is time to consider how to scale up such a device structure in parallel to increase the throughput of the whole system. The design of the rotating magnetic field setup will provide us with sufficient working space that matches the required magnetic field strength. It is an optimization problem to provide the maximum workspace at a given cost for the NdFeB magnet. As shown in Fig. S7, our proposed design with a motor setup can be easily scaled in parallel with minimal interference between the rotating magnets.

The next step is to design the microfluidic networks. In the basic case of scaling in parallel, the microfluidic network can be embedded inside the 3D printed case, but the optimal connection architecture (parallel, hierarchical, etc.) is still unknown. In some cases, the suspensions may need to pass through three chips to achieve the best separation results. These issues are beyond the scope of this work and require further investigation in future work.

Step 5: Optimize operating frequency and flow rate: After the device is fully assembled, it is still possible to tune system parameters including the rotating frequencies, the pumping rate of particle suspensions, and the relative flow rate between the "clean" liquid channel and the recycled microparticle channel. Such parameters may require constant adjustment to match the dynamic situations in the separation, e.g., varying microparticle density.

In practice, one needs to decide the magnetic field strength threshold in the system (this determines the overall workspace) and the operating frequency of the rotating magnetic field. As shown in the Fig. S6. If one uses a low magnetic field threshold B1, one can in principle use a larger working volume, but this means the capped rotating frequency is low. On the other hand, if one chooses a higher magnetic field threshold, the corresponding usable working volume is smaller, but one can push up the rotating frequency to achieve faster particle speed. The particle rotating speed and the working volume together determine the throughput of the device. And we expect details may vary across different microparticles and applications.

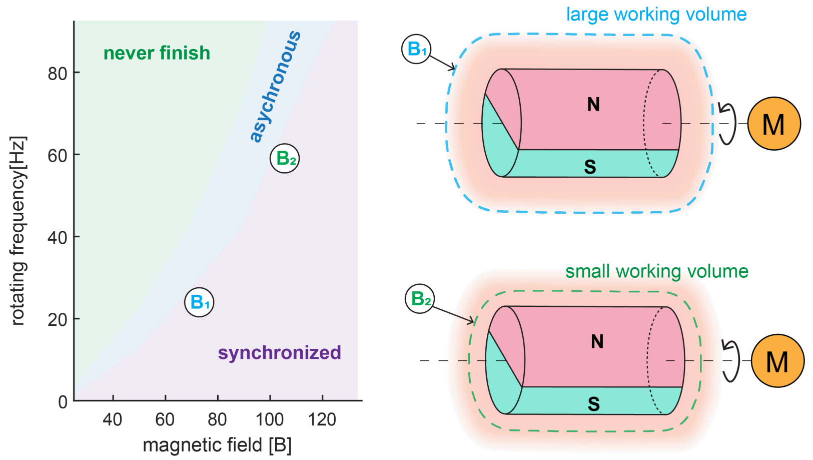

Figure S6. Compromises between magnetic field strength, workspace, and rotating frequency.

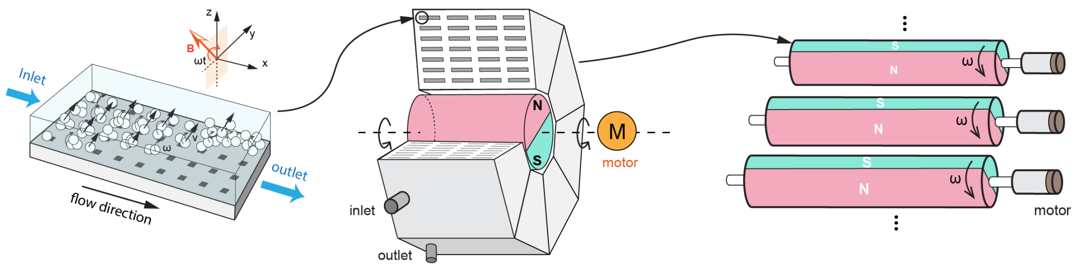

Design stage	Individual microfluidic device design (step 1-3)	Microfluidic architecture design (step 4)	Scalable system and operating parameter optimziaiton (step 5)
system parameters	magnetic particle<br>micromagnet chip design<br>rotating magnetic field	number of chips<br>fluidic network	operating frequency ω<br>pumping flow rate
dimension	10x3x1 mm<br>(single channel pack)	magnet: 3(D)x5(L) cm<br>case: 10(D)x5(L) cm	20x20x40 cm<br>(include 8 motors)
throughput	~0.1ml/min	~50 ml/min	~400 ml/min

Figure S7. Design process of scalable high-throughput magnetic particle separation system. It provides a map of key parameters at different design stages, and shows how the system can be further scaled to 100mL/min and more, even though it is not demonstrated experimentally in this work.

Note S10 Magnetic properties of the micromagnet array and magnetic microparticles

Magnetic properties of the micromagnets: Micromagnets must be dynamically magnetized by the rotating magnetic field. When the magnetic field is along the long axis of the micromagnets (perpendicular to the microchip), the micromagnets attract the surrounding particles (shown in red in Fig. 2 in Ref. S10), and when the magnetic field is along the short axis of the micromagnets (parallel to the microchip), the surrounding particles are repelled (shown in blue in Fig. 2 in Ref. S10). This alternating "attract and repel" behavior is critical for the microparticles to successfully move forward to the next micromagnet.

This feature determines that the micromagnets have a very small coercivity that the external rotating field can dynamically magnetize, demagnetize, and remagnetize the micromagnets. Soft magnetic materials (ferromagnetic materials with a small narrow hysteresis loop) are good choices. We choose electroplated multi-domain nickel in our fabrication methods because of its simplicity, low cost, and ease of scale-up. High permeability is also desirable as it can provide a higher local gradient field.

Another type of material we think is possible is superparamagnetic materials. They also have very low (almost zero) coercivity and high permeability. (e.g. iron oxide nanoparticles).

Magnetic properties of mobile microparticles: Unlike micromagnets, mobile particles can have a wide range of magnetic properties as long as they are strongly attracted to the magnetic field. This means that diamagnetic materials are not suitable. In this work, we mainly focus on the polymer-based particle with embedded iron oxide nanoparticles, which exhibit superparamagnetic behavior. This is due to the fact that the dynamics are easier to study because the particles have no memory for previously applied magnetic fields, and the magnetic coupling between neighboring magnetic particles is negligible because the nanoparticles are sufficiently separated.

We also have performed experiments with particles having different magnetic properties. For virgin NdFeB microparticles (average size: 5 micrometers), the particles are gradually magnetized and have their own magnetizations, which cannot be synchronized with the rotating magnetic field due to the high coercivity. As a result, the particles form larger and larger clusters (as shown in Fig. S8). This effect is not irreversible and the dynamics and behavior are difficult to predict.

We also performed some experiments with pure iron oxide nanoparticles (size: 30 nm). As shown in Fig. S9, the nanoparticles form very large clusters (like a cylinder) and tumble on the micromagnet chip. Due to the strong magnetic interaction between nanoparticles, the particles do not disassemble when the field is turned off. And the particles do not disperse well because of their nanometer size. So we think it may have limitations for certain applications.

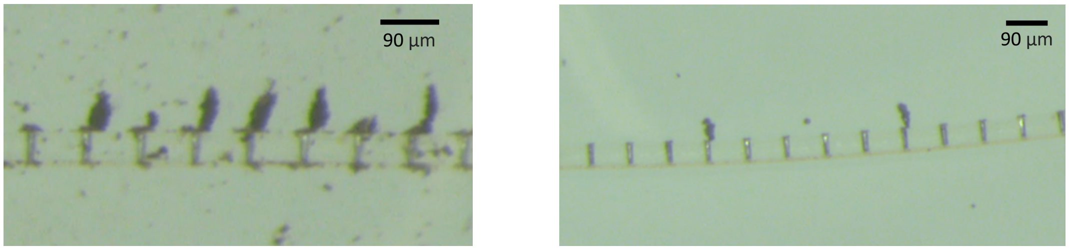

A B

Figure S8. Transport of ferromagnetic NdFeB microparticles on the 1-D micromagnet array. Two views (panel A and B) are presented depending on the local magnetic particle density.

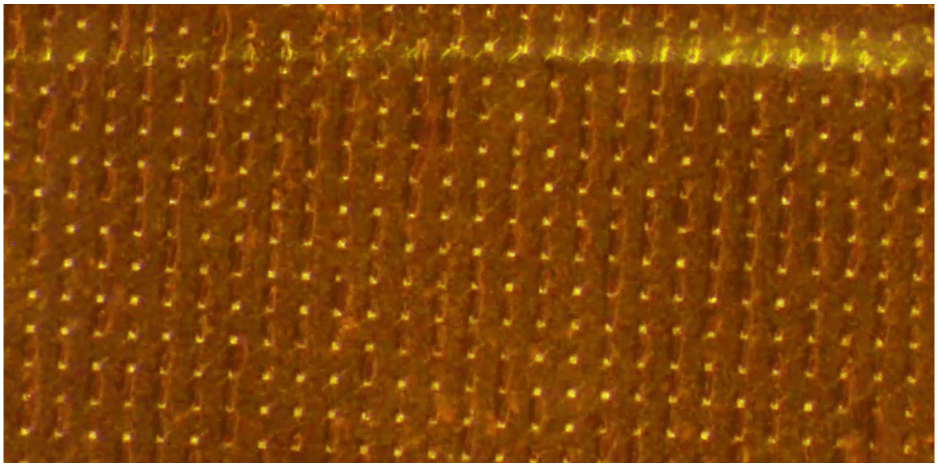

Figure S9. Iron oxide nanoparticles (average size: 30 nm) assemble into large clusters and are transported on the micromagnet array (periodicity: 120 micrometers). A rotating magnetic field of 40 mT is applied. The video is added as supplemental Movie S8.

References

[S1] Lüders, A., Siems, U. and Nielaba, P. (2019). Dynamic ordering of driven spherocylinders in a nonequilibrium suspension of small colloidal spheres. Phys. Rev. E . 99, 022601. 10.1103/PhysRevE.99.022601.

[S2] Stuckert, R., Lüders, A., Wittemann, A. and Nielaba, P. (2021). Phase behaviour in 2D assemblies of dumbbell-shaped colloids generated under geometrical confinement. Soft Matter 17, 6519–6535. 10.1039/D1SM00635E.

[S3] Jones, R. B. and Kutteh, R. (1999). Sedimentation of colloidal particles near a wall: Stokesian dynamics simulations. Phys. Chem. Chem. Phys. 1, 2131–2139. 10.1039/A809571J.

[S4] Bhattacharya, S., Bławzdziewicz, J. and Wajnryb, E. (2006). Hydrodynamic interactions of spherical particles in poiseuille flow between two parallel walls. Phys. Fluids 18, 053301. 10.1063/1.2195992.

[S5] Carrasco, B. and García de la Torre, J. (1999). Hydrodynamic properties of rigid particles: comparison of different modeling and computational procedures. Biophys. J. 76, 3044. 10.1016/S0006-3495(99)77457-6.

[S6] Granados Leyva, S., Stoop, R., Pagonabarraga, I. and Tierno, P. (2022). Hydrodynamic synchronization and clustering in ratcheting colloidal matter. Sci. advances 8, eabo4546. 10.1126/sciadv.abo4546.

[S7] Sing, C., Schmid, L., Schneider, M., Franke, T. and Alexander-Katz, A. (2010). Controlled surface-induced flows from the motion of self-assembled colloidal walkers. Proc. Natl. Acad. Sci. United States Am. 107, 535–40. 10.1073/pnas.0906489107.

[S8] Abbott, J. J., Ergeneman, O., Kummer, M. P., Hirt, A. M. and Nelson, B. J. (2007). Modeling magnetic torque and force for controlled manipulation of soft-magnetic bodies. IEEE Trans. Robot. 23, 1247–1252. 10.1109/TRO.2007.910775.

[S9] Weeks, J. D., Chandler, D. and Andersen, H. C. (1971). Role of repulsive forces in determining the equilibrium structure of simple liquids. J. Chem. Phys. 54, 5237–5247. 10.1063/1.1674820.

[S10] Gu, H., Hanedan, E., Boehler, Q., Huang, T.-Y., Mathijssen, A. J. and Nelson, B. J. (2022). Artificial microtubules for rapid and collective transport of magnetic microcargoes. Nat. Mach. Intell. 4, 678–684. 10.1038/s42256-022-00510-7.

[S11] Humphrey, W., Dalke, A. and Schulten, K. (1996). VMD: visual molecular dynamics. J. Mol. Graph. 14, 33–38. 10.1016/0263-7855(96)00018-5.
