# Supplementary material for: Scalable high-throughput microfluidic separation of magnetic microparticles
Source: Device. 2024 Jul 19;2(7):100403. doi: 10.1016/j.device.2024.100403 (PMC11285115; doi:10.1016/j.device.2024.100403)
Supplement: Document S2. Article plus supplemental information [file mmc12.pdf]

Device

Article

Scalable high-throughput microfluidic separation of magnetic microparticles

Graphical abstract

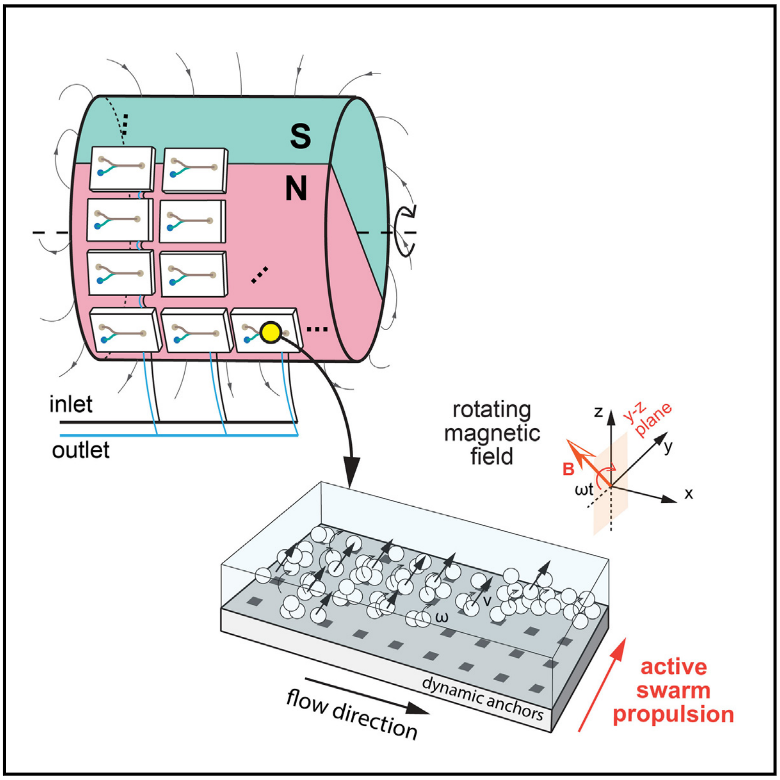

Highlights

- A scalable system to remove magnetic microparticles from liquid
- A conceptual shift from "microfluidic-centered" to "magnet-centered" system
- Micromagnets and a rotating magnetic field are used to boost collective movement
- Theoretical framework to compare the separation throughput using different methods

Authors

Hongri Gu, Yonglin Chen, Anton Lüders, ..., Peter Nielaba, Clemens Bechinger, Bradley J. Nelson

Correspondence

hongri.gu@uni-konstanz.de

In brief

This work presents a method for rapid and efficient separation of magnetic microparticles in liquids. With very high-throughput applications in mind, we focus on the scalability of the magnetic separation systems. We propose a conceptual evolution from a ""microfluidic-centric" to a "magnet-centric" system architecture and combine micromagnet arrays and rotating magnetic fields to increase separation efficiency. This work provides an alternate path toward high-throughput magnetic separation systems, which are critical for blood purification, micro- and nanorobot recycling, and wastewater remediation.

3 **Develop**
Prototype with demonstrated applications in relevant environment

Gu et al., 2024, Device 2, 100403
July 19, 2024 © 2024 The Authors. Published by Elsevier Inc.
https://doi.org/10.1016/j.device.2024.100403

CellPress

Device

CellPress
OPEN ACCESS

Article

Scalable high-throughput microfluidic separation of magnetic microparticles

Hongri Gu,[1,2,3,4,*] Yonglin Chen,[2,3] Anton Lüders,[1,3] Thibaud Bertrand,[2] Emre Hanedan,[2] Peter Nielaba,[1] Clemens Bechinger,[1] and Bradley J. Nelson[2]
[1]Department of Physics, University of Konstanz, Konstanz 78464, Germany
[2]Institute of Robotics and Intelligent Systems, ETH Zurich, Zurich CH-8092, Switzerland
[3]These authors contributed equally
[4]Lead contact
*Correspondence: hongri.gu@uni-konstanz.de
https://doi.org/10.1016/j.device.2024.100403

THE BIGGER PICTURE Reliable and effective removal of magnetic particles is a critical step in many emerging high-throughput applications. The required throughput for these applications can be orders of magnitude larger than today's microfluidic separation systems. Here, we propose a scalable and high-throughput microfluidic separation method. We discuss the underlying mechanism of collective transport of microparticles under flow, evaluate its unique advantages from a scalability perspective, and provide a roadmap for further optimization and parallel scaling to expand the capability of high-throughput removal of magnetic particles from liquid. The results pave the way for emerging applications of magnetic micro- and nanoparticles that require significantly higher throughput, including blood purification, water remediation, compact chemical reactors, and other scenarios of magnetic micro- and nanorobots that need to be recovered, recycled, and reused.

SUMMARY

Surface-engineered magnetic microparticles are used in chemical and biomedical engineering due to their ease of synthesis, high surface-to-volume ratio, selective binding, and magnetic separation. To separate them from fluid suspensions, existing methods rely on the magnetic force introduced by the local magnetic field gradient. However, this strategy has poor scalability because the magnetic field gradient decreases rapidly as one moves away from the magnets. Here, we present a scalable high-throughput magnetic separation strategy using a rotating permanent magnet and two-dimensional arrays of micromagnets. Under a dynamic magnetic field, nickel micromagnets allow the surrounding magnetic microparticles to self-assemble into large clusters and effectively propel themselves through the flow. The collective speed of the microparticle swarm reaches about two orders of magnitude higher than the gradient-based separation method over a wide range of operating frequencies and distances from a rotating magnet.

INTRODUCTION

Magnetic micro- and nanoparticles with sizes ranging from hundreds of nanometers to hundreds of micrometers hold promise across a wide array of applications, from environmental remediation[1] to biomedical engineering.[2] These particles have many unique properties that make them suitable for various applications, including high surface-to-volume ratios, high surface loading capacities, chemical stability, and biocompatibility. In recent years, they have become a popular platform technology where the surface can be coated with functional materials that expand their application scenarios, such as catalysts for accelerating specific chemical reactions,[3,4] ligands for selective binding of large biomolecules and cells,[5] and active sites for the adsorption of heavy metals in the environment.[6] In addition, magnetic particles can be driven to form active swarms to enhance collective functions such as virus detection,[7–10] and DNA scavenging.[11–13] In single-channel microfluidic devices, magnetic particles are typically injected from a storage channel, sufficiently mixed with the target fluid, and then magnetically separated and collected for further analysis. These microfluidic systems are widely used for immunoassay and the detection of specific molecules, with typical flow rates of a few milliliters per minute and sample volumes in the range of nanoliters to milliliters.[14,15]

Thanks to the developments in particle synthesis and functionalization, the available selection of magnetic microparticles has expanded, and the associated costs are continuously

This is an open access article under the CC BY license (http://creativecommons.org/licenses/by/4.0/).

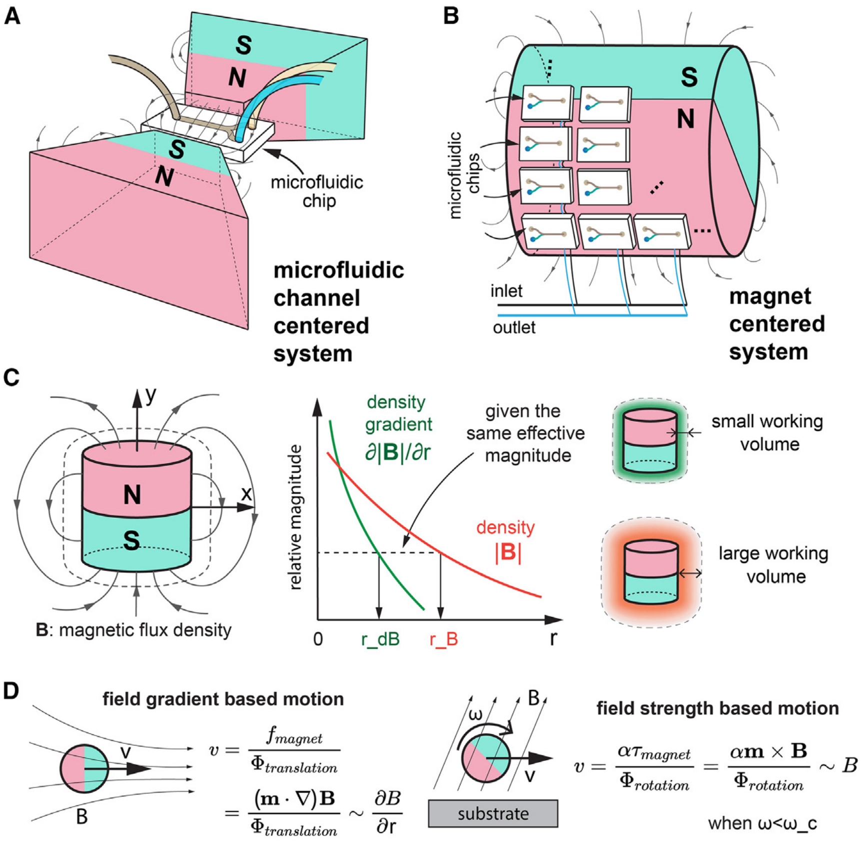

$$v = \frac{f_{magnet}}{\Phi_{translation}} = \frac{(\mathbf{m}\cdot\nabla)\mathbf{B}}{\Phi_{translation}} \sim \frac{\partial B}{\partial r}$$

$$v = \frac{\alpha\tau_{magnet}}{\Phi_{rotation}} = \frac{\alpha\mathbf{m}\times\mathbf{B}}{\Phi_{rotation}} \sim B$$

Figure 1. Concept and analysis of microfluidic-centered and magnet-centered systems for magnetic particle separation

(A and B) Illustration of a typical microfluidic-centered system and a magnet-centered system, respectively. The magnetic flux density maps around a typical cylindrical magnet.

(C) The field gradient dB/dx decreases faster than the magnetic flux density B away from the magnet. For the same relative magnitude, the gradient-based separation methods have a much smaller working volume than the field-strength-based separation methods. The working volumes for the two different methods are shown as colored shadows around the same magnet.

(D) Two examples of gradient-based motion (a magnet in a nonuniform magnetic field) and field-strength-based motion (a rolling sphere near a surface). f_{magnet} and τ_{magnet} are the force and torque applied on the mobile magnetic microparticle, and $\Phi_{translation}$ and $\Phi_{rotation}$ are translational and rotational drag coefficients, respectively. α is the coupling factor of the translation and rotation near a surface. See the supplemental information for more details on the comparison.

decreasing. This makes magnetic microparticles a viable option for many emerging applications that require much higher throughput (at least ~100 mL/min) compared to single-channel microfluidic devices. For example, in blood purification, magnetic microparticles can be used to selectively eliminate pathogens in acute blood infections (e.g., sepsis) where traditional antibiotic treatment is too slow.[16–18] With β-amyloid binders on the surface, magnetic microparticles can be used to remove toxic protein aggregates from the blood of patients with Alzheimer's disease.[19,20] In another example, researchers are exploring compact solutions with microfluidic devices for hospital wastewater treatment, where various pharmaceutically active compounds must be broken down before they enter the drainage system.[21,22] Magnetic micro- and nanoparticles inside a small system can be promising for such applications where industrial solutions are too large to fit.[1,22,23] These envisioned applications rely on robust separation methods to effectively and reliably recover magnetic microparticles at the end of the process, which is often the limiting factor for magnetic-particle-based applications.[24] In some cases, this can be critical, as unrecovered magnetic particles can cause damage to the human body and the environment (e.g., blocking distal blood vessels, degrading to microplastics, and being ingested by marine animals).

Most existing microfluidic separation methods rely on the magnetic force due to the local field gradient ($\vec{F} = (\vec{m}\cdot\nabla)\vec{B}$), where $\vec{m}$ is the dipole moment of the magnetic microparticle and $\vec{B}$ is the density flux of the magnetic field. In a simple example, one can place a bar magnet close to the microfluidic channel, and magnetic microparticles will drift and accumulate toward the north and south poles, where the magnetic field gradient is greatest.[17,25–29] This principle guides the design of most microfluidic magnetic microparticle separation devices, resulting in a microfluidic-centered design where the microfluidic channel is surrounded by strong and bulky magnets (Figure 1A). Such systems can achieve very good separation results with a typical throughput of a few milliliters per minute,[15,27,30–33] which is sufficient for most diagnostic-oriented applications (e.g., counting circulating tumor cells[15]). However, many emerging applications require significantly higher separation throughput, and such a system cannot be easily scaled to meet these needs. In the blood purification example, the ideal throughput is similar to that of a hemodialysis machine (approximately 200–500 mL/min), which is almost two orders of magnitude higher than the current state-of-the-art microfluidic separation systems.[34–36]

Increasing separation throughput by orders of magnitude is a challenging problem. Scaling up the dimensions of the microfluidic channel dramatically reduces the effective magnetic field gradient.[37–39] In addition, scaling out (i.e., increasing the number in parallel) of the same-size microfluidic devices requires a corresponding number of large magnets, where both the overall size and cost of the system become prohibitive. Many high-throughput systems have been reported in the literature, often with different microfluidic channel designs, different magnetic microparticles (ferromagnetic, paramagnetic, etc.), different magnet designs (electromagnet, permanent magnet), and different working fluids (viscous, viscoelastic, and even some biofluids).[4,31,40] To date, there is no general framework for the

CellPress
OPEN ACCESS

development of very high-throughput magnetic microfluidic systems in which all these reported systems can be fairly compared, analyzed, and evaluated.[41,42] It remains difficult for researchers to strategically combine the advantages of different existing separation systems and to develop new devices for new applications that may require orders of magnitude higher throughput.

In this article, we first analyze existing microfluidic separation methods from the perspective of throughput scalability and then introduce and discuss the experimental results of dynamic transport of swarming particles. We find that the inefficient use of the magnet is the main bottleneck for scalable magnetic separation. Based on this analysis, we constrain our design with a fixed magnet volume and try to use its surrounding magnetic field for separation as efficiently as possible. As a result, we obtain a magnet-centered separation system, which is different from the microfluidic-centered systems commonly found in the literature. In addition, we propose a novel separation method that combines the use of a micromagnet array and a rotating magnetic field. The high magnetic field gradient near the micromagnets attracts nearby magnetic microparticles, assembles them into large clusters, and helps them to "jump" on the micromagnet grid under a rotating magnetic field. This collective transport enables the rapid separation of magnetic particles in microfluidic channels, even in the presence of strong fluid flow. Combining experiments and numerical simulations, we studied the collective dynamics under different magnetic field strengths (18–130 mT) and rotation frequencies (1–130 Hz). We discover three distinct states of collective transport and quantify the key parameters (e.g., particle density and the width of the particle band) during the transition between the collective states. Finally, we show that this collective transport is an effective method for the separation of magnetic microparticles in water and in porcine blood, with the possibility of being adapted to various high-throughput applications.

Design strategy

Microfluidic-centered vs. magnet-centered systems

Microfluidic devices, together with external control modules, enable precise fluid manipulation (e.g., rapid mixing and reactions) in continuous flows or within droplets.[33] Using soft lithography and three-dimensional (3D) printing, microfluidic channels can be integrated and scaled in parallel to increase throughput while maintaining the benefits of channel size.[32] However, this is not the case for most magnetic separation systems, where bulky magnets are placed closely around the microfluidic channels to maximize the magnetic field gradient.[40,43] In some cases, multiple bulky magnets are implemented around the microfluidic channel in all directions. Increasing the throughput in these microfluidic systems also means increasing the number of accompanying bulky magnets. This will inevitably increase rapidly the size and cost of the scaled system, especially considering that the target throughput can be orders of magnitude higher than that of a standard microfluidic channel. In these scaled-up designs, the magnet, rather than the microfluidics, becomes the bottleneck for scaling because the use of magnets is inefficient.

To overcome this limitation of scalability and to further increase throughput, we propose the concept of "magnet-centered" systems, as shown in Figure 1B. In this concept, we assume that the available magnet has a fixed volume and that the microfluidic channels are arranged around this magnet. As a result, we can no longer add more magnets around the microfluidic channel to increase the local magnetic field gradient, as in the case of microfluidic-centered systems. This conceptual change forces us to think about how to use the magnetic field around this magnet as efficiently as possible and how to design microfluidic separation systems. For example, the gradient ($\partial B / \partial r$, where r corresponds to the distance from the magnet) decreases more steeply than the magnetic field B (in point dipole approximation: $\vec{B}(r) = \frac{\mu_0}{4\pi} \frac{3\widehat{\vec{r}}(\widehat{\vec{r}} \cdot \vec{m}) - \vec{m}}{|\vec{r}|^3}$, where $\vec{r}$ is a vector from the center of the magnetic dipole to the location of interest, $\vec{m}$ is the magnetic dipole moment, μ_0 is the vacuum permeability, and the hat means that a vector is normalized) in the space around a magnet. The magnetic field decreases at the rate of $|\vec{r}|^{-3}$, and the gradient decreases at the rate of $|\vec{r}|^{-4}$ when the magnet is considered a point dipole.[38]

This observation raises an important question about the available working volume around a given magnet. If we choose a minimum value for the magnetic field gradient and magnetic flux density that can be used for magnetic particle separation, we will find that the available working volume, where we can place microfluidic separation channels, is different for both cases. The working volume of the gradient-based method (marked green in Figure 1C) is much smaller than the working volume of the flux-based method (marked red in Figure 1C). Regardless of the actual separation efficiency, the available working volume alone makes a significant difference in terms of the throughput because more microfluidic channels and devices can fit into a larger working volume. In Figure 1D, we select two classical examples to move magnetic particles in a laminar flow. One is to use the magnetic force due to the magnetic field gradient, and the other is to use a magnetic torque under a rotating magnetic field. In this case, the magnetic-torque-based method, which depends on the magnetic flux density, is likely to have a larger available working volume than the gradient-based separation methods.

Combining micromagnets and dynamic magnetic fields

Among the gradient-based magnetic separation systems, one popular technique to further enhance the magnetic field gradient is to use micromagnets.[30] Patterned micromagnets can be deposited directly on the substrate as part of the microfluidic channel, placing them very close to the target magnetic microparticles. The strong gradient field around the micromagnets creates many magnetic field "hotspots" on the microfluidic chip. The effective magnetic force is so strong that it can be used to trap cells and large particle clusters.[44] In the bottom column of Figure 2, the magnetic flux lines are drawn to demonstrate such gradient fields and their effect on magnetic microparticles.

Another method that can increase the separation efficiency is to use a dynamic magnetic field, which is very popular in the microrobotics community. For example, a rotating magnetic field can apply a continuous torque to magnetic particles, propelling them forward as they rotate (e.g., surface roller and helix).[10,39,45] The speed of the moving microparticle depends on the rotational frequency and the field strength of the applied magnetic field. The magnetic particle is synchronized at low frequencies and

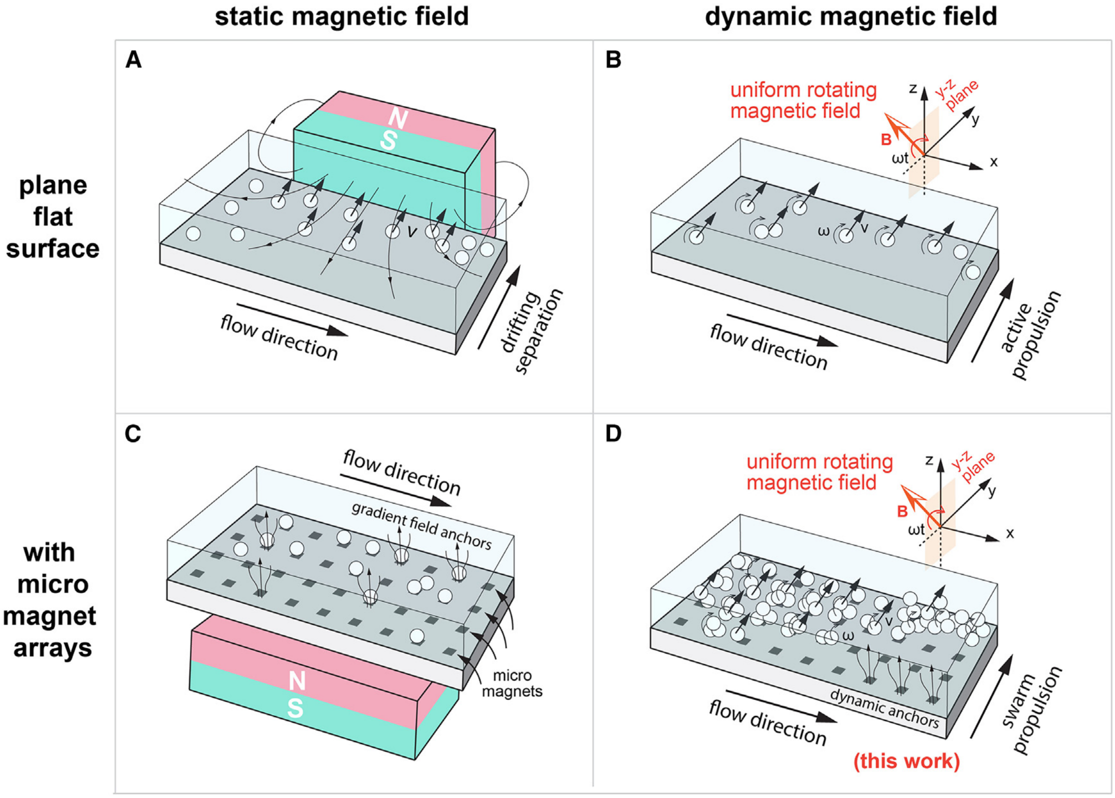

Figure 2. Four categories of magnetic particle separation devices using microfluidics

(A–D) The four categories are determined based on two independent criteria: (1) use of a static or a dynamic magnetic field or (2) use of an array of micromagnets or simply a flat surface in the microfluidic channel. In this work, we combined both a dynamic rotating magnetic field and micromagnets to achieve better dynamic locomotion of magnetic microparticle swarms through microfluidic flow.

slows down at high frequencies when the fluid drag is high. At a moderate magnetic field strength of 10 mT (which is much lower than the magnetic field strength for most separation devices), the particle can reach 10–100 body lengths per second in water,[46] which is typically higher than the speed we observed in gradient-field-based microfluidic separation devices. It is also known in the microrobotics community that for the same magnetic setup that can generate both a gradient field and a uniform rotating magnetic field, e.g., Octomag,[47] the rotating magnetic field can move particles much faster than the gradient-based method.[39] However, such systems can be costly and complicated compared to a single piece of magnet for magnetic particle separation purposes.

In Figure 2, we classify magnetic separation devices into four categories based on two independent criteria: (1) the use of a static magnetic field (Figures 2A and 2C) or a dynamic field (Figures 2B and 2D) and (2) the use of an array of micromagnets (Figures 2C and D) or simply a flat surface inside the microfluidic channel (Figures 2A and 2B). In this work, we combined both a dynamic magnetic field (i.e., a rotating magnetic field) and patterned micromagnets to achieve fast and collective transport of magnetic microparticles across the microfluidic flow. As shown in the 2D case,[48] the magnetic particle swarm can self-assemble into large clusters and move collectively, bridging the particles and achieving a much higher transport speed. The two methods work together in our experiments and show superior performance compared to other magnetic separation devices, which we explain later in the discussion.

Scalability and cost considerations

Implementing micromagnets and a rotating magnetic field can be expensive and may not be easily accessible to researchers in microfluidics and magnetic separation, as magnetic actuation systems are usually large and costly. These systems are usually composed of multiple electromagnets or permanent magnets that allow individual control to generate a calibrated magnetic field in the workspace.[47,49] This is critical for controlling the precise motion of the microrobot but is redundant for the purpose of magnetic particle separation. We use a simple magnetic setup that can be scaled to meet the throughput requirements in different applications. The setup is based on a permanent magnet rotating at a constant speed. Using a stepper motor and a motor controller, the speed of the rotating magnetic field can be easily controlled, which is less expensive than coil-based electromagnetic systems.

As depicted in Figure 3A, we use a cylindrical NdFeB magnet (grade: N35, diameter: 3 cm, length: 4 cm, surface magnetic flux density: 439 mT) with the dipole direction along the radial axis. The magnetic field strength measured at a distance from the surface of the magnet is shown in Figure S3. The NdFeB magnet is fixed inside a 3D-printed case, supported by two bearings, and connected to the motor by a coupling (the exact structure and

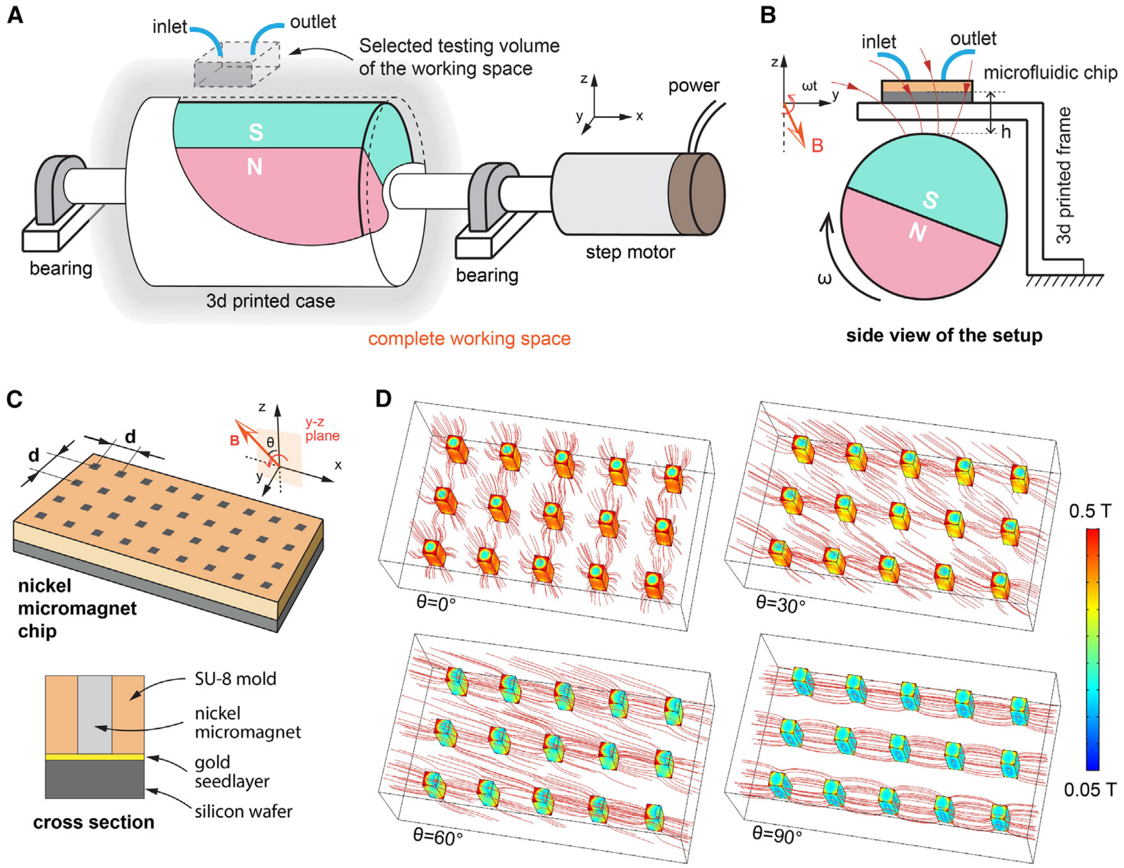

Figure 3. Experimental setup of a rotating magnetic field and the magnetic field in the vicinity of the micromagnet array

(A) An illustration of the experimental setup for scalable separation and the selected working volume of the available field, which can be further scaled.

(B) Side view of the setup showing the relative z-position between the rotating magnet, the 3D-printed frame, and the micromagnet chip in the microfluidic device.

(C) Structure of the nickel micromagnet chip. The periodicity d is equal to 60 μm in the x and y directions. The detailed fabrication process can be found in the supplemental information.

(D) Results of numerical simulations of the magnetic field around the micromagnet array. The orange lines indicate the local magnetic field directions and the relative density near the micromagnets. The colored area shows the magnetic flux density on the surface of the nickel micromagnets in the range of 50 mT to 0.5 T under an external uniform field of 200 mT. See the supplemental information for more details of the simulation.

computer-aided design [CAD] data of the actual setup can be found in Figures S1 and S2). The dynamic load on the motor is quite small, since the interaction with the magnetic particles can be negligible compared to the friction in the system. It is worth noting that the rotating magnetic field strength at any given position around this dipole magnet is not constant. Due to the symmetry of the dipole magnet in 3D, the magnetic field is stronger when facing the north and south poles and weaker in between, as shown in Figure S4. We neglect these differences and use the average field strength to represent the rotating magnetic field.

Inside the microfluidic channel, we fabricated the micromagnet array using nickel and SU-8 photoresist on a silicon chip. As shown in Figure 3C, the nickel micromagnets are embedded in the SU-8 layer with the same height (45 μm). The processing involves standard microfabrication with 1-step photolithography and nickel electroplating, both of which can be mass produced at a reasonable cost. Electroplated nickel exhibits typical soft magnetic properties with low coercivity and a linear response under an external magnetic field.[48] The micromagnet chip can be further improved with higher permeability magnetic materials (e.g., NiCo), optimized geometry, and better surface treatment to prevent unwanted particle adhesion and sorption. We used polystyrene-based microparticles (average diameter of 10 μm) with embedded superparamagnetic iron oxide nanoparticles (SPIONs). In this paper, we have only implemented a small microfluidic device using a fraction of the working volume of the rotating magnet to understand collective transport. One can design different setups to accommodate more microfluidic channels and chips to increase the overall throughput depending on the applications.

RESULTS

Collective transport on a nickel micromagnet chip

We observed that the magnetic microparticles interact with the nickel micromagnet chip in two distinct phases (Figure 4A). In the first phase, the magnetic particles are attracted to the surface of the micromagnet chip from different heights due to the

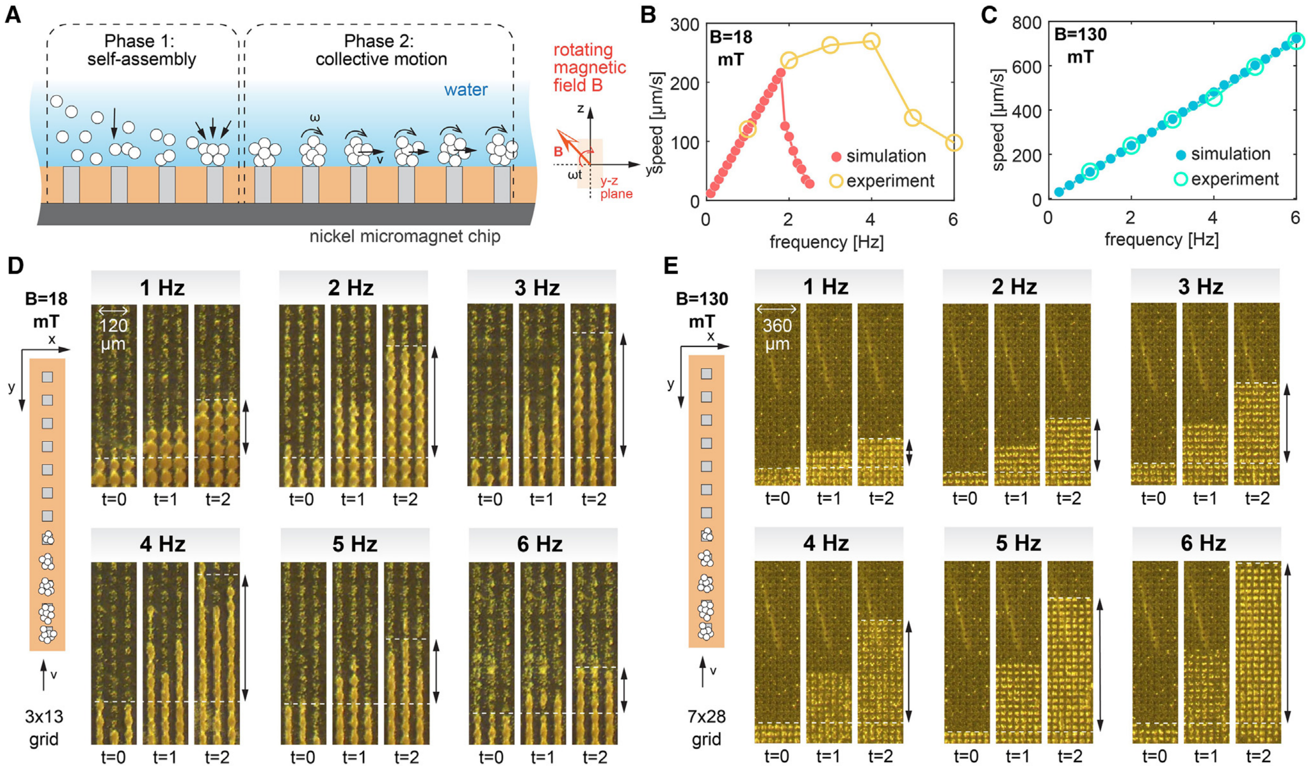

Figure 4. Locomotion speed of the magnetic particle swarm on the chip without externally driven flow

(A) Two distinct phases of magnetic microparticle motion: self-assembly through magnetic attractions and collective swarming motion.

(B and C) Measured velocity of the front line of the magnetic swarm.

(D and E) Time-lapse images of swarm particles moving under a uniform rotating magnetic field with different frequencies of the driving magnetic field. Time unit is seconds.

very high local magnetic field gradient near the micromagnets (as shown in Figure 3D). The time needed for the particles to reach the surface depends on their vertical positions. The gradient field around the micromagnets has a typical characteristic length of the micromagnet periodicity ($d = 60$ µm). When the magnetic microparticles are far away from the micromagnets, the gradient field is small and may never bring them close enough to the chip. This indicates that the design of the microfluidic channel requires an optimal channel height. If the channel is too high, the magnetic particles will not be in the magnetic field of the micromagnet chip and will simply flow away; and if the channel is too low, the flow rate will be too low with a very high flow resistance.

In the second phase, magnetic microparticles are on the surface of the magnetic microchip, and the behavior is dominated by the embedded micromagnet array. These microparticles assemble into large clusters due to the local magnetic field gradient and dynamically transfer from one micromagnet to a neighboring one, achieving collective transport. The local distortions of the magnetic field result in alternating gradient regions because the micromagnets are dynamically magnetized by the rotating magnetic field.[48] The dynamically alternating gradient regions around the micromagnets help anchor the particle clusters and propel them twice per rotation.[48] This collective transport mechanism allows the transport speed to depend not on the particle size but on the rotational frequency and the periodicity of the magnetic field. As a result, the transport method significantly increases the separation speed at two jumps per rotation on the micromagnet chip. Similar acceleration collective behavior has been observed on magnetic garnet films, where hydrodynamic interactions promote collective transport on a structured micromagnet.[50,51]

At higher frequencies, the required fluid drag increases, and the magnetic clusters will eventually slow down, as shown in Figures 4B and 4D. However, in the case of using a stronger magnetic field (130 mT), the magnetic clusters stay synchronized at 1–6 Hz and achieve a higher maximum collective speed. The comparison between different operating frequencies can be directly observed in Videos S1, S2, and S3. The transition frequency, which is determined by the relative strength of the magnetic and fluidic interactions, can also be observed in other systems driven by rotating magnetic fields.[39,52,53] We also observed this transition in the many-particle simulations (as shown in Figure 4B). In the simulation, we use a very simple model without any fitting that integrates the corresponding overdamped equations of motions of the magnetic particles (including their hydrodynamic interactions on a point particle level), and the results show excellent qualitative agreement with the experiments. More details of our numerical methods can be found in Note S8.

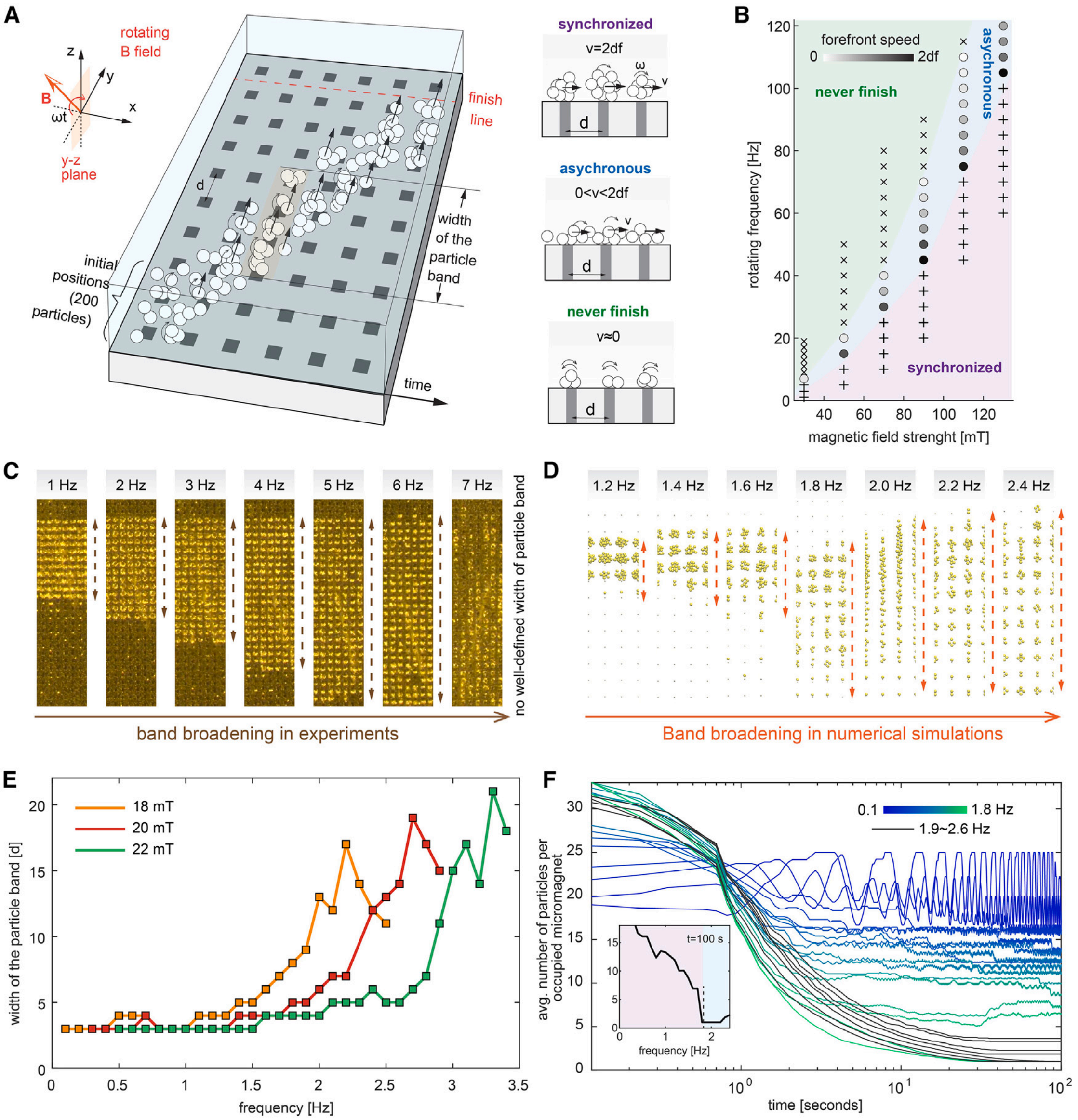

Figure 5. Characterization of the collective motion of swarm magnetic microparticles on a micromagnet chip

(A and B) Simulation setup of the collective transport of magnetic microparticles. 200 particles are released in the starting region, and a rotating magnetic field of different magnitude and frequency is applied. Depending on the combination, three distinct "regimes" are observed in the simulation: synchronized, asynchronized, and never reaching the target.

(C and D) The width of the particle band widens with increasing operating frequency. This behavior is observed in both simulations and experiments.

(E) The width of traveling particle band of the same number of microparticles. This helps to determine the maximum traveling density of the particles on the chip.

(F) Number of particles per occupied micromagnet. This can show the intrinsic probabilities of high-frequency swarm travel, and the speed can have a large variation.

Characterization of collective transport

To obtain a deeper understanding of the collective transport under different driving conditions, we perform many-particle simulations of the collective transport of magnetic microparticles under different combinations of magnetic field strength and rotating frequencies. In detail, we randomly place 200 superparamagnetic microparticles at the start of four lanes of micromagnets, and the rotating magnetic field is applied (see more information in Video S4). Depending on the topic of study, we decide to stop the simulation after either the first 20 microparticles pass a predefined finish line to analyze the speed or a certain period of time (100 s) to analyze the particle configurations (e.g., width of the particle band).

We observe three different collective regimes of the magnetic particle swarms shown in Figures 4B and 5B: a synchronized regime (<1.9 Hz for B = 18 mT), an asynchronized regime (between 1.9 and 2.5 Hz, for B = 18 mT), and one that never reached the finish line (>2.5 Hz, B = 18 mT). We have already discussed

the synchronized and asynchronized regimes in the previous section. In the never-finish regime, the particles are gradually dispersed among the micromagnets and only oscillate under the rotating field without moving forward. In this regime, which usually occurs at high frequency and low magnetic field, the magnetic force is much weaker compared to the hydrodynamic drag, resulting in zero net motion. Note that in the asynchronized regime, the velocity of the microparticle front distinctly decreases with increasing frequency for all tested magnetic flux densities. Using numerical simulations, we computed a diagram (Figure 5B) showing the transition for the different regimes at different magnetic fields.

An important parameter to quantify this collective transport is the width of the particle band, which refers to the flat, thin strip of space occupied by a finite number of microparticles during transport. In the synchronized regime, the particles in the simulations assemble into large clusters that occupy 3–4 rows of micromagnets and propel forward. During transport, the width of the particle band remains constant until the swarms reach the target. However, as the frequency increases, the width of the particle band starts to increase and widens due to the increasing hydrodynamic drag while moving along the lanes, even before reaching the asynchronous regime. We observed this behavior in both experiments and simulations (Figures 5C and 5D). The width increases rapidly in the asynchronous regime, where the forefront of the particle swarm is quickly propelled and many particles are left behind and even remain at micromagnets. This can be captured by the sharp increase in the width of particle band shown in Figure 5E. The corresponding data are captured 15 s after the start of the simulations, and we define the edges of the particle distribution to obtain the width of the particle band as the last row of micromagnets, where ≥ 5 microparticles are located.

Note that the increasing width of the particle band results in the stop of the transport in the asynchronized regime. As the number of particles is constant in the simulations, an increase in the width of the particle band will decrease the number of particles that occupy the particular micromagnets at a specific time. If this number is too low, then the hopping of the microparticles from one micromagnet to the next micromagnet becomes impossible, and the particles stay trapped at their current positions. This also means that the asynchronized regime and the never-finish regime are related: in the never-finish regime, the critical number of particles per micromagnet for which transport is still possible is reached before the finish line, and the front velocity cannot be calculated. Consequently, the concrete transition point between the asynchronized regime and the never-finish regime depends on the chosen finish line. In our simulations, we use the fourth row of micromagnets as the starting line and the tenth row of micromagnets as the finishing line.

We also plot the average number of particles per occupied micromagnet in Figure 5F to show the same dynamic behavior from a different perspective. Here, a micromagnet is considered occupied if at least one particle is located in its vicinity. In the synchronous regime, the number of particles per occupied micromagnet reaches a static and finite value. The magnetic microparticles are self-assembled into swarms and then “jump” between micromagnets while the magnetic field is rotating. Near the transition frequency and in the asynchronous regime, the width of the particle band increases rapidly, and thus the number of particles per occupied micromagnet decreases distinctly. At the transition frequency (1.9 Hz for B = 18 mT), we even observed that micromagnets are only occupied by a single microparticle. If this value is reached, then the transport stops, as we find that a single particle seems to be unable to hop from the micromagnet to the micromagnet. In the asynchronous regime and the never-finish regime, the number of particles per occupied micromagnet again reaches a finite value larger than one. Here, the critical number of particles per micromagnet for which transport is still possible increases, and consequently, the number of particles that are stuck at the particular micromagnet rises. The dependence of the final value of the number of particles per occupied micromagnet on the frequency is also depicted in the inset of Figure 5F. For the same amount of magnetic microparticles, the width of the particle band increases with rotating frequencies (easy to extract from the video). This also provides clues to the dynamics in the z direction, namely that at lower frequencies, the particle swarm can carry more particles in the z direction with a larger swarm.

Collective transport across the flow

In a microfluidic separation device, magnetic particles are designed to move perpendicular to the flow and accumulate on one side of the channel. This separation is stabilized by the laminar flow, and the flow of high-concentration magnetic particles can be easily collected at the end of the separation channel. In this work, we only evaluate the transport across the flow on the nickel micromagnet chip inside the microfluidic channel to quantify the magnetic separation efficiency. As shown in Figure 6, we use a syringe pump to control the flow rate inside the fluidic channel and observe the motion of the magnetic microparticles. The collective motion of the magnetic microparticles gives a clear separation line based on the density of the magnetic microparticles. With an increasing flow rate, the separation line changes its slope to adapt to the flow rate, which can be the sum of the magnetic propulsion speed and the local flow rate (Video S5). This effect of the changing slope of the separation line is also reproduced by our numerical simulations, which provide a useful indicator of the matching of the rotational speed and the externally driven flow (Video S6). In the optimal scenario, the separation line should cover the diagonal of the micromagnetic chip so that all magnetic microparticles reach the bottom of the fluidic channel after passing through the separation device. The separation system then achieves maximum throughput while maintaining the highest recovery efficiency.

We also perform a proof-of-concept experiment of magnetic particles in porcine blood. As shown in Figures 7A and 7B, the swarm of particles can collectively move in synchrony with the rotating magnetic field at low frequencies (Video S7). Blood is a much more complicated environment for magnetic microparticles, and many factors can influence the behavior and need further investigation. First, high concentrations of salt and proteins can affect the interactions between microparticles.[54] Blood, as with many other fluids in the human body, is a viscoelastic fluid, and magnetic particle dynamics depend on operating frequencies. Cells may capture and engulf magnetic particles, as they are considered foreign by the immune system. In our experiments, we observed that some white blood cells

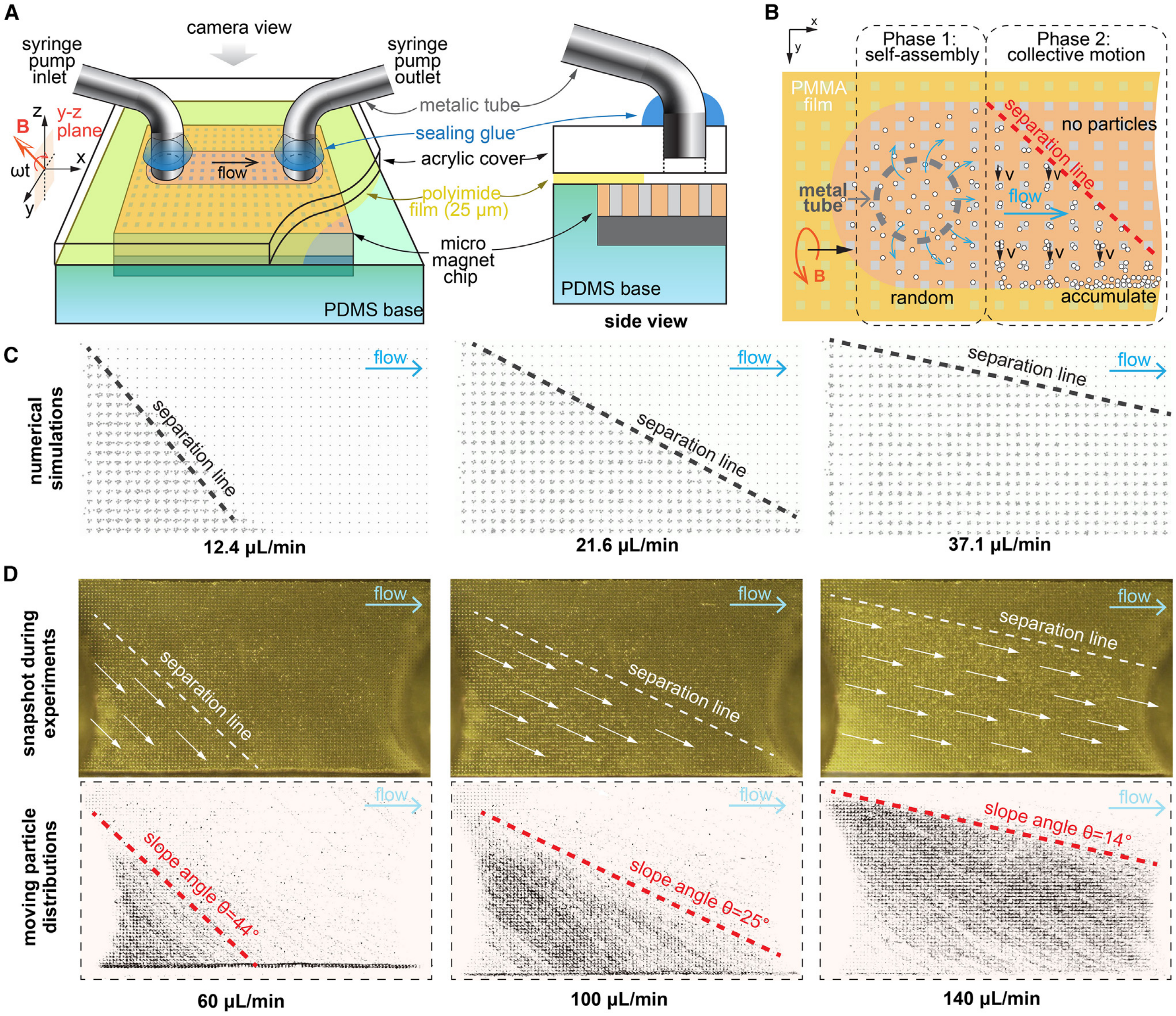

Figure 6. Locomotion of the magnetic microparticle swarm under perpendicular flow

(A) Detailed view of the microfluidic setup. The setup consisted of a thin PMMA film (25 μm) to maintain a precise distance between the acrylic top cover and the micromagnet chip. The thickness of the film also determines the thickness in the z direction of the microfluidic channel. A camera captures the top view between the inlet and outlet.

(B) An enlarged top view of the microfluidic setup. The swarm particles are driven in a positive y direction by the rotating magnetic field. The externally driven flow is perpendicular to the x direction. The combination of the two motions results in a clear separation line between the particle-free area and the particle-filled area.

(C and D) Experimental and simulation results of the particle swarm motion under different flow velocities. The magnetic field rotates at 4 Hz.

became stuck on the micromagnetic chip (Figure 7B). However, this complex nature of swarm magnetic microparticles also provides opportunities in multiple applications, including blood purification,[16,18] endovascular embolization,[55] and the removal of biofilms from bacterial colonies.[56]

DISCUSSION

We have proposed a scalable separation method for magnetic microparticles using a combination of a micromagnet array and a rotating magnetic field. The self-assembled microparticle cluster can be effectively transported on the surface of the micromagnet chip, achieving very high speeds even under externally driven flow. To compare with other separation methods, we assume the same cylindrical magnet (grade: N35, diameter: 3 cm, length: 4 cm) and fitting magnetic microparticles to calculate the maximum speed based on different separation methods. The magnetic field and gradient at any distance from the center of the cylindrical magnet (z axis) are calculated using the finite element method. It is clear that the speed of the magnetic particles decreases as they move away from the magnet, since both the gradient and the field strength decrease. The proposed

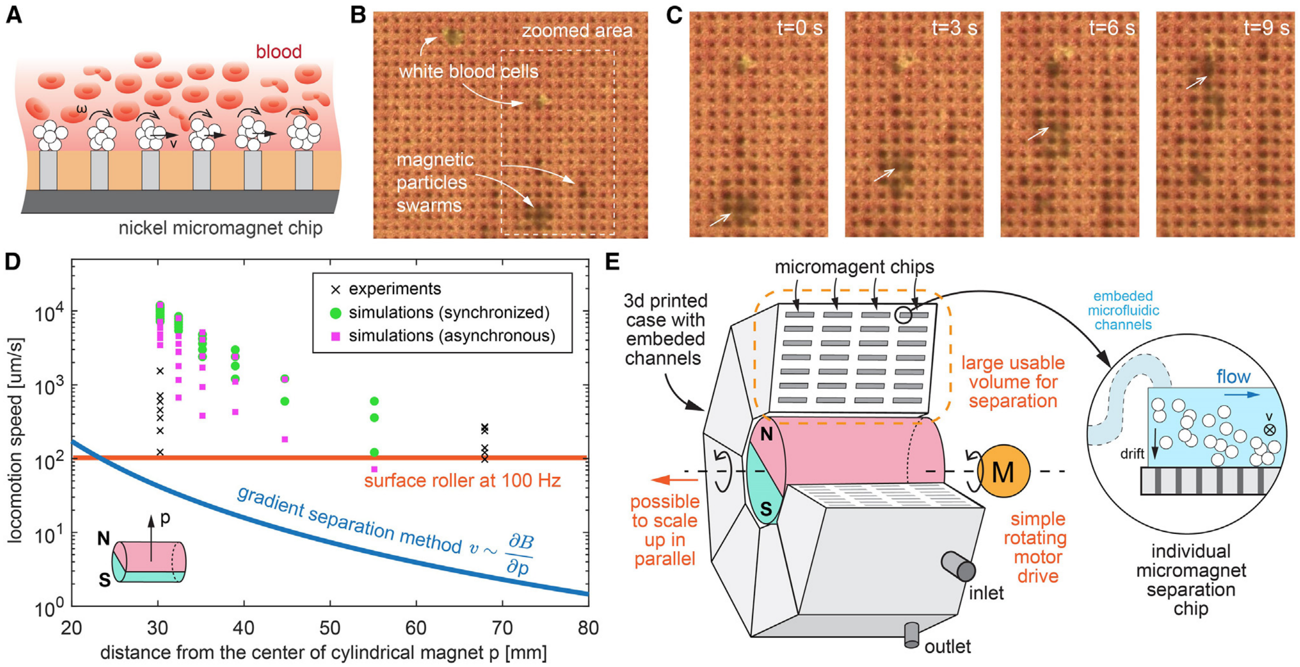

Figure 7. Swarming magnetic particles inside porcine blood and envisioned scalable system

(A) Illustration of the swarm of magnetic particles moving in the blood. The magnetic particles accumulate at the bottom of the solution and near the micromagnet array.

(B) Microscopic image of the experiments. We can identify some white blood cells and magnetic particle swarms.

(C) Detailed view of the magnetic particle swarm inside the porcine blood. The particles are transported with a lower maximum velocity due to the increased viscosity and the non-Newtonian effect. For more information, see Video S7.

(D) Comparison to different separation methods (gradient-based magnetic force and surface roller at 100 Hz) in terms of particle velocity at a distance from a given cylindrical magnet (same as in Figure 3A). The combination of micromagnets and rotating magnetic field shows superior separation performance over a wide working range. See the supplemental information for more details on the comparison.

(E) An envisioned high-throughput magnetic particle separation device that integrates multiple micromagnet chips within a 3D-printed microfluidic housing.

method in this paper is up to two orders of magnitude higher than the separation speed based on simply the magnetic field gradient. We also calculated the speed of the surface roller at 100 Hz as a reference, which is also much slower. The coupling between rotation and translation is approximately 0.2 body length per revolution, which is a value commonly found in the literature.[57]

However, speed is only one factor. As we observed in Figure 5, many other factors (e.g., width of the particle band) are also important for robust high-throughput particle separation. For example, band broadening shows that some particles cannot catch up with the front moving speed and are left behind. This means that for certain applications, we may want to avoid operating in the asynchronous regime and in the synchronous regime at high frequencies. Additionally, increasing the periodicity of the micromagnet array may appear to increase the transport speed. However, it also requires a much higher magnetic field to match the fluidic drag and a sufficient number of microparticles. Depending on the detailed application scenarios, different magnetic particles (e.g., SPIONs, NdFeB, iron, nickel, etc.) with different sizes (from nanometers to millimeters) and surface coatings (-COOH group or with antibodies) can be used in different liquid environments. Further investigation and optimization are required to understand the collective behavior and corresponding separation performance. Taking blood purification applications as an example, the high shear velocity near the chip surface may cause coagulation and generate thrombosis inside the fluidic channel, where surface coating may be important to prevent these problems.

As we discussed in the first section (microfluidic-centered vs. magnet-centered systems), the proposed method not only improves the separation speed of magnetic particles but also provides a larger working volume in which more devices and fluidic channels can be integrated around this given cylindrical magnet. In the future, many micromagnet chips can be assembled into a 3D-printed case with integrated parallel microfluidic connections, as shown in Figure 7E. Due to the inhomogeneity around the spinning NdFeB magnet, compromises must be made between the rotating frequency and the available workspace. And there is certainly room for further optimization of the integration strategy when hundreds of micromagnet chips are integrated into the 3D-printed microfluidic package and the fluidic network inside. Furthermore, we provide a roadmap and design guidelines in the supplemental information (Note S9; Figures S5–S9; Videos S8, S9, and S10) for developing scalable and high-throughput magnetic separation systems from a single microfluidic channel (~0.1 mL/min) to benchtop machines (~100 mL/min). This

massive integration of microfluidic separation devices, utilizing only simple rotating magnets, has the potential to revolutionize current systems by creating compact, cost-effective, and highly efficient systems capable of achieving order-of-magnitude increases in throughput. This development holds significant promise for a wide range of applications, including blood purification, wastewater treatment, compact chemical reactors, DNA scavenging from large liquid samples, and many other scenarios involving the recovery, recycling, and reuse of magnetic micro- and nanorobots.

EXPERIMENTAL PROCEDURES

Resource availability

Lead contact

Further information and requests for resources should be directed to the lead contact, Hongri Gu (hongri.gu@uni-konstanz.de).

Materials availability

This study did not generate new unique materials.

Data and code availability

All data needed to evaluate the conclusions in the paper are present in the paper and the supplemental information. The code that was used to perform the many-particle simulations and the CAD files are available via GitHub (https://github.com/AntonLueders/BDHM) and under the permanent DOI: https://doi.org/10.5281/zenodo.10982927.

SUPPLEMENTAL INFORMATION

Supplemental information can be found online at https://doi.org/10.1016/j.device.2024.100403.

ACKNOWLEDGMENTS

We thank Quan Gao and Dieter Barth for their help in developing the experimental setup. We also thank Wenda Zheng for his advice on figure styling and illustrations. The fabrication of the micromagnet chip samples was performed in the clean room facilities of FIRST at ETH Zurich. We thank the ETH Lab Supporting Group for the deposition process. This work was partially supported by the European Research Council Advanced Grant-Soft MicroRobots (SOMBOT, number 743217), and H.G. would like to acknowledge support from the SNSF Postdoc Mobility Grant (203203). The authors gratefully acknowledge the Gauss Centre for Supercomputing e.V. (https://www.gauss-centre.eu) for funding this project by providing computing time through the John von Neumann Institute for Computing (NIC) on the GCS Supercomputer JUWELS at the Jülich Supercomputing Centre (JSC).

AUTHOR CONTRIBUTIONS

H.G. conceived the idea and managed the research. Y.C., T.B., and H.G. built the setup and performed the experiments. E.H. fabricated the micromagnet chip. A.L. performed the many-particle simulations. H.G., A.L., Y.C., and T.B. analyzed the data. H.G., A.L., and Y.C. drafted the manuscript with contributions from all authors. P.N., C.B., and B.J.N. supervised the project.

DECLARATION OF INTERESTS

The authors declare no competing interests.

Received: December 30, 2023
Revised: January 5, 2024
Accepted: May 1, 2024
Published: June 3, 2024

REFERENCES

1. Urso, M., Ussia, M., and Pumera, M. (2023). Smart micro-and nanorobots for water purification. Nat. Rev. Bioeng. *1*, 236–251. https://doi.org/10.1038/s44222-023-00025-9.
2. Schmidt, C.K., Medina-Sánchez, M., Edmondson, R.J., and Schmidt, O.G. (2020). Engineering microrobots for targeted cancer therapies from a medical perspective. Nat. Commun. *11*, 1–18. https://doi.org/10.1038/s41467-020-19322-7.
3. Rossi, L.M., Costa, N.J.S., Silva, F.P., and Wojcieszak, R. (2014). Magnetic nanomaterials in catalysis: advanced catalysts for magnetic separation and beyond. Green Chem. *16*, 2906–2933. https://doi.org/10.1039/C4GC00164H.
4. Gijs, M.A.M., Lacharme, F., and Lehmann, U. (2010). Microfluidic applications of magnetic particles for biological analysis and catalysis. Chem. Rev. *110*, 1518–1563. https://doi.org/10.1021/cr9001929.
5. Nikoloff, J.M., Saucedo-Espinosa, M.A., Kling, A., and Dittrich, P.S. (2021). Identifying extracellular vesicle populations from single cells. Proc. Natl. Acad. Sci. USA *118*, e2106630118. https://doi.org/10.1073/pnas.2106630118.
6. Bagheri, H., Afkhami, A., Saber-Tehrani, M., and Khoshsafar, H. (2012). Preparation and characterization of magnetic nanocomposite of Schiff base/silica/magnetite as a preconcentration phase for the trace determination of heavy metal ions in water, food and biological samples using atomic absorption spectrometry. Talanta *97*, 87–95. https://doi.org/10.1016/j.talanta.2012.03.066.
7. Bente, K., Bakenecker, A.C., von Gladiss, A., Bachmann, F., Cēbers, A., Buzug, T.M., and Faivre, D. (2021). Selective Actuation and Tomographic Imaging of Swarming Magnetite Nanoparticles. ACS Appl. Nano Mater. *4*, 6752–6759. https://doi.org/10.1021/acsanm.1c00768.
8. Yu, W., Lin, H., Wang, Y., He, X., Chen, N., Sun, K., Lo, D., Cheng, B., Yeung, C., Tan, J., et al. (2020). A ferrobotic system for automated microfluidic logistics. Sci. Robot. *5*, eaba4411. https://doi.org/10.1126/scirobotics.aba4411.
9. Lin, H., Yu, W., A. Sabet, K., Bogumil, M., Zhao, Y., Hambalek, J., Lin, S., Chandrasekaran, S., Garner, O., Di Carlo, D., et al. (2022). Ferrobotic swarms enable accessible and adaptable automated viral testing. Nature *611*, 570–577. https://doi.org/10.1038/s41586-022-05408-3.
10. Xie, H., Sun, M., Fan, X., Lin, Z., Chen, W., Wang, L., Dong, L., and He, Q. (2019). Reconfigurable magnetic microrobot swarm: Multimode transformation, locomotion, and manipulation. Sci. Robot. *4*, eaav8006. https://doi.org/10.1126/scirobotics.aav8006.
11. Rittich, B., and Spanová, A. (2013). SPE and purification of DNA using magnetic particles. J. Separ. Sci. *36*, 2472–2485. https://doi.org/10.1002/jssc.201300331.
12. Dubus, S., Gravel, J.-F., Le Drogoff, B., Nobert, P., Veres, T., and Boudreau, D. (2006). PCR-free DNA detection using a magnetic bead-supported polymeric transducer and microelectromagnetic traps. Anal. Chem. *78*, 4457–4464. https://doi.org/10.1021/ac060486n.
13. Xiang, D.s., Zeng, G.p., and He, Z.k. (2011). Magnetic microparticle-based multiplexed DNA detection with biobarcoded quantum dot probes. Biosens. Bioelectron. *26*, 4405–4410. https://doi.org/10.1016/j.bios.2011.04.051.
14. Labib, M., Wang, Z., Ahmed, S.U., Mohamadi, R.M., Duong, B., Green, B., Sargent, E.H., and Kelley, S.O. (2021). Tracking the expression of therapeutic protein targets in rare cells by antibody-mediated nanoparticle labelling and magnetic sorting. Nat. Biomed. Eng. *5*, 41–52. https://doi.org/10.1038/s41551-020-0590-1.
15. Mishra, A., Dubash, T.D., Edd, J.F., Jewett, M.K., Garre, S.G., Karabacak, N.M., Rabe, D.C., Mutlu, B.R., Walsh, J.R., Kapur, R., et al. (2020). Ultrahigh-throughput magnetic sorting of large blood volumes for epitope-agnostic isolation of circulating tumor cells. Proc. Natl. Acad. Sci. USA *117*, 16839–16847. https://doi.org/10.1073/pnas.2006388117.

16. Herrmann, I.K., Schlegel, A.A., Graf, R., Stark, W.J., and Beck-Schimmer, B. (2015). Magnetic separation-based blood purification: a promising new approach for the removal of disease-causing compounds? J. Nanobiotechnol. *13*, 49–54. https://doi.org/10.1186/s12951-015-0110-8.

17. Herrmann, I.K., Urner, M., Koehler, F.M., Hasler, M., Roth-Z'Graggen, B., Grass, R.N., Ziegler, U., Beck-Schimmer, B., and Stark, W.J. (2010). Blood purification using functionalized core/shell nanomagnets. Small *6*, 1388–1392. https://doi.org/10.1002/smll.201000438.

18. Kang, J.H., Super, M., Yung, C.W., Cooper, R.M., Domansky, K., Graveline, A.R., Mammoto, T., Berthet, J.B., Tobin, H., Cartwright, M.J., et al. (2014). An extracorporeal blood-cleansing device for sepsis therapy. Nat. Med. *20*, 1211–1216. https://doi.org/10.1038/nm.3640.

19. Kim, D., Kwon, H.J., and Hyeon, T. (2019). Magnetite/Ceria Nanoparticle Assemblies for Extracorporeal Cleansing of Amyloid-β in Alzheimer's Disease. Adv. Mater. *31*, 1807965. https://doi.org/10.1002/adma.201807965.

20. Jang, J., and Park, C.B. (2022). Magnetoelectric dissociation of Alzheimer's $β$-amyloid aggregates. Sci. Adv. *8*, eabn1675. https://doi.org/10.1126/sciadv.abn1675.

21. Majumder, A., Gupta, A.K., Ghosal, P.S., and Varma, M. (2021). A review on hospital wastewater treatment: A special emphasis on occurrence and removal of pharmaceutically active compounds, resistant microorganisms, and SARS-CoV-2. J. Environ. Chem. Eng. *9*, 104812. https://doi.org/10.1016/j.jece.2020.104812.

22. Pariente, M.I., Segura, Y., Álvarez-Torrellas, S., Casas, J.A., de Pedro, Z.M., Diaz, E., García, J., López-Muñoz, M.J., Marugán, J., Mohedano, A.F., et al. (2022). Critical review of technologies for the on-site treatment of hospital wastewater: From conventional to combined advanced processes. J. Environ. Manag. *320*, 115769. https://doi.org/10.1016/j.jenvman.2022.115769.

23. Zhou, H., Mayorga-Martinez, C.C., Pané, S., Zhang, L., and Pumera, M. (2021). Magnetically driven micro and nanorobots. Chem. Rev. *121*, 4999–5041. https://doi.org/10.1021/acs.chemrev.0c01234.

24. González Fernández, C., Gómez Pastora, J., Basauri, A., Fallanza, M., Bringas, E., Chalmers, J.J., and Ortiz, I. (2020). Continuous-flow separation of magnetic particles from biofluids: how does the microdevice geometry determine the separation performance? Sensors *20*, 3030. https://doi.org/10.3390/s20113030.

25. Pamme, N., Eijkel, J.C., and Manz, A. (2006). On-chip free-flow magnetophoresis: Separation and detection of mixtures of magnetic particles in continuous flow. J. Magn. Magn Mater. *307*, 237–244. https://doi.org/10.1016/j.jmmm.2006.04.008.

26. Nguyen, J., Conca, D.V., Stein, J., Bovo, L., Howard, C.A., and Llorente Garcia, I. (2019). Magnetic control of graphitic microparticles in aqueous solutions. Proc. Natl. Acad. Sci. USA *116*, 2425–2434. https://doi.org/10.1073/pnas.1817989116.

27. Robert, D., Pamme, N., Conjeaud, H., Gazeau, F., Iles, A., and Wilhelm, C. (2011). Cell sorting by endocytotic capacity in a microfluidic magnetophoresis device. Lab Chip *11*, 1902–1910. https://doi.org/10.1039/C0LC00656D.

28. Pamme, N., and Wilhelm, C. (2006). Continuous sorting of magnetic cells via on-chip free-flow magnetophoresis. Lab Chip *6*, 974–980. https://doi.org/10.1039/B604542A.

29. Gu, S.-Q., Zhang, Y.-X., Zhu, Y., Du, W.-B., Yao, B., and Fang, Q. (2011). Multifunctional picoliter droplet manipulation platform and its application in single cell analysis. Anal. Chem. *83*, 7570–7576. https://doi.org/10.1021/ac201678g.

30. Vermesh, O., Aalipour, A., Ge, T.J., Saenz, Y., Guo, Y., Alam, I.S., Park, S.M., Adelson, C.N., Mitsutake, Y., Vilches-Moure, J., et al. (2018). An intravascular magnetic wire for the high-throughput retrieval of circulating tumour cells in vivo. Nat. Biomed. Eng. *2*, 696–705. https://doi.org/10.1038/s41551-018-0257-3.

31. Alnaimat, F., Dagher, S., Mathew, B., Hilal-Alnqbi, A., and Khashan, S. (2018). Microfluidics based magnetophoresis: A review. Chem. Rec. *18*, 1596–1612. https://doi.org/10.1002/tcr.201800018.

32. Su, R., Wang, F., and McAlpine, M.C. (2023). 3D printed microfluidics: advances in strategies, integration, and applications. Lab Chip *23*, 1279–1299. https://doi.org/10.1039/D2LC01177H.

33. Zhu, Y., and Fang, Q. (2013). Analytical detection techniques for droplet microfluidics—A review. Anal. Chim. Acta *787*, 24–35. https://doi.org/10.1016/j.aca.2013.04.064.

34. Elliott, D.A. (2000). Hemodialysis. Clin. Tech. Small Anim. Pract. *15*, 136–148. https://doi.org/10.1053/svms.2000.18297.

35. Kim, Y.O., Song, W.J., Yoon, S.A., Shin, M.J., Song, H.C., Kim, Y.S., Kim, S., Chang, Y., Bang, B., and Bang, B. (2004). The effect of increasing blood flow rate on dialysis adequacy in hemodialysis patients with low Kt/V. Hemodial. Int. *8*, 85. https://doi.org/10.1111/j.1492-7535.2004.0085q.x.

36. Lin, S.-L., Huang, C.-H., Chen, H.-S., Hsu, W.-A., Yen, C.-J., and Yen, T.-S. (1998). Effects of age and diabetes on blood flow rate and primary outcome of newly created hemodialysis arteriovenous fistulas. Am. J. Nephrol. *18*, 96–100. https://doi.org/10.1159/000013315.

37. Nelson, B.J., Kaliakatsos, I.K., and Abbott, J.J. (2010). Microrobots for minimally invasive medicine. Annu. Rev. Biomed. Eng. *12*, 55–85. https://doi.org/10.1146/annurev-bioeng-010510-103409.

38. Abbott, J.J., Diller, E., and Petruska, A.J. (2020). Magnetic methods in robotics. Annu. Rev. Control. Robot. Autonom. Syst. *3*, 57–90. https://doi.org/10.1146/annurev-control-081219-082713.

39. Abbott, J.J., Peyer, K.E., Lagomarsino, M.C., Zhang, L., Dong, L., Kaliakatsos, I.K., and Nelson, B.J. (2009). How should microrobots swim? Int. J. Robot Res. *28*, 1434–1447. https://doi.org/10.1177/0278364909341658.

40. Ge, W., Encinas, A., Araujo, E., and Song, S. (2017). Magnetic matrices used in high gradient magnetic separation (HGMS): A review. Results Phys. *7*, 4278–4286. https://doi.org/10.1016/j.rinp.2017.10.055.

41. Pamme, N. (2006). Magnetism and microfluidics. Lab Chip *6*, 24–38. https://doi.org/10.1039/B513005K.

42. Zhang, Y., and Nguyen, N.-T. (2017). Magnetic digital microfluidics–a review. Lab Chip *17*, 994–1008. https://doi.org/10.1039/C7LC00025A.

43. Jamshaid, T., Neto, E.T.T., Eissa, M.M., Zine, N., Kunita, M.H., El-Salhi, A.E., and Elaissari, A. (2016). Magnetic particles: From preparation to lab-on-a-chip, biosensors, microsystems and microfluidics applications. TrAC Trends Anal. Chem. *79*, 344–362. https://doi.org/10.1016/j.trac.2015.10.022.

44. Afshar, R., Moser, Y., Lehnert, T., and Gijs, M.A.M. (2011). Magnetic particle dosing and size separation in a microfluidic channel. Sens. Actuators, B *154*, 73–80. https://doi.org/10.1016/j.snb.2009.08.044.

45. Sing, C.E., Schmid, L., Schneider, M.F., Franke, T., and Alexander-Katz, A. (2010). Controlled surface-induced flows from the motion of self-assembled colloidal walkers. Proc. Natl. Acad. Sci. USA *107*, 535–540. https://doi.org/10.1073/pnas.0906489107.

46. Tasci, T.O., Herson, P.S., Neeves, K.B., and Marr, D.W.M. (2016). Surface-enabled propulsion and control of colloidal microwheels. Nat. Commun. *7*, 10225–10226. https://doi.org/10.1038/ncomms10225.

47. Kummer, M.P., Abbott, J.J., Kratochvil, B.E., Borer, R., Sengul, A., and Nelson, B.J. (2010). OctoMag: An electromagnetic system for 5-DOF wireless micromanipulation. IEEE Trans. Robot. *26*, 1006–1017. https://doi.org/10.1109/TRO.2010.2073030.

48. Gu, H., Hanedan, E., Boehler, Q., Huang, T.-Y., Mathijssen, A.J.T.M., and Nelson, B.J. (2022). Artificial microtubules for rapid and collective transport of magnetic microcargoes. Nat. Mach. Intell. *4*, 678–684. https://doi.org/10.1038/s42256-022-00510-7.

49. Ryan, P., and Diller, E. (2017). Magnetic actuation for full dexterity microrobotic control using rotating permanent magnets. IEEE Trans. Robot. *33*, 1398–1409. https://doi.org/10.1109/TRO.2017.2719687.

50. Tierno, P., Sagués, F., Johansen, T.H., and Fischer, T.M. (2009). Colloidal transport on magnetic garnet films. Phys. Chem. Chem. Phys. *11*, 9615–9625. https://doi.org/10.1039/B910427E.

51. Leyva, S.G., Stoop, R.L., Pagonabarraga, I., and Tierno, P. (2022). Hydrodynamic synchronization and clustering in ratcheting colloidal matter. Sci. Adv. *8*, eabo4546. https://doi.org/10.1126/sciadv.abo4546.

52. Lauga, E., and Powers, T.R. (2009). The hydrodynamics of swimming microorganisms. Rep. Prog. Phys. *72*, 096601. https://doi.org/10.1088/0034-4885/72/9/096601.

53. Elgeti, J., Winkler, R.G., and Gompper, G. (2015). Physics of microswimmers—single particle motion and collective behavior: a review. Rep. Prog. Phys. *78*, 056601. https://doi.org/10.1088/0034-4885/78/5/056601.

54. Yu, J., Jin, D., Chan, K.-F., Wang, Q., Yuan, K., and Zhang, L. (2019). Active generation and magnetic actuation of microrobotic swarms in bio-fluids. Nat. Commun. *10*, 5631. https://doi.org/10.1038/s41467-019-13576-6.

55. Law, J., Wang, X., Luo, M., Xin, L., Du, X., Dou, W., Wang, T., Shan, G., Wang, Y., Song, P., et al. (2022). Microrobotic swarms for selective embolization. Sci. Adv. *8*, eabm5752. https://doi.org/10.1126/sciadv.abm5752.

56. Hwang, G., Paula, A.J., Hunter, E.E., Liu, Y., Babeer, A., Karabucak, B., Stebe, K., Kumar, V., Steager, E., and Koo, H. (2019). Catalytic antimicrobial robots for biofilm eradication. Sci. Robot. *4*, eaaw2388. https://doi.org/10.1126/scirobotics.aaw2388.

57. Chamolly, A., Lauga, E., and Tottori, S. (2020). Irreversible hydrodynamic trapping by surface rollers. Soft Matter *16*, 2611–2620. https://doi.org/10.1039/C9SM02250C.

DEVICE, Volume *2*

Supplemental information

Scalable high-throughput microfluidic separation of magnetic microparticles

Hongri Gu, Yonglin Chen, Anton Lüders, Thibaud Bertrand, Emre Hanedan, Peter Nielaba, Clemens Bechinger, and Bradley J. Nelson

Note S1 Magnetic particles and properties

We use Micromer®-M (Micromod, 08-02-104, spherical with 10 µm size, surface: COOH, suspension in water, concentration: 50 $\mathrm{mg/mL}$, magnetization: 1.8 $\mathrm{A\,m^2/kg}$ at $H = 80\mathrm{kA/m}$, price: 264 euro for 10 mL) as the magnetic microparticle in the experiments. We then dilute the original particle suspension tenfold to become the input fluid that we use in the externally driven flow experiments depicted in Fig. 6 of the main article. Therefore, the particle concentration of the input fluid is 5 $\mathrm{mg/mL}$ (approx. 8.7×10^6 particles per mL). Due to the sedimentation, the particle concentration may slightly vary during the experiments.

Note S2 Design of the microfluidic device for magnetic particle separation

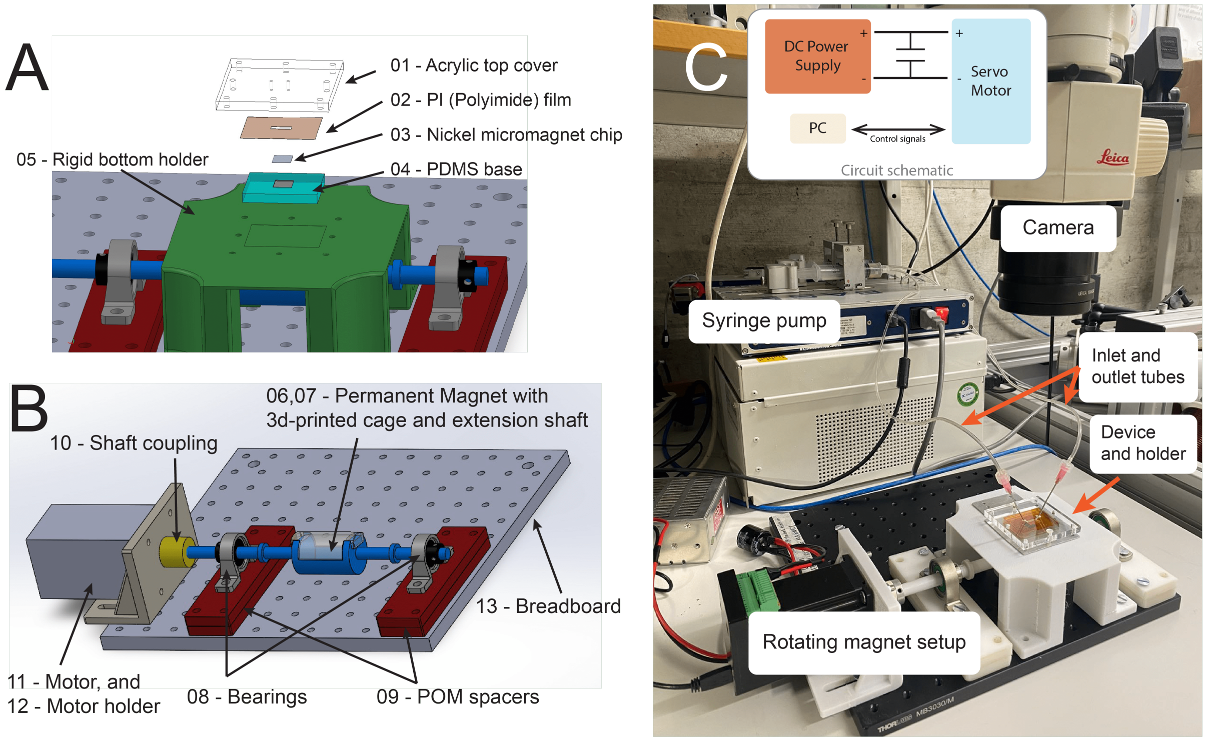

Figure S1. Design of the microfluidic particle separation device and the experimental setup. A.) The exploded view of the microfluidic separation device. The size of fluid channel (10 mm × 3 mm × 25.4 µm) is determined by the laser cut polyimide film. Eight M3 bolts and nuts are used to ensure a tight sealing between the acrylic top cover and the 3D-printed rigid bottom holder. B.) Assembly drawing of rotating magnet setup. We use a cylindrical NdFeB with radial magnetization (diameter = 30 mm and height = 40 mm). C.) Photograph of the complete experimental setup, with components labeled. The inlet and outlet tubes are connected to the syringe pump. The connection of the motor driver circuit is shown in the circuit schematic. The speed of the servo motor is controlled by a software (Plug & Drive Studio, Nanotec) on the PC.

We tested the magnetic particle separation in a microfluidic system shown in Fig. S1. The dimensions of the microfluidic channel are 10 mm × 3 mm × 25.4 µm (length × width × height). Fig. S1A demonstrates the assembly of the microfluidic separation device. It consists of 5 components (from top to bottom, as the numbers shown in Fig. S1A): 1) a 3-mm acrylic cover; 2) polyimide (Kapton) film (Thickness: 25.4 µm, McMaster-Carr, IL, USA); 3) nickel micromagnet chip; 4) PDMS base; 5) 3D-printed poly-carbonate rigid bottom holder, with holes for M6 screws on its legs for fixation on the breadboard. These layers are fixed with M3 bolts and nuts to ensure that the channel height is equal to the polyimide film. And soft PDMS layer ensures a tight fitting between the layers. We do not observe noticeable leakage even under a high flow rate at 3000 $\mathrm{\mu L/min}$.

The main objectives of the device design are to ensure height retention, anti-leakage, and good optical observability.

1) Height retention: The height of the fluid channel has a significant impact on the performance of particle separation. Therefore, ensuring a fixed and precise height control becomes an important consideration of device

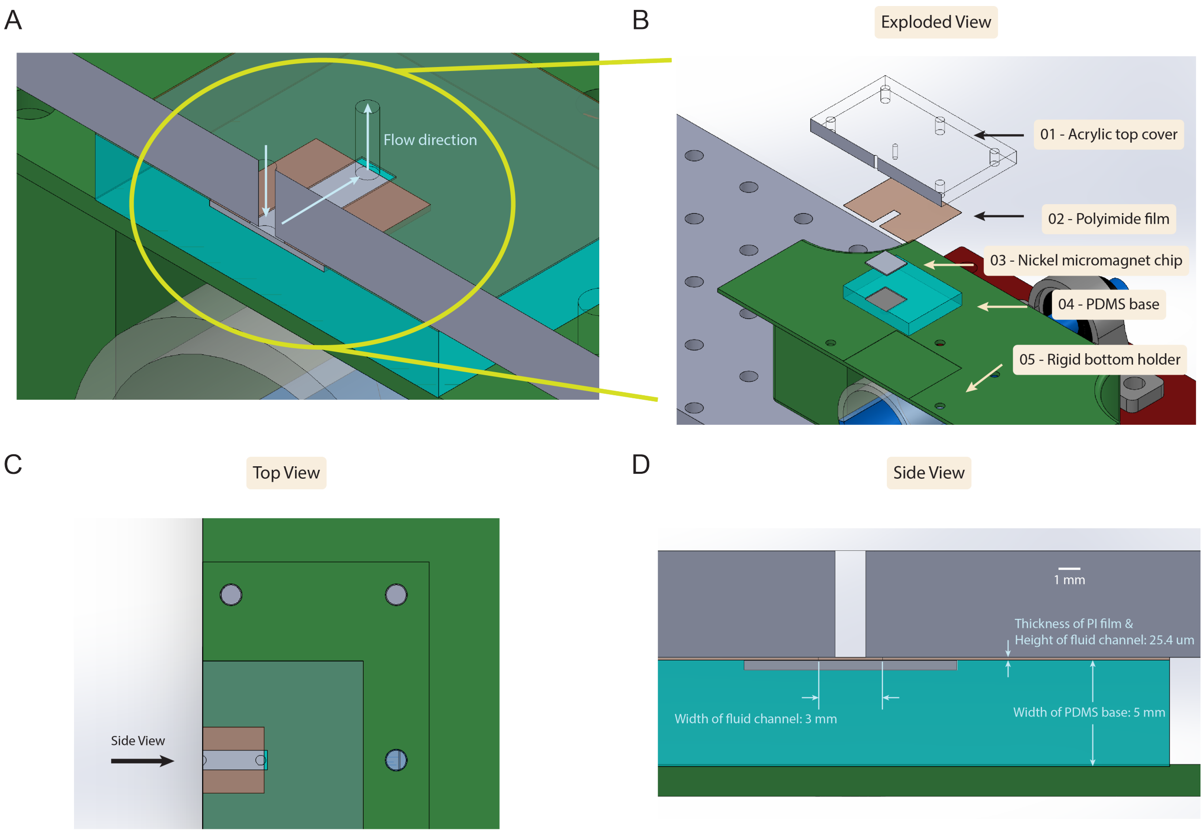

Figure S2. Details of fluid channel design. A.) 3D-sectional view of the fluid channel in Solidworks. The flow direction is marked by light blue arrows. The inlet and outlet directly sit on the fluid channel and guide the fluid through the channel, preventing leakage and contact with the PDMS base. B.) Exploded view of the fluid channel. The depth of the slot on the PDMS base is the same as the thickness of the chip. Hence, the upper surface of the chip and PDMS base is flat. The central rectangular slot in the PI film defines the dimensions of the fluid channel. C.) Top view of the sectional view of the microfluidic device. D.) Side view of the microfluidic device, reflecting the true scale of different parts in the device.

design. We use a polyimide (PI) film (Thickness: 25.4 μm, McMaster-Carr, IL, USA) to provide rigid support between the top acrylic cover and the soft PDMS base. The rectangular slit in the center of the film defines the dimensions of the fluid channel. (Fig. S2) Therefore, by tightening the bolts and nuts around the PDMS base and applying enough pressure on the Acrylic-PI film-PDMS interfaces, the height of the fluid channel is the same as the thickness of the PI film.

2) Anti-leakage: During the experiments, the device needs to be disassembled and cleaned once in a while, to get rid of the residual microparticles attached to the surfaces of the fluid channel. Therefore, the bonding between different parts of the microfluidic device cannot be permanent. Under the above constraints, preventing leakage and maintaining a fixed height of the fluid channel can be challenging.

Additionally, the inlet and outlet are located vertically above the fluid channel and guide the fluid through the channel without contacting the PDMS base. (Fig. S2A) Under sufficient pressure, the large contact area between the PI film and the PDMS base surrounding the fluid channel also helps prevent liquid from leaking out of the channel. Therefore, when sufficient pressure is applied by tightening the bolts and nuts, the interfaces between acrylic cover - polyimide film - PDMS are tight preventing leakage even at a high flow rate (typically 2000-3000 $\mu L/min$) without any adhesion agent.

3) Optical observability: The observation and recording of the dynamics of swarm microparticles are performed with a microscope placed above the setup. By covering a 3 mm-thick acrylic plate as the top cover, the fluid channel and magnetic particles are entirely visible from above. The details are shown in Fig. S1A.

4) Bubble-free: The height of the fluid channel (tens of microns) is several times smaller than its length and width. When the flow passes by, it is necessary to remove all bubbles inside the fluidic channel. The fluid inlet and

Table S1. Characteristic dimensions of components in the microfluidic separation device.

No #	Item	Characteristic dimensions
01	Acrylic top cover	Outline: 60 mm $\times$ 60 mm $\times$ 3 mm, holes for inlet/outlet: d1.5 mm
02	Polyimide film	Outline: 40 mm $\times$ 30 mm $\times$ 25.4 µm, Slot: 3 mm $\times$ 11 mm
03	Nickel micromagnet chip	Outline: 10 mm $\times$ 10 mm $\times$ 460 µm
04	PDMS base	Outline: 40 mm $\times$ 30 mm $\times$ 5 mm, Slot: same as the chip
05	3D-printed polycarbonate bottom holder	Thickness: 1.5 mm

outlet are placed directly above the chip so that the flow does not pass the gap between the PDMS and the chip. In practice, we control the flow rate and wet the channel first to make sure the system is bubble-free.

Note S3 Design of the rotating magnetic field setup

Fig. S1B shows the assembly of the rotating magnetic field setup. We use a cylindrical NdFeB magnet with radial magnetization (HKCM, 9962-72557, flux density at surface = 439 mT) inside a 3D-printed poly-carbonate cage with a shaft connected to a stepper motor (Nanotec, PD4-C5918L4204-E-01) with a shaft coupling (SUNGIL, SJC-25C-GR-6X10). The whole structure is supported by 2 bearing units (MISUMI pillow blocks, PBRSX10) to ensure a smooth rotation.

Circuit schematic of the servo motor system: As shown in Fig. S1C), the electrical connection of the motor is as follows: The DC power supply provides the power for the motor. An electrolyte capacitor is connected in parallel with the DC power supply. The motor has a built-in controller and driver. The control signals are sent from the PC to the motor via a USB connection.

Control of the motor speed: The manufacturer of the servo motor (Nanotec) provides software (Plug & Drive Studio) to directly set and control the speed of the motor in a GUI. No custom code is used to control the motor speed.

Note S4 Externally driven flow setup

Fig. S1C shows the structure of the experimental setup. A syringe pump (Cetoni Base 120) is used to control the flow rate. Two 18-gauge needles are inserted and glued on the acrylic cover of the microfluidic device as the fluid inlet and outlet. A microscope and camera (Basler acA2440-35uc) are mounted above to observe the particle swarm dynamics with and without the external flow.

In the externally driven flow experiment, we use a syringe to extract around 5 mL of stirred particle suspension and mount it on the syringe pump. An identical empty syringe is mounted on the other syringe pump to collect the liquid from the outlet. We wait 20 to 30 seconds for the system to reach equilibrium after changing the flow parameters. The nickel micromagnet chip is cleaned and re-used after each experiment.

Note S5 Magnetic flux density measurement

The magnetic field is measured using a customized setup as shown in Fig. S3A. A 3-axis magnetic field sensor (Metrolab, THM1176 Hall Magnetometer) is fixed on a POM plate mounted on a motorized XYZ stage. The cylindrical NdFeB magnet rotates at $f = 0.3$ Hz and the hall sensor records the waveform $B(t)$. The recorded flux density waveform is sinusoidal, as shown in Fig. S4. The sensor is adjusted to a specific vertical height and the average value of the waveform is calculated as the magnetic field strength at this height. Fig. S3B shows the curve of the averaged magnetic flux density B at different vertical distances r. In this curve, r is defined as the vertical distance between the surface of the magnet cage (not the magnet itself) and the bottom surface of the POM plate that carries the hall sensor. The POM plate is 4 mm thick. As the curve shows, the field strength decreases rapidly with an increasing r.

Note S6 Numerical simulations of the magnetic field around micromagnet array

The magnetic field around the nickel micromagnet chip is simulated using COMSOL Multiphysics. In the simulation, we assume that the permeability of the nickel micromagnets is 70 and the rest is 1. A simple stationary

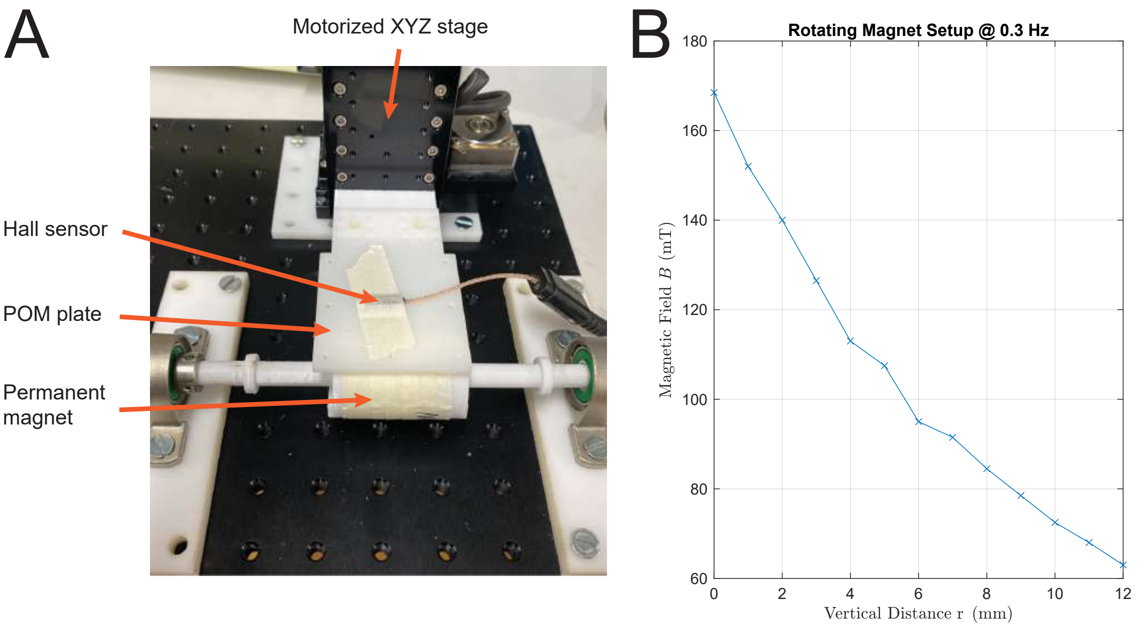

Figure S3. Magnetic field measurement near the rotating cylindrical magnet using a Hall sensor. A.) Photo of the setup for measuring the average flux density of the rotating permanent magnet. B.) Curve of multi-cycle averaged magnetic flux density versus vertical distance. The cylindrical magnet rotates along its axis at $f = 0.3$ Hz. The average magnetic flux density in the externally driven flow experiment is 130 mT.

case is implemented for magnetic fields in different directions using a simple model (Magnetic Fields, No Currents, mfnc). A uniform magnetic flux of 100 mT is applied to all outer surfaces of the geometries, and the results are visualized in Fig. 3D of the main article. In the visualization, we selected streamline positioning only on the surfaces of the micromagnets with adjusted streamline densities to provide an easier understanding of the 3D magnetic field distribution. The streamline density in Fig. 3D is only a qualitative representation of the local field strength.

Note S7 Comparison between different magnetic separation methods

We compare our collective transport of magnetic microparticles with two commonly used separation methods: (1) drifting based on the magnetic field gradient, and (2) surface roller under a rotating magnetic field. There are many factors in the experimental implementation of these systems that can affect the overall throughput of magnetic microparticle separation. In this work, we focus on a microscopic quantity, i.e., magnetic particle velocity, which eliminates the influence of the microfluidic channel dimensions. We believe that it more accurately reflects the differences in the performance of the separation methods.

The other important factor that makes comparison difficult is the different magnetic setups in the literature. For example, one can place a very powerful superconducting magnet near the microfluidic channel and achieve record throughput. However, this unique advantage cannot be easily replicated by other users considering implementation in other applications. Here, we assume that all methods can only use the identical cylindrical NdFeB magnet (grade: N35, diameter: 3 cm, length: 4 cm) and compare the corresponding moving speed of the magnetic microparticles.

We simulated the magnetic field around the cylindrical magnet in COMSOL Multiphysics (magnetic flux density $B_r = 1192$ mT) and used curve fitting (power law, as for a point dipole) to numerically calculate the corresponding magnetic field gradient. The gradient is used to calculate the moving speed of the magnetic microparticles utilized in this study, based on the reported size and magnetic properties (10 μm diameter, magnetization: 1.8 $\mathrm{A\,m^2/kg}$ at $H = 80\mathrm{kA/m}$). For the surface rollers, we assume that they are actively rotating at 100 Hz, which is usually considered a very high speed in the literature. We use the translation to rotation coupling factor $\alpha = 0.1$, which means that the magnetic particle moves 0.1 times its diameter under a single rotation, which is commonly reported in the literature for roller on a smooth surface.

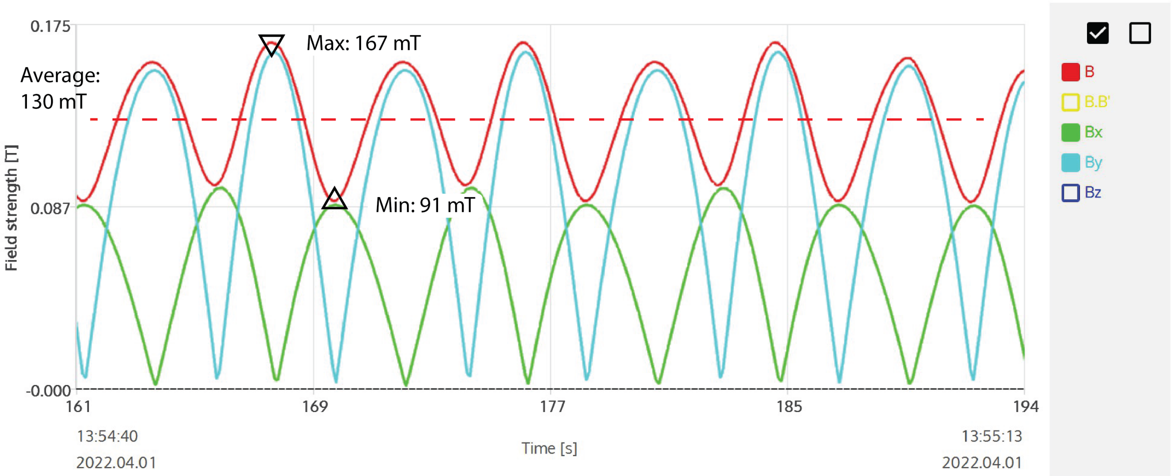

Figure S4. Recorded waveform of magnetic flux density near the rotating cylindrical magnet using a 3-axis hall sensor. The rotating frequency of the cylindrical magnet is 0.3 Hz, and the waveform of the total magnetic flux density B is sinusoidal. The average, maximum, and minimum values are 130, 167, and 91 mT, respectively. We use the average value to represent the magnetic field strength in experiments and numerical simulations (Note that the field strength is kept constant in the many-particle simulations).

Note S8 Many-particle Simulation

Various aspects that contribute to the dynamics of the systems (such as the hydrodynamic interactions between the particles and the walls, the magnetic field resulting from the micromagnet array, the additionally applied flow, and the external magnetic field with changing direction and magnitude) result in a high complexity of our setup. To break this complexity down and enhance the generality of our results, we implement a heavily simplified minimal toy model to corroborate our experimental findings.

In our simulations, we model the superparamagnetic microparticles as hydrodynamic point particles that possess a magnetic (point) dipole moment. To incorporate the finite volume of the real colloids (i.e., steric interactions) and avoid diverging dipole forces, we add purely repulsive pair interactions to the particle dynamics. However, hydrodynamic interactions that would result from these interactions are neglected to increase the stability of the simulations. This simplification leads to unphysical motions of "touching" particles, but the excellent qualitative agreement between our numerical model and the results of the experiments shows that our assumptions are sufficient to capture the collective phenomena sufficiently.

The micromagnets of the array are also modeled as magnetic point dipoles. To approximate their elongated shape, we assign the magnetic anisotropy of a prolate spheroid to the dipole moments by incorporating an (apparent) susceptibility tensor for magnetic ellipsoids in our corresponding calculations. We use a "wall" to separate the micromagnets and the superparamagnetic colloids, which is implemented as standard soft wall boundary conditions. Here, we use the same repulsive pair potential as for the repulsion between the microparticles[S1, S2]. The influence of the wall on the hydrodynamic interactions between the colloids is accounted for by applying the Blake tensor[S3] (for hydrodynamically interacting point particles in front of a wall) in our equations of motion. In our numerical model, we simplify the rotating external magnetic field by using a constant magnitude. Note that we assume that the magnetic moments of both, the particles and the micromagnets are linearly related to the external field for all applied field strengths, and no saturation magnetizations are reached.

For the simulations without an additional flow field, we utilize periodic boundary conditions in all directions that are not restricted by the soft wall separating the micromagnet array from the particles, where we always assume that the colloids only interact with their nearest periodic image. In these systems, the initial positions of the particles are randomly chosen at one "end" of the array. In the studies with an additional flow field, we add more walls to the systems: Firstly, we implement an additional soft wall parallel to the micromagnet array to set the constriction for the parabolic Poiseuille flow[S4] that we include in the equations of motion. Secondly, we include a soft wall at the end of the micromagnet array to which the particles are driven using the rotating magnetic field. The influence of both walls on the hydrodynamic interactions between the spheres is neglected. In the simulations with the additional flow field, we have to continuously add and remove new spheres to address the particle transport. For this, we use

a simple model where we delete particles that would leave the micromagnet array and spawn new particles at a constant rate along a predefined boundary.

Equations of motion: To describe the motion of the superparamagnetic particles, we integrate the Stokes law-like equations that connect the velocities of the colloids with the forces that act on them (through the grand mobility tensor)[S5]. This is in line with works such as Refs. S6, S7. Applying our additional approximations (for instance, neglecting the hydrodynamic interactions for the steric pair interactions of touching colloids), we find the integrator

$$\vec{r}_i(t+\Delta t) = \vec{r}_i(t) + \sum_j \hat{\mu}_{ij}(t)\vec{F}_j^{\text{mag}}(t)\Delta t + \frac{D}{k_B T}\vec{F}_i^{\text{ste}}(t)\Delta t + \vec{v}_i^{\text{Poi}}(t)\Delta t \tag{S1}$$

for the position $\vec{r}_i$ of the ith sphere. Here, Δt is the length of the simulation step, $\hat{\mu}_{ij}$ is the hydrodynamic mobility tensor[S5] which depends on the positions of all particles and is based on the Blake tensor[S3] for point particles, $\vec{F}_j^{\text{mag}}$ is the sum of the magnetic forces acting on particle j, $\vec{F}_i^{\text{ste}}$ is the sum of the repulsive pair interactions acting on particle i (i.e., all particle-particle and particle-wall repulsions) and $\vec{v}_i^{\text{Poi}}$ is the Poiseuille flow field[S4] that can be engaged. Note that the Poiseuille flow $\vec{v}_i^{\text{Poi}}(t)$ depends on the position of the ith sphere at time t. The constants D and k_B are the diffusion coefficient of a sphere and the Boltzmann constant, respectively. The quantity T is the temperature.

Magnetic properties: After applying the external magnetic field, the superparamagnetic particles and micromagnets carry a magnetic dipole moment. In the real system, the direction and magnitude of these moments should be determined by the total magnetic field given by the external field and the dipole moments of the surrounding particles and micromagnets[S7]. In our simple toy model, however, we neglect the influence the colloids and micromagnets have on each other and assume that only the external magnetic field is relevant for the particular moments at a specific time. We further estimate that the orientation of the particles and the moments follow the orientation of the external rotating field instantaneously, and there are no phenomena such as hysteresis. Additionally, we assume that the external field and the magnetic moments of the components are linearly related for all applied field magnitudes (i.e., we do not reach saturation magnetizations).

At a specific time t, all superparamagnetic particles possess an identical magnetic moment $\vec{m}^{col}(t)$. In the same manner, all micromagnets of the array possess an identical magnetic moment $\vec{m}^{pol}(t)$ (but $\vec{m}^{col}(t) \neq \vec{m}^{pol}(t)$). Through simple algebraic transformations, we can express the magnetic moments of the micromagnets in multiples of the dipole moments of the particles. For this, we write

$$\vec{m}^{col}(t) = m_0 \vec{\hat{H}}(t) \tag{S2}$$

for the moment which each colloid holds, where m_0 is the corresponding magnitude and $\vec{\hat{H}}(t) = \vec{H}(t)/|\vec{H}(t)|$ is the direction of the external magnetic field $\vec{H}(t)$. Then, the magnetic moment of the micromagnets is

$$\vec{m}^{pol}(t) = \alpha^{\text{pol}} m_0 \hat{\chi} \vec{\hat{H}}(t). \tag{S3}$$

Here, $\hat{\chi}$ is the diagonal (apparent) susceptibility tensor of an ellipsoid[S8] with the two unique eigenvalues

$$\chi^{\parallel} = \frac{\chi_0}{1+\chi_0 n^{\parallel}} \tag{S4}$$

$$\chi^{\perp} = \frac{\chi_0}{1+\chi_0 n^{\perp}} \tag{S5}$$

that correspond to the directions parallel and perpendicular to the orientation of the elongated micromagnets (which we model as ellipsoids). The corresponding quantity χ_0 is the susceptibility of the material, and we use the demagnetization factors

$$n^{\parallel} = \frac{1}{q^2-1}\left[\frac{q}{2\sqrt{q^2-1}} \ln\left(\frac{q+\sqrt{q^2-1}}{q-\sqrt{q^2-1}}\right) - 1\right] \tag{S6}$$

$$n^{\perp} = \frac{1-n^{\parallel}}{2} \tag{S7}$$

depending on the aspect ratio q of the micromagnets[S8] ($\chi^{\perp}$ is a double eigenvalue). The dimensionless coefficient α^{pol} is given by

$$\alpha^{\mathrm{pol}} = \frac{V^{\mathrm{pol}} H}{m_0}, \tag{S8}$$

where V^{pol} is the volume of a micromagnent.

The force resulting from the interaction between two magnetic moments $\vec{m}_i$ and $\vec{m}_j$ is computed as usually[S6, S7] using the relation for magnetic point dipoles

$$\vec{F}_{ij} = F_0^{\mathrm{mag}} \left(\frac{\sigma}{|\vec{r}_{ij}|}\right)^4 \left\{ \left(\frac{\vec{\tilde{m}}_i \cdot \vec{r}_{ij}}{|\vec{r}_{ij}|}\right) \vec{\tilde{m}}_j + \left(\frac{\vec{\tilde{m}}_j \cdot \vec{r}_{ij}}{|\vec{r}_{ij}|}\right) \vec{\tilde{m}}_i - \left[\left(\frac{5\,(\vec{\tilde{m}}_i \cdot \vec{r}_{ij})\,(\vec{\tilde{m}}_j \cdot \vec{r}_{ij})}{|\vec{r}_{ij}|^2} \right) - (\vec{\tilde{m}}_i \cdot \vec{\tilde{m}}_j) \right] \frac{\vec{r}_{ij}}{|\vec{r}_{ij}|} \right\}, \tag{S9}$$

which contains the vector $\vec{r}_{ij}$ connecting the point dipoles, the diameter σ of the spheres and the dipole interaction strength

$$F_0^{\mathrm{mag}} = \frac{3\mu_0 m_0^2}{4\pi\sigma^4}, \tag{S10}$$

where μ_0 is the vacuum permeability. Here, we used the abbreviations $\vec{\tilde{m}}_i(t) = \vec{m}_i(t)/m_0$ and $\vec{\tilde{m}}_j(t) = \vec{m}_j(t)/m_0$ marking the directions of the moments. Equation (S9) can be used for the magnetic interactions between two particles and the magnetic interactions between a particle and a micromagnet.

Especially in the simulations with the additional flow field, the numerical systems consist of a large number of particles and micromagenets. To save computational cost, we introduce cut-off radii describing distances after which we neglect the dipole interactions. For the colloid-micromagnet interactions, we use 3.5 times the lattice constant of the micromagnet array for said distance. For the dipole interactions between the particular microparticles, we use a cut-off radius of 2.5 times the lattice constant of the micromagnet array. Note that we apply these hard-coded cut-off radii independently of the dimensions of the simulation box or the micromagnet array and neglect interactions with more than the nearest periodic image in simulations with periodic boundary conditions (this can introduce artifacts that do not matter for our simplified toy model).

Repulsion: The purely repulsive particle interaction we use to model the finite size of the superparamagnetic microparticles is implemented via the Weeks-Chandler-Andersen (WCA) potential[S9]

$$V(|\vec{r}_{ij}|) = \begin{cases} 4\varepsilon \left[\left(\frac{\sigma}{|\vec{r}_{ij}|}\right)^{12} - \left(\frac{\sigma}{|\vec{r}_{ij}|}\right)^{6} \right] + \varepsilon, & |\vec{r}_{ij}| \leq 2^{1/6}\sigma \\ 0, & |\vec{r}_{ij}| > 2^{1/6}\sigma, \end{cases} \tag{S11}$$

where ε is the interaction strength. This pair potential is a shifted Lennard-Jones potential that is truncated at its minimum $r = 2^{1/6}\sigma$ to obtain only its repulsive part. The same potential is applied to realize the soft walls[S1, S2]. In fact, even the same interaction strength is inserted compared to the particle-particle interactions.

Poiseuille flow: For our studies with an additional flow (or velocity) field, we add the standard Poiseuille flow to the equations of motion[S4]. In detail, we set the soft wall over the micromagnet array at the position $z = 0$ and complete the Poiseuille flow setup with an additional soft wall with the position $z = h$. Thus, we can write

$$\vec{v}_i^{\mathrm{Poi}}(t) = v_0 \left[1 - \frac{(z_i(t) - h/2)^2}{(h/2)^2} \right] \tag{S12}$$

for the flow velocity affecting the ith particle, where we assume that the two walls act as no-slip boundary conditions (for $\vec{v}_i^{\mathrm{Poi}}$). Here, $z_i(t) = \vec{r}_i(t) \cdot (0,0,1)^{\mathrm{t}}$ is the z-component of the position of sphere i and v_0 is the maximum velocity, which can be matched to generate similar volume flows as in the experiments.

Note that all quantitative flow rate data are always given concerning the setup of the experiments, which possess a width of $3\,\mathrm{mm}$ and a height of approximately $30\,\mu\mathrm{m}$ (which we rounded from the real $25.4\,\mu\mathrm{m}$). This means that the average velocity is matched between the experiments and the simulations, and the flow rate numbers that are given in the text should be interpreted as the value the simulated system would have if it is set with the same dimensions as the real microfluidic channel.

Hydrodynamic interactions: Similar to works such as Ref. S6, we incorporate hydrodynamics on the Oseen level (i.e., we use a point particle model) in our numerical toy model. For this, we adapt the relations that can be

found in the theory part and the appendix of Ref. S3 to our computations. While we neglect the influence of all additional walls that are included during the studies with an additional flow field, we incorporate the effect of the soft walls between the particles and the micromagnet array (at position $z = 0$). Hence, we have to base the grand mobility tensor on the Blake tensor[S3]. In detail, for $i \neq j$, the mobility tensor is identical to said Blake tensor which means

$$\hat{\mu}_{ij} = \hat{G}(\vec{r}_i - \vec{r}_j) - \hat{G}(\vec{r}_i - \vec{r}\,'_j) + \delta\hat{G}(\vec{r}_i, \vec{r}_j), \tag{S13}$$

where $\vec{r}\,'_j = \vec{r}_j - 2\vec{z}_j$ with $\vec{z}_j = \vec{r}_j(t) \cdot (0,0,1)^{\mathrm{t}}$ is the position of a mirror particle behind the wall, and

$$\hat{G}(\vec{r}) = \frac{1}{8\pi\eta|\vec{r}|}\left(\mathbb{1} + \frac{\vec{r}\otimes\vec{r}}{|\vec{r}|^2}\right) \tag{S14}$$

is the Oseen tensor, where η is the viscosity of the dispersion medium, $\mathbb{1}$ is the identity matrix, and $\otimes$ is the dyadic product[S3]. The matrix $\delta\hat{G}(\vec{r}_i, \vec{r}_j)$ is defined by the equations for its components

$$8\pi\eta\,\delta G_{xx} = -2z_i z_j\left[\frac{1}{s^3} - 3\frac{(x_i - x_j)^2}{s^5}\right] \tag{S15}$$

$$8\pi\eta\,\delta G_{yy} = -2z_i z_j\left[\frac{1}{s^3} - 3\frac{(y_i - y_j)^2}{s^5}\right] \tag{S16}$$

$$8\pi\eta\,\delta G_{zz} = 2z_i z_j\left[\frac{1}{s^3} - 3\frac{(z_i + z_j)^2}{s^5}\right] \tag{S17}$$

$$8\pi\eta\,\delta G_{xy} = 6\frac{z_i z_j\,(x_i - x_j)\,(y_i - y_j)}{s^5} = 8\pi\eta\,\delta G_{yx} \tag{S18}$$

$$8\pi\eta\,\delta G_{xz} = 2(x_i - x_j)\left[\frac{z_j}{s^3} - 3\frac{z_i z_j\,(z_i + z_j)}{s^5}\right] \tag{S19}$$

$$8\pi\eta\,\delta G_{zx} = 2(x_i - x_j)\left[\frac{z_j}{s^3} + 3\frac{z_i z_j\,(z_i + z_j)}{s^5}\right] \tag{S20}$$

$$8\pi\eta\,\delta G_{yz} = 2(y_i - y_j)\left[\frac{z_j}{s^3} - 3\frac{z_i z_j\,(z_i + z_j)}{s^5}\right] \tag{S21}$$

$$8\pi\eta\,\delta G_{zy} = 2(y_i - y_j)\left[\frac{z_j}{s^3} + 3\frac{z_i z_j\,(z_i + z_j)}{s^5}\right] \tag{S22}$$

with $\vec{r}_i = (x_i, y_i, z_i)^{\mathrm{t}}$, $\vec{r}_j = (x_j, y_j, z_j)^{\mathrm{t}}$ and $s = |\vec{r}_i - \vec{r}\,'_j|$, which we took out of the appendix of Ref. S3. Note that these relations only hold for a wall at position $z = 0$ and particles with $z_i > 0$.

We complete the grand mobility tensor by defining the remaining components for $i = j$ by

$$\hat{\mu}_{ii} = \frac{D}{k_B T}\mathbb{1} - \hat{G}(\vec{r}_i - \vec{r}\,'_i) + \delta\hat{G}(\vec{r}_i, \vec{r}_i). \tag{S23}$$

The calculation of the hydrodynamic mobility tensor is the bottleneck of the simulations as it approximately scales with N^2, where N is the number of particles. Furthermore, the hydrodynamic interactions are long-ranged and, thus, we cannot introduce a cutoff radius or use similar techniques.

Remarks on the position of the wall: As we use a simplified hydrodynamic point particle model, unphysical artifacts can occur if the microparticles are too close to the wall, which is placed over the micromagents. In detail, the mobility tensor $\hat{\mu}_{ii}$ can change its sign when the corresponding particle penetrates the potential modeling the steric particle-wall interactions too much. To avoid this, we shift the position of the "steric representation of the wall" slightly above the wall we use in the calculation of the Blake tensor. This means that we use a wall positioned at $z = 0$ for the calculation of the Blake tensor, and a wall at $z = \delta z$ for the repulsive particle-wall interactions. One can interpret this approach as a setup, where a small layer is applied on the wall, that repels the microparticles but is perfectly permeable for the surrounding fluid. With this strategy, we avoid the artifacts in the simulations. However, note that tests showed that using $\delta z = 0$ does not affect the general collective dynamics of the system and the same qualitative behavior can be obtained.

General parameters used in the simulations: The simulations possess a large parameter set that we tried to match to the experimental setup as well as possible. Starting with the magnetic particles, we always assume a diameter of $10\,\mu\mathrm{m}$, a density of $1100\,\mathrm{kg/m^3}$ and a magnetization of $1.8\,\mathrm{Am^2/kg}$ (at $H = 80\,\mathrm{kA/m}$) (which corresponds to the properties of the real microparticles). With this, we obtain the magnitude of the magnetic moment $m_0 = 1.296 \times 10^{17}\,\mathrm{m}^3\,|\vec{H}(t)|$.

We chose to match the volume and the aspect ratio of the elliptical micromagnets of the simulations to their experimental counterparts. Hence, we use $V^{\mathrm{pol}} = 15 \times 15 \times 45\,\mu\mathrm{m}^3$ and $q = 3.00$ and find $\alpha^{\mathrm{pol}} = 781.31$. For the susceptibility of the micromagnets, we utilize $\chi_0 = 20$. This value can approximately be obtained from the relative magnetic permeability that was measured via a vibrating sample magnetometer and applied to FEM simulations in Ref. S10 for similar micromagnets. However, note that χ_0 does not influence the simulation distinctly as the apparent susceptibility tensor is dominated by geometric effects for large susceptibility[S8]. For instance, changing χ_0 from $\chi_0 = 20$ to $\chi_0 = 2000$ changes $\chi^{\parallel}$ and $\chi^{\perp}$ only by factors 1.45 and 1.11, respectively.

To maintain numerical stability, we set the interaction strength of the WCA potential to $\varepsilon = 10000 k_B T$. For the temperature, we apply $293.15\mathrm{K}$. If not stated otherwise, we utilize a distance of $60\,\mu\mathrm{m}$ between the micromagnets. The point dipols that resemble the micromagnets are placed $21.0\,\mu\mathrm{m}$ below the wall. This value was also optimized for numerical stability and is only possible as the point dipole micromagnets do not possess a "real" finite volume (the tips of the elliptical micromagnets would slightly poke through the wall). For the shift δz describing the difference between the position of the wall for the steric and the hydrodynamic interactions, we utilize $1.5\,\mu\mathrm{m}$. In the simulations where an additional flow field is applied, we shift the upper wall also by $1.5\,\mu\mathrm{m}$ to maintain the predefined distance between the walls (the Poiseuille flow is applied between $z = 0$ and $z = h\,\mu\mathrm{m}$ nonetheless).

Specific parameters for the different runs: For the runs depicted in Fig. 4 of the main article, we utilize a micromagnet array of 4×100 micromagnets and randomly place 200 particles over the first two rows of said array. The length of the simulation step is set to $2.33 \times 10^{-5}\,\mathrm{s}$ for $18\,\mathrm{mT}$ and $2.33 \times 10^{-6}\,\mathrm{s}$ for $130\,\mathrm{mT}$. To calculate the velocity of the particle front, we define the fourth row of microparticles as a start line and the tenth row of microparticles as the finish line. If the first 20 particles pass the start line, we begin with the velocity calculations. After the first 20 particles pass the finish line, the simulation ends and the velocity can be obtained by the distance between the start and the finish line, as well as the time needed for the particles to travel this distance.

The simulations for the phase diagram in Fig. 5 are implemented analogously. Here, the length of the time step is also set to $2.33 \times 10^{-5}\,\mathrm{s}$ or $2.33 \times 10^{-6}\,\mathrm{s}$ depending on the stability of the simulations. To determine the width of the particle bands and the number of microparticles per occupied micromagnet, we also perform runs that do not end when the particle front reaches the finish line. In these runs, we set the total simulation time of approximately $115\,\mathrm{s}$ and use a time step length of $2.33 \times 10^{-5}\,\mathrm{s}$ throughout. The width of the particle band is defined as the distance between the last two rows of micromagnets where at least 5 microparticles are located (meaning that we compute the total number of all microparticles positioned at the four micromagnets corresponding to a row and take the distance between the first and the last row for which this number is higher or equal to 5). We consider a micromagnet occupied if it has at least a single particle in its vicinity. Formally, a particle is in the vicinity of the micromagnet when its position in the xy plane is in a square around the micromagnet with a box length of $60\,\mu\mathrm{m}$.

For the simulations with a finite flow, we implement a micromagnet array of 60×20 micromagnets and utilize a time step length of $5.82 \times 10^{-6}\,\mathrm{s}$. The height of the channel is chosen to be $h = 30\,\mu\mathrm{m}$. Initially, 60 particles are randomly placed at one of the boundaries perpendicular to the flow field. Then, new particles spawn after a specific number of steps. At $12.4\,\mu\mathrm{L/min}$, $21.6\,\mu\mathrm{L/min}$, and $37.1\,\mu\mathrm{L/min}$, a new particle appears after every 230, 132, and 76 simulation steps, respectively. Here, every simulation runs for 10^6 simulation steps each but we manually stop them after the separation line has fully formed. For supporting simulations analyzing the influence of the channel dimensions, we vary the height of the channel between $30\,\mu\mathrm{m}$ and $120\,\mu\mathrm{m}$. The spawn rate of the particles is adjusted for the increased volume.

Notes on gravity: Throughout all simulations, we neglect the influence of gravity on the particle dynamics. This can be justified by approximating estimates for the corresponding strengths of the magnetic and gravitational forces. For this, we assume a density of the colloids of $\rho = 1100\,\mathrm{kg/m^3}$ and, additionally, take the buoyancy (which effectively reduces the influence of the gravity) into account. The resulting reduced force of gravity is roughly

$$F_G = (\rho - \rho_{\mathrm{water}})V^{\mathrm{col}} g \approx 1300\,k_B T/\sigma \approx 0.51\,\mathrm{pN}, \tag{S24}$$

where ρ_{water} is the density of water, $V^{\mathrm{col}} = 4/3\pi(\sigma/2)^3$ is the volume of the spherical colloids, and g is the acceleration corresponding to gravity. We can compare this value with the magnitude of the magnetic interaction force $\vec{F}_{ij}$ between an isolated colloid and an isolated micromagnet. Let us consider an example case with a low magnetic field with a strength of $18\,\mathrm{mT}$ (which is the lowest field used in the numerical investigations). For simplicity, we further assume

that $\vec{m}^{col} \parallel \vec{m}^{pol}$, $\hat{\chi} \approx \mathbb{1}$, and $\vec{r}_{ij} \parallel \vec{m}^{col}$. With this, we find

$$|\vec{F}_{ij}| \approx 2F_0^{\mathrm{mag}} \alpha^{\mathrm{pol}} \left(\frac{\sigma}{|\vec{r}_{ij}|}\right)^4 \approx 4.0 \times 10^6\, k_B T/\sigma \left(\frac{\sigma}{|\vec{r}_{ij}|}\right)^4 \approx 1.62\,\mathrm{nN} \left(\frac{\sigma}{|\vec{r}_{ij}|}\right)^4 \tag{S25}$$

from Eq. (S9). Thus, it becomes clear that F_G is much smaller than $|\vec{F}_{ij}|$, and it is reasonable to neglect its influence at distances that are relevant to our simulations. For instance, $|\vec{F}_{ij}| \approx F_G$ at $|\vec{r}_{ij}| \approx 7.5\,\sigma = 75.0\,\mu\mathrm{m}$, which is more than twice the height of the channel used in the experiments. Subtracting the distance $75.0\,\mu\mathrm{m}$ of the micromagnets from the bottom wall (as they are placed behind the confining boundary), we obtain a height interval of $54.0\,\mu\mathrm{m}$ in respect to the bottom wall, where the influence of the gravity is smaller than the influence of the analyzed dipole interactions. Note that F_0^{mag} scales with $m_0^2 \sim H^2$ (see Eq. (S10)). This means that the difference between the orders of magnitude of the magnetic and gravitational forces becomes even much more distinct for larger magnetic field strengths.

Comments on the quantitative differences between the experiments and the simulations: Looking at Fig. 4B of the main article, the general results of the experiments and the many-particle simulations agree qualitatively: Up to a certain transition frequency, the collective dynamics are synchronous with the external magnetic field, meaning that the velocity of the particle front follows a linear relation. Passing the transition frequency, this linear relation between the velocity and the frequency is broken, and, finally, the particle front velocity decays.

Nevertheless, a clear quantitative deviation between the experimental and numerical results can be seen in Fig. 4B. Many aspects could be responsible for or could influence this deviation: To correctly assess the differences visible in Fig. 4B, one should keep in mind that the simulation results are based on simplified calculations with a model system consisting of 200 colloids. On the other hand, the experiments correspond to distinctly larger systems of much more microparticles. This should influence the results. Secondly, in Fig. 4D, it is visible that there is particle content at the micromagnets even before the particle band has reached them. As we discuss in Sec. "Characterization of collective transport" of the main article, we expect that a certain number of colloids must be assembled at a micromagnet so that jumps are still possible. Hence, the particle content that is already present on the micromagnets could influence the transition frequency because it could push the particle number of the "critical number of colloids" that is needed to keep the transport more synchronous and increase the observed particle front velocity. The last and most important aspect is that the presented many-particle simulations are a simple toy model that captures the essential collective dynamics of the complex experimental system (see above for the multiple simplifications that are made in the numerical calculations). Hence, the simulation results should strictly be interpreted on a qualitative level.

Remarks regarding the visualization: All images depicting the numerical result in the main article and all supplemental movies corresponding to the simulations are done using Visual molecular dynamics (VMD)[S11].

Source code availability: The code of the performed many-particle simulations is available via GitHub: `https://github.com/AntonLueders/BDHM`. This repository also contains the CAD data for the 3D-printed parts of the experimental setup. The corresponding permanent DOI is 10.5281/zenodo.10982927.

Note S9 How to design the micromagnetic array and choose system parameters?

In general, design parameters will strongly depend on the specific application, especially considering the complex collective dynamics of the magnetic microparticles. Here, we would like to provide a general guideline on how to design one's own high-throughput systems based on our proposed scalable method. And share our insights on the gains and compromises in choosing certain parameters to maximize the final results balanced between throughput and reliability. The discussions are based on the simple system we presented in the paper (micrometer-sized magnetic particles and water).

Step 1: Choose magnetic microparticles: For most applications, there are limited choices for magnetic particles with the desired surface coating and size polydispersity. However, with advances in magnetic particle synthesis, it is possible to create your own magnetic microparticles with designer surface materials and functionalities.

Size selection: Magnetic particles need to be large enough to provide sufficient magnetic moment that scales with volume (L^3), however, one advantage of using magnetic particles is the high surface-to-volume ratio, and making the size L too large will compromise the total surface area ($\sim L^2$). Another issue related to size is Brownian motion, as thermal energy may become more pronounced at small sizes and the dynamics we observed in the paper may break down at the nanoscale.

Magnetic properties: Magnetic microparticles should have sufficient magnetic moments to interact strongly with the micromagnet array to move efficiently through the flow. We would say that the magnetic particles should be as permeable as possible. In addition, we would recommend that the magnetic moment of the microparticles have a linear dependence on the external magnetic field. The use of high coercivity magnetic materials will have its own magnetic memory, which will significantly increase the complexity of the particle dynamics (some aspects are discussed in supplemental Information Section S10 Magnetic properties of the micromagnet array and magnetic microparticles). This limits the choice of materials to superparamagnetic particles or soft magnetic materials.

Surface properties: The surface materials are usually determined by the intended applications (valuable catalyst, antigen for biomedical applications, etc.). However, dispersion and mixing with the media are also important. For water-based media, the surface of the microparticles must be hydrophilic so that they can be easily dispersed and react sufficiently in the media. If the surface is hydrophobic, the particle may want to cluster and compromise its advantages in a high surface-to-volume ratio and reaction time may increase in the first step before particle separation.

Cost: Since we are considering very high throughput applications (> 100 mL/min), the cost of the process can increase quickly and it is important to keep the price low or find a way to recycle and reuse the microparticles.

Further analysis and post processing: After the particles are separated from the bulk liquid, some applications require further treatment, and users need to consider whether the particles are compatible with these processes. For example, proteins or cells captured by the particles may need to be further analyzed to determine other properties. In another example, valuable catalysts may need to be recycled, cleaned, and reused.

Step 2: Micromagnet chip design: Our presented fabrication method based on 1-layer photolithography and electroplating supports a wide range of customized designs of micromagnet chips at reasonable cost.

Periodicity of micromagnet array: The periodicity of the micromagnet array should match the designed cluster size, which is a result of balancing transport speed and robustness. If one has a designed cluster size (100 microparticles), the periodicity of the micromagnet array should be about 1.5 to 2 times the size of the clusters.

We numerically study the influence on the periodicity by changing the periodicity of the micromagnetic array while keeping the rest of the parameters constant ($B = 18$ mT @ 1Hz). As shown in Fig. S5, we can clearly identify there is an optimum for a given magnetic field strength and rotating frequency. However, the distribution of the swarm of microparticles also needs to be considered since it is a sign of transport robustness.

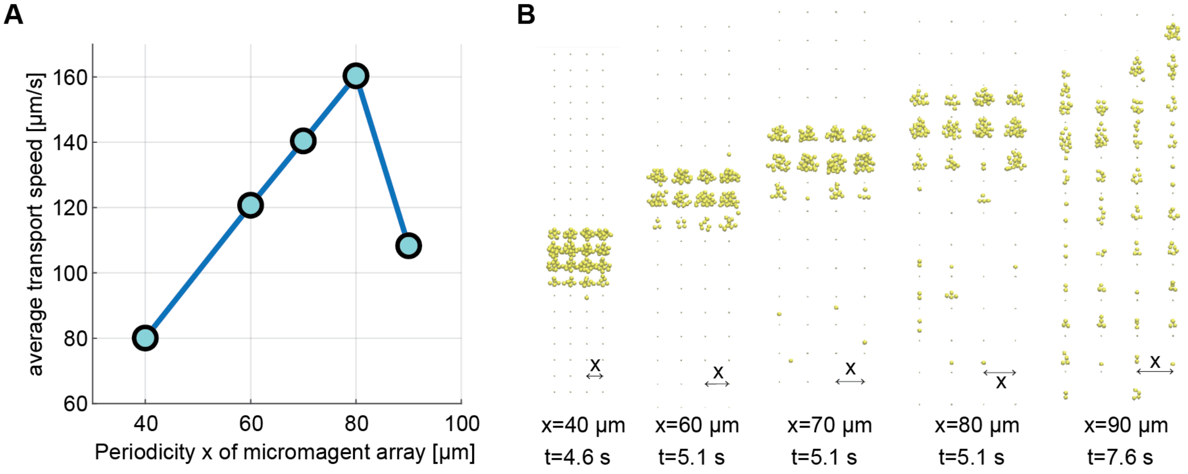

Figure S5. The influence of periodicity on the transport performance of swarm magnetic microparticles. A.) The average transport speed of magnetic microparticle swarms under the condition of an 18 mT rotating magnetic field at 1 Hz. B.) The morphologies of the swarm distributed on the micromagnetic array (top view) at the end of the numerical simulations.

Length and width of each micromagnet: Each micromagnet should have a long axis perpendicular to the chip substrate, and to prevent the magnetic coupling between the neighboring micromagnets, so the length and width of the micromagnet should be a small fraction of the periodicity L, a typical reference value should be $a, b < 0.3\ L$.

Height of the micromagnet: Increasing the height can ensure the magnetic anisotropy so that the long axis is always perpendicular to the chip. We recommend that the height is two times or more compared to the length and width (in our micromagnet array, the height is about 45 micrometers and the width is 15 micrometers). In

addition, increasing the height will increase the magnetic volume of the micromagnet, effectively increasing the magnetic attraction and promoting particle transport. However, increasing the height also increases the aspect ratio in microfabrication, which can be challenging and lowers the microfabrication yield rate.

Step 3: Design individual microfluidic channels: *Channel length and width:* First, the microfluidic channel should be long enough to allow all the magnetic microparticles to move across the flow and accumulate at one end of the microfluidic channel. This includes both the magnetic particle assembly process and the active swarming motion across the flow as shown in Figure 4A. In addition, the length and width should be adjusted to allow for the separation line across the channel, which is determined by the pumping speed of the externally driven flow and the collective moving speed of the magnetic microparticles.

Height of the channel: The height of the channel should have a characteristic length as the height of the micromagnet. The local gradient field, which is responsible for attracting free-floating microparticles in the liquid to the vicinity of the micromagnets, decays rapidly when moving away from the chip surface. This means that if the microfluidic channel is too high, particles at higher positions can be flushed away before they are attracted by the micromagnets and moved along with other particles. On the other hand, if the channel is too low, the flow resistance increases, and the strong shear flow profile can cause problems in the blood, as it can cause coagulation and thrombosis.

Step 4: Designing the rotating magnetic field setup and microfluidic networks: After determining the system parameters of the micromagnetic chip and microfluidic channels, it is time to consider how to scale up such a device structure in parallel to increase the throughput of the whole system. The design of the rotating magnetic field setup will provide us with sufficient working space that matches the required magnetic field strength. It is an optimization problem to provide the maximum workspace at a given cost for the NdFeB magnet. As shown in Fig. S7, our proposed design with a motor setup can be easily scaled in parallel with minimal interference between the rotating magnets.

The next step is to design the microfluidic networks. In the basic case of scaling in parallel, the microfluidic network can be embedded inside the 3D printed case, but the optimal connection architecture (parallel, hierarchical, etc.) is still unknown. In some cases, the suspensions may need to pass through three chips to achieve the best separation results. These issues are beyond the scope of this work and require further investigation in future work.

Step 5: Optimize operating frequency and flow rate: After the device is fully assembled, it is still possible to tune system parameters including the rotating frequencies, the pumping rate of particle suspensions, and the relative flow rate between the “clean” liquid channel and the recycled microparticle channel. Such parameters may require constant adjustment to match the dynamic situations in the separation, e.g., varying microparticle density.

In practice, one needs to decide the magnetic field strength threshold in the system (this determines the overall workspace) and the operating frequency of the rotating magnetic field. As shown in the Fig. S6. If one uses a low magnetic field threshold B1, one can in principle use a larger working volume, but this means the capped rotating frequency is low. On the other hand, if one chooses a higher magnetic field threshold, the corresponding usable working volume is smaller, but one can push up the rotating frequency to achieve faster particle speed. The particle rotating speed and the working volume together determine the throughput of the device. And we expect details may vary across different microparticles and applications.

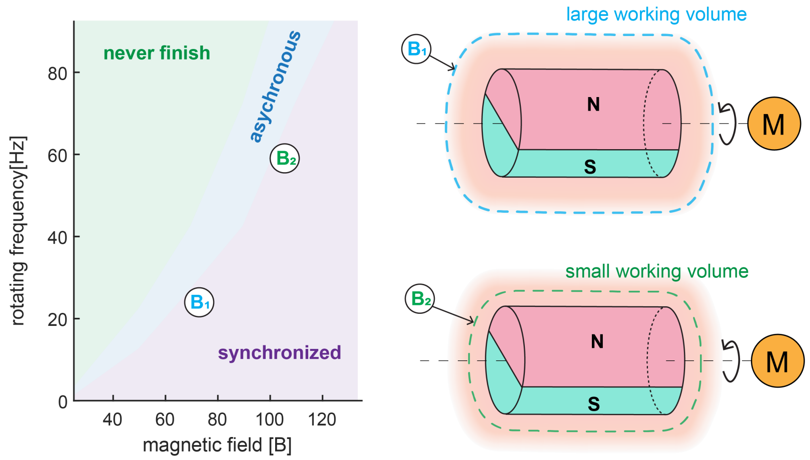

Figure S6. Compromises between magnetic field strength, workspace, and rotating frequency.

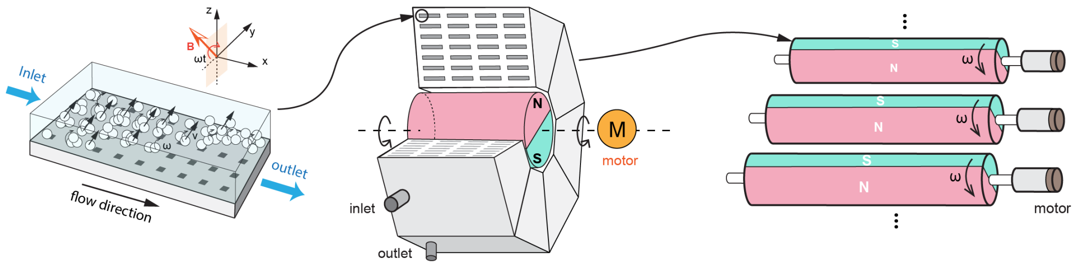

Design stage	Individual microfluidic device design (step 1-3)	Microfluidic architecture design (step 4)	Scalable system and operating parameter optimziaiton (step 5)
system parameters	magnetic particle<br>micromagnet chip design<br>rotating magnetic field	number of chips<br>fluidic network	operating frequency ω<br>pumping flow rate
dimension	10x3x1 mm<br>(single channel pack)	magnet: 3(D)x5(L) cm<br>case: 10(D)x5(L) cm	20x20x40 cm<br>(include 8 motors)
throughput	~0.1ml/min	~50 ml/min	~400 ml/min

Figure S7. Design process of scalable high-throughput magnetic particle separation system. It provides a map of key parameters at different design stages, and shows how the system can be further scaled to 100mL/min and more, even though it is not demonstrated experimentally in this work.

Note S10 Magnetic properties of the micromagnet array and magnetic microparticles

Magnetic properties of the micromagnets: Micromagnets must be dynamically magnetized by the rotating magnetic field. When the magnetic field is along the long axis of the micromagnets (perpendicular to the microchip), the micromagnets attract the surrounding particles (shown in red in Fig. 2 in Ref. S10), and when the magnetic field is along the short axis of the micromagnets (parallel to the microchip), the surrounding particles are repelled (shown in blue in Fig. 2 in Ref. S10). This alternating "attract and repel" behavior is critical for the microparticles to successfully move forward to the next micromagnet.

This feature determines that the micromagnets have a very small coercivity that the external rotating field can dynamically magnetize, demagnetize, and remagnetize the micromagnets. Soft magnetic materials (ferromagnetic materials with a small narrow hysteresis loop) are good choices. We choose electroplated multi-domain nickel in our fabrication methods because of its simplicity, low cost, and ease of scale-up. High permeability is also desirable as it can provide a higher local gradient field.

Another type of material we think is possible is superparamagnetic materials. They also have very low (almost zero) coercivity and high permeability. (e.g. iron oxide nanoparticles).

Magnetic properties of mobile microparticles: Unlike micromagnets, mobile particles can have a wide range of magnetic properties as long as they are strongly attracted to the magnetic field. This means that diamagnetic materials are not suitable. In this work, we mainly focus on the polymer-based particle with embedded iron oxide nanoparticles, which exhibit superparamagnetic behavior. This is due to the fact that the dynamics are easier to study because the particles have no memory for previously applied magnetic fields, and the magnetic coupling between neighboring magnetic particles is negligible because the nanoparticles are sufficiently separated.

We also have performed experiments with particles having different magnetic properties. For virgin NdFeB microparticles (average size: 5 micrometers), the particles are gradually magnetized and have their own magnetizations, which cannot be synchronized with the rotating magnetic field due to the high coercivity. As a result, the particles form larger and larger clusters (as shown in Fig. S8). This effect is not irreversible and the dynamics and behavior are difficult to predict.

We also performed some experiments with pure iron oxide nanoparticles (size: 30 nm). As shown in Fig. S9, the nanoparticles form very large clusters (like a cylinder) and tumble on the micromagnet chip. Due to the strong magnetic interaction between nanoparticles, the particles do not disassemble when the field is turned off. And the particles do not disperse well because of their nanometer size. So we think it may have limitations for certain applications.

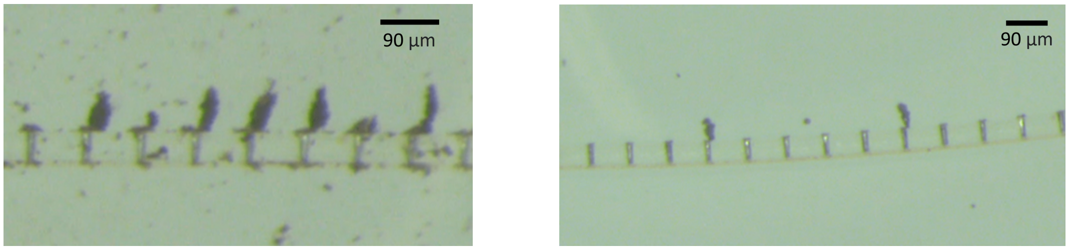

A B

Figure S8. Transport of ferromagnetic NdFeB microparticles on the 1-D micromagnet array. Two views (panel A and B) are presented depending on the local magnetic particle density.

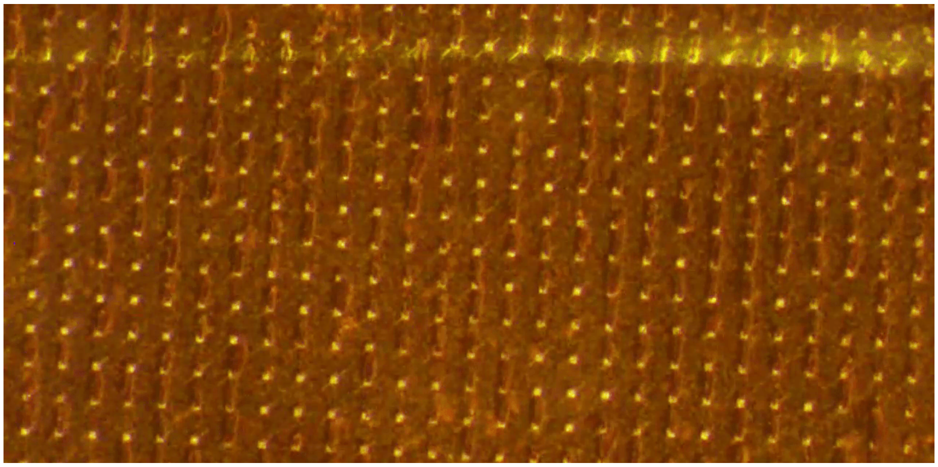

Figure S9. Iron oxide nanoparticles (average size: 30 nm) assemble into large clusters and are transported on the micromagnet array (periodicity: 120 micrometers). A rotating magnetic field of 40 mT is applied. The video is added as supplemental Movie S8.

References

[S1] Lüders, A., Siems, U. and Nielaba, P. (2019). Dynamic ordering of driven spherocylinders in a nonequilibrium suspension of small colloidal spheres. Phys. Rev. E . 99, 022601. 10.1103/PhysRevE.99.022601.

[S2] Stuckert, R., Lüders, A., Wittemann, A. and Nielaba, P. (2021). Phase behaviour in 2D assemblies of dumbbell-shaped colloids generated under geometrical confinement. Soft Matter 17, 6519–6535. 10.1039/D1SM00635E.

[S3] Jones, R. B. and Kutteh, R. (1999). Sedimentation of colloidal particles near a wall: Stokesian dynamics simulations. Phys. Chem. Chem. Phys. 1, 2131–2139. 10.1039/A809571J.

[S4] Bhattacharya, S., Bławzdziewicz, J. and Wajnryb, E. (2006). Hydrodynamic interactions of spherical particles in poiseuille flow between two parallel walls. Phys. Fluids 18, 053301. 10.1063/1.2195992.

[S5] Carrasco, B. and García de la Torre, J. (1999). Hydrodynamic properties of rigid particles: comparison of different modeling and computational procedures. Biophys. J. 76, 3044. 10.1016/S0006-3495(99)77457-6.

[S6] Granados Leyva, S., Stoop, R., Pagonabarraga, I. and Tierno, P. (2022). Hydrodynamic synchronization and clustering in ratcheting colloidal matter. Sci. advances 8, eabo4546. 10.1126/sciadv.abo4546.

[S7] Sing, C., Schmid, L., Schneider, M., Franke, T. and Alexander-Katz, A. (2010). Controlled surface-induced flows from the motion of self-assembled colloidal walkers. Proc. Natl. Acad. Sci. United States Am. 107, 535–40. 10.1073/pnas.0906489107.

[S8] Abbott, J. J., Ergeneman, O., Kummer, M. P., Hirt, A. M. and Nelson, B. J. (2007). Modeling magnetic torque and force for controlled manipulation of soft-magnetic bodies. IEEE Trans. Robot. 23, 1247–1252. 10.1109/TRO.2007.910775.

[S9] Weeks, J. D., Chandler, D. and Andersen, H. C. (1971). Role of repulsive forces in determining the equilibrium structure of simple liquids. J. Chem. Phys. 54, 5237–5247. 10.1063/1.1674820.

[S10] Gu, H., Hanedan, E., Boehler, Q., Huang, T.-Y., Mathijssen, A. J. and Nelson, B. J. (2022). Artificial microtubules for rapid and collective transport of magnetic microcargoes. Nat. Mach. Intell. 4, 678–684. 10.1038/s42256-022-00510-7.

[S11] Humphrey, W., Dalke, A. and Schulten, K. (1996). VMD: visual molecular dynamics. J. Mol. Graph. 14, 33–38. 10.1016/0263-7855(96)00018-5.
